# The complete reference genome for grapevine (*Vitis vinifera* L.) genetics and breeding

**DOI:** 10.1093/hr/uhad061

**Published:** 2023-04-04

**Authors:** Xiaoya Shi, Shuo Cao, Xu Wang, Siyang Huang, Yue Wang, Zhongjie Liu, Wenwen Liu, Xiangpeng Leng, Yanling Peng, Nan Wang, Yiwen Wang, Zhiyao Ma, Xiaodong Xu, Fan Zhang, Hui Xue, Haixia Zhong, Yi Wang, Kekun Zhang, Amandine Velt, Komlan Avia, Daniela Holtgräwe, Jérôme Grimplet, José Tomás Matus, Doreen Ware, Xinyu Wu, Haibo Wang, Chonghuai Liu, Yuling Fang, Camille Rustenholz, Zongming Cheng, Hua Xiao, Yongfeng Zhou

**Affiliations:** College of Horticulture, Qingdao Agricultural University, Qingdao 266109, China; State Key Laboratory of Tropical Crop Breeding, Shenzhen Branch, Guangdong Laboratory of Lingnan Modern Agriculture, Key Laboratory of Synthetic Biology, Ministry of Agriculture and Rural Affairs, Agricultural Genomics Institute at Shenzhen, Chinese Academy of Agricultural Sciences, Shenzhen 518120, China; State Key Laboratory of Tropical Crop Breeding, Shenzhen Branch, Guangdong Laboratory of Lingnan Modern Agriculture, Key Laboratory of Synthetic Biology, Ministry of Agriculture and Rural Affairs, Agricultural Genomics Institute at Shenzhen, Chinese Academy of Agricultural Sciences, Shenzhen 518120, China; Key Laboratory of Horticultural Plant Biology Ministry of Education, College of Horticulture and Forestry Sciences, Huazhong Agricultural University, Wuhan 430070, China; State Key Laboratory of Tropical Crop Breeding, Shenzhen Branch, Guangdong Laboratory of Lingnan Modern Agriculture, Key Laboratory of Synthetic Biology, Ministry of Agriculture and Rural Affairs, Agricultural Genomics Institute at Shenzhen, Chinese Academy of Agricultural Sciences, Shenzhen 518120, China; School of Agriculture and Food Science, University College Dublin, Belfield, Dublin 4, Ireland; State Key Laboratory of Tropical Crop Breeding, Shenzhen Branch, Guangdong Laboratory of Lingnan Modern Agriculture, Key Laboratory of Synthetic Biology, Ministry of Agriculture and Rural Affairs, Agricultural Genomics Institute at Shenzhen, Chinese Academy of Agricultural Sciences, Shenzhen 518120, China; National Demonstration Center for Experimental Plant Science Education, College of Agriculture, Guangxi University, Nanning 530004, China; State Key Laboratory of Tropical Crop Breeding, Shenzhen Branch, Guangdong Laboratory of Lingnan Modern Agriculture, Key Laboratory of Synthetic Biology, Ministry of Agriculture and Rural Affairs, Agricultural Genomics Institute at Shenzhen, Chinese Academy of Agricultural Sciences, Shenzhen 518120, China; State Key Laboratory of Resource Insects, Southwest University, Chongqing 400715, China; State Key Laboratory of Tropical Crop Breeding, Shenzhen Branch, Guangdong Laboratory of Lingnan Modern Agriculture, Key Laboratory of Synthetic Biology, Ministry of Agriculture and Rural Affairs, Agricultural Genomics Institute at Shenzhen, Chinese Academy of Agricultural Sciences, Shenzhen 518120, China; State Key Laboratory of Tropical Crop Breeding, Shenzhen Branch, Guangdong Laboratory of Lingnan Modern Agriculture, Key Laboratory of Synthetic Biology, Ministry of Agriculture and Rural Affairs, Agricultural Genomics Institute at Shenzhen, Chinese Academy of Agricultural Sciences, Shenzhen 518120, China; College of Horticulture, Qingdao Agricultural University, Qingdao 266109, China; State Key Laboratory of Tropical Crop Breeding, Shenzhen Branch, Guangdong Laboratory of Lingnan Modern Agriculture, Key Laboratory of Synthetic Biology, Ministry of Agriculture and Rural Affairs, Agricultural Genomics Institute at Shenzhen, Chinese Academy of Agricultural Sciences, Shenzhen 518120, China; State Key Laboratory of Tropical Crop Breeding, Shenzhen Branch, Guangdong Laboratory of Lingnan Modern Agriculture, Key Laboratory of Synthetic Biology, Ministry of Agriculture and Rural Affairs, Agricultural Genomics Institute at Shenzhen, Chinese Academy of Agricultural Sciences, Shenzhen 518120, China; State Key Laboratory of Tropical Crop Breeding, Shenzhen Branch, Guangdong Laboratory of Lingnan Modern Agriculture, Key Laboratory of Synthetic Biology, Ministry of Agriculture and Rural Affairs, Agricultural Genomics Institute at Shenzhen, Chinese Academy of Agricultural Sciences, Shenzhen 518120, China; State Key Laboratory of Tropical Crop Breeding, Shenzhen Branch, Guangdong Laboratory of Lingnan Modern Agriculture, Key Laboratory of Synthetic Biology, Ministry of Agriculture and Rural Affairs, Agricultural Genomics Institute at Shenzhen, Chinese Academy of Agricultural Sciences, Shenzhen 518120, China; State Key Laboratory of Tropical Crop Breeding, Shenzhen Branch, Guangdong Laboratory of Lingnan Modern Agriculture, Key Laboratory of Synthetic Biology, Ministry of Agriculture and Rural Affairs, Agricultural Genomics Institute at Shenzhen, Chinese Academy of Agricultural Sciences, Shenzhen 518120, China; State Key Laboratory of Tropical Crop Breeding, Shenzhen Branch, Guangdong Laboratory of Lingnan Modern Agriculture, Key Laboratory of Synthetic Biology, Ministry of Agriculture and Rural Affairs, Agricultural Genomics Institute at Shenzhen, Chinese Academy of Agricultural Sciences, Shenzhen 518120, China; State Key Laboratory of Tropical Crop Breeding, Shenzhen Branch, Guangdong Laboratory of Lingnan Modern Agriculture, Key Laboratory of Synthetic Biology, Ministry of Agriculture and Rural Affairs, Agricultural Genomics Institute at Shenzhen, Chinese Academy of Agricultural Sciences, Shenzhen 518120, China; Institute of Horticulture Crops, Xinjiang Academy of Agricultural Sciences, Urumqi 830091, China; Beijing Key Laboratory of Grape Science and Enology, Institute of Botany, Chinese Academy of Sciences, Xiangshan, Beijing 100093, China; College of Enology, Northwest A&F University, Yangling 712100, China; SVQV, INRAE - University of Strasbourg, 68000 Colmar, France; SVQV, INRAE - University of Strasbourg, 68000 Colmar, France; Genetics and Genomics of Plants, CeBiTec & Faculty of Biology, Bielefeld University, 33615 Bielefeld, Germany; Unidad de Hortofruticultura, Centro de Investigación y Tecnología Agroalimentaria de Aragón (CITA), 50059 Zaragoza, Spain; Institute for Integrative Systems Biology (I2SysBio), Systems Biotech Program, Universitat de València-CSIC, Paterna, 46908, Valencia, Spain; Cold Spring Harbor Laboratory, Cold Spring Harbor, NY 11724, USA; USDA ARS NEA Robert W. Holley Center for Agriculture and Health, Agricultural Research Service, Ithaca, NY 14853, USA; Institute of Horticulture Crops, Xinjiang Academy of Agricultural Sciences, Urumqi 830091, China; Fruit Research Institute, Chinese Academy of Agricultural Sciences/Key Laboratory of Biology and Genetic Improvement of Horticultural Crops (Germplasm Resources Utilization), Ministry of Agriculture/Key Laboratory of Mineral Nutrition and Fertilizers Efficient Utilization of Deciduous Fruit Tree, Liaoning Province, Xingcheng 125100, China; Zhengzhou Fruit Research Institute, Chinese Academy of Agricultural Sciences, Zhengzhou 450004, China; College of Enology, Northwest A&F University, Yangling 712100, China; SVQV, INRAE - University of Strasbourg, 68000 Colmar, France; College of Horticulture, Nanjing Agricultural University, Nanjing, 210095, China; State Key Laboratory of Tropical Crop Breeding, Shenzhen Branch, Guangdong Laboratory of Lingnan Modern Agriculture, Key Laboratory of Synthetic Biology, Ministry of Agriculture and Rural Affairs, Agricultural Genomics Institute at Shenzhen, Chinese Academy of Agricultural Sciences, Shenzhen 518120, China; Institute of Horticulture Crops, Xinjiang Academy of Agricultural Sciences, Urumqi 830091, China; State Key Laboratory of Tropical Crop Breeding, Shenzhen Branch, Guangdong Laboratory of Lingnan Modern Agriculture, Key Laboratory of Synthetic Biology, Ministry of Agriculture and Rural Affairs, Agricultural Genomics Institute at Shenzhen, Chinese Academy of Agricultural Sciences, Shenzhen 518120, China; State Key Laboratory of Tropical Crop Breeding, Tropical Crops Genetic Resources Institute, Chinese Academy of Tropical Agricultural Sciences, Haikou 571101, China

## Abstract

Grapevine is one of the most economically important crops worldwide. However, the previous versions of the grapevine reference genome tipically consist of thousands of fragments with missing centromeres and telomeres, limiting the accessibility of the repetitive sequences, the centromeric and telomeric regions, and the study of inheritance of important agronomic traits in these regions. Here, we assembled a telomere-to-telomere (T2T) gap-free reference genome for the cultivar PN40024 using PacBio HiFi long reads. The T2T reference genome (PN_T2T) is 69 Mb longer with 9018 more genes identified than the 12X.v0 version. We annotated 67% repetitive sequences, 19 centromeres and 36 telomeres, and incorporated gene annotations of previous versions into the PN_T2T assembly. We detected a total of 377 gene clusters, which showed associations with complex traits, such as aroma and disease resistance. Even though PN40024 derives from nine generations of selfing, we still found nine genomic hotspots of heterozygous sites associated with biological processes, such as the oxidation–reduction process and protein phosphorylation. The fully annotated complete reference genome therefore constitutes an important resource for grapevine genetic studies and breeding programs.

## Introduction

Since the first human genome was published in 2000, hundreds of reference genomes have successively been assembled in a variety of species [1–3]. A reference genome is essential for biological and genetic studies. Thus, acquiring a high-quality genome has persistently been pursued. Despite this, there are many missing segments due to highly repetitive sequences clustered across the genome, especially three representative regions: telomere, centromere, and ribosome DNA (rDNA) [3–5].

The centromere, which hosts CENPA/CENH3-variant nucleosomes and where the kinetochore forms and attaches to spindle microtubules, plays an essential role during cell division. It consists of alpha satellites, highly repetitive DNA sequences. The alpha satellite is composed of monomeric DNA repeats known as higher-order repeats (HORs), which contain arranged monomers that range from 100 to 200 bp [6–8]. Despite their conserved function across species, their structure and sequence can change rapidly within and between species, and diverse organizations can be observed from one species to another. Nevertheless, centromeres show concerted evolution within genomes [7, 9–11]. Currently, the centromere remains mostly unknown to researchers.

Telomeres are mostly unknown as well. They are composed of tandem repeats of relatively conserved microsatellite sequences located at the ends of chromosomes in eukaryotes [12, 13]. Telomeres are important for protecting chromosome terminal sequences during cell division [14–17]. Ribosomal DNA (rDNA) is one of the most abundant repetitive elements in a genome, and plays an essential role in ribosome formation while driving cell growth and cell proliferation [18–20].

Because of the missing information on previously assembled genomes, the investigation of centromeres, telomeres, and rDNA has been extremely limited in the past two decades. Fortunately, benefiting from the improvement of sequencing technologies and computational algorithms, genome assembly has ushered in a new era: that of telomere-to-telomere (T2T) sequencing [21]. Compared with fragmented genomes, a T2T genome has fewer or no gaps at all. It is based on third-generation sequencing platforms, including PacBio high-fidelity long reads (HiFi), ultra-long Oxford Nanopore Technologies (ONT), and Hi-C data. Moreover, the T2T genome includes nearly complete information on the telomere, centromere, and rDNA regions [22, 23]. Promisingly, the T2T genome allows us to access these regions, opening a window into understanding the structure of these regions and the function of genes in these regions. Since the first complete human X chromosome was published in 2020, T2T assembly has quickly become a research hotspot [22, 23]. In plants, the first T2T genome was reported in *Arabidopsis thaliana* in 2021 [7, 24]. At present, T2T genome assemblies have been obtained in several species, such as rice, banana, and watermelon, fascinating researchers into genomic structure and function and their relation to crop breeding traits [25–28].

The grapevine (*Vitis vinifera* ssp. *vinifera*), a fruit tree that originated in the Near East, is one of the most widely cultivated and economically valuable crops worldwide [29]. Domesticated grapes often have highly heterozygous genomes [30], which greatly impedes the acquisition of high-quality genomes. For instance, ~15% of genes are hemizygous in the ‘Chardonnay’ genome.^31^ Fortunately, the PN40024 genotype, a highly homozygous cultivar derived from selfing of cv. ‘Helfensteiner’ [31], became the reference genome of grapevine, first obtained in 2007 (8X), and was the first fruit crop to be sequenced [32]. Subsequently, several updated versions have been released: the 12X.v2 version and its upgraded annotation VCost.v3 in 2017, and the PN40024.v4.1 version in 2021 [33]. The grape gene reference catalogue now includes a full correspondence between all of their annotation versions [34]. In addition, fragmented genome assemblies of various grape cultivars have been produced in recent years, such as those for ‘Black Corinth’ [35], ‘Cabernet Franc’ [36, 37], ‘Cabernet Sauvignon’ [37–39], ‘Carménère’ [40], ‘Chardonnay’ [30, 41], ‘Merlot’ [35], and ‘Nebbiolo’ [42]. As the grapevine is a representative dicotyledonous plant among fruit trees, its high-quality genome will greatly facilitate research on gene function, genetic structure, and evolution of *Vitis* and eudicot species.

Despite the great number of grape genome sequences available, these genome assemblies are incomplete in repetitive regions, centromeres, and telomeres. Here we generated a T2T-level gap-free grape genome of the PN40024 reference and aimed to address four main analyses. The application of third-generation sequencing and assembly technologies to high-fidelity long reads has contributed to gap-free genome assemblies [43, 44]. Thus, our first question was to see whether we could complete the grape reference genome using these new sequencing and assembly approaches. Second, as studies on the centromere, telomere, and rDNA have long been neglected, we analyzed the features, structure, and distribution of these regions based on the assembled gapless grape genome. Third, the annotation of transposable elements (TEs) and genes in highly repetitive regions was improved based on the T2T genome, which could further improve our understanding of their biological functions, especially those of gene clusters. Finally, the PN40024 genome is almost fully homozygous [32], but some sites remain heterozygous after nine generations of selfing. It is worthwhile to investigate the genomic distribution and genetic effects of such heterozygous sites.

## Results

### A telomere-to-telomere gap-free reference genome for grapevine

**Figure 1:**
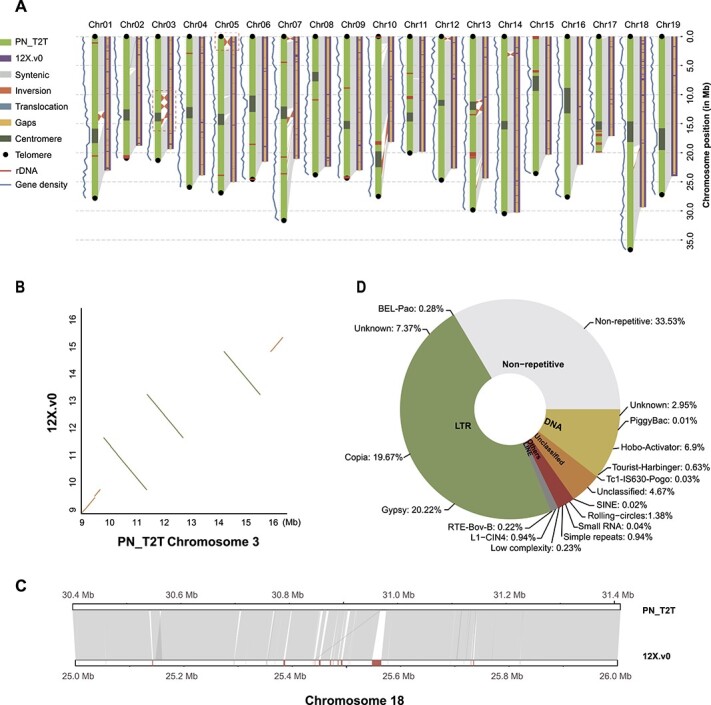
The T2T gap-free assembly of the grapevine reference genome. (**A**) Overview of the genome assemblies (12X.v0, right bars; PN_T2T, left bars). The red dashed boxes on chromosomes 3 and 5 indicate differences in large inversions between the two versions of the genomic assembly. (**B**) Zoomed-in portion of the red dashed box region on chromosome 3 in (**A**). (**C**) Plot showing 1-Mb syntenic region between the 12X.v0 and PN_T2T assemblies on chromosome 18. Gray bands connect corresponding collinear regions, and red boxes at the bottom show the gaps in 12X.v0. (**D**) Types and percentages of different TE families detected in the PN_T2T genome.

PN40024, a highly homozygous inbred line originating from ‘Helfensteiner’, was used for T2T genome assembly. In total, 21 Gb (21 024 461 524 bp, ~42× coverage) HiFi reads were generated by the PacBio platform. For the preliminary assembly, hifiasm was used to assemble the HiFi reads. We then used MUMmer and the 12X.v0 genome version (*V. vinifera* genome assembly 12X.v0; https://www.ncbi.nlm.nih.gov/data-hub/genome/GCF_000003745.3) to order the 38 contigs into 19 chromosomes (Fig. 1). Only one gap was left after initial assembly into contigs (Supplementary Data Fig. S1). After filling the gap with continuous long reads of PN40024.v4, a gap-free PN_T2T genome was finally generated (494.87 Mb), being 69 Mb longer than 12X.v0 (426.18 Mb, Table 1) using the same statistical method. The *k*-mer metric was used to evaluate genomic homozygosity, estimated at 99.8% (Supplementary Data Fig. S2A–D). BUSCO (Benchmarking Universal Single-Copy Orthologs) was used to evaluate genomic completeness; 98.5% of the core conserved plant genes were found complete in the genome assembly (Supplementary Data Fig. S2E), which is 4.8% more than in 12X.v0 (93.7%, Table 1).

**Table 1:** Comparison of genomic features of 12X.v0, 12X.v2, PN40024.v4, and PN_T2T assemblies.

	**12X.v0**	**12X.v2**	**PN40024.v4**	**PN_T2T**
Total sequence length (bp)	426 176 009	458 815 822	462 158 227	494 873 210
Number of chromosomes	19	19	19	19
Contig N50 (bp)	102 700	102 674		25 934 928
Maximum length (bp)	30 274 277	34 568 450	34 942 157	36 684 271
Number of gaps	9429	5106	3391	0
Centromeres annotated				19/ 19
Telomeres annotated				36/38
Bases masked (bp)	303 719 475			328 929 883
Retroelements (bp)	217 819 122			241 027 616
LTR (bp)	212 117 752			235 245 099
Number of genes	28 516	41 182	35 256	37 534
Number of TEs	942 096			935 783
BUSCO (%)	93.70	97.70	98.20	98.50

12X.v0, https://www.ncbi.nlm.nih.gov/data-hub/genome/GCF_000003745.3.nih.gov. 12X.v2, https://urgi.versailles.inra.fr/files/Vini/Vitis%2012X.2%20annotations/12Xv2_grapevine_genome_assembly.fa.zip. PN40024.v4, https://grapedia.org/genomes/https://integrape.eu/resources/genes-genomes/genome-accessions.

**Figure 2:**
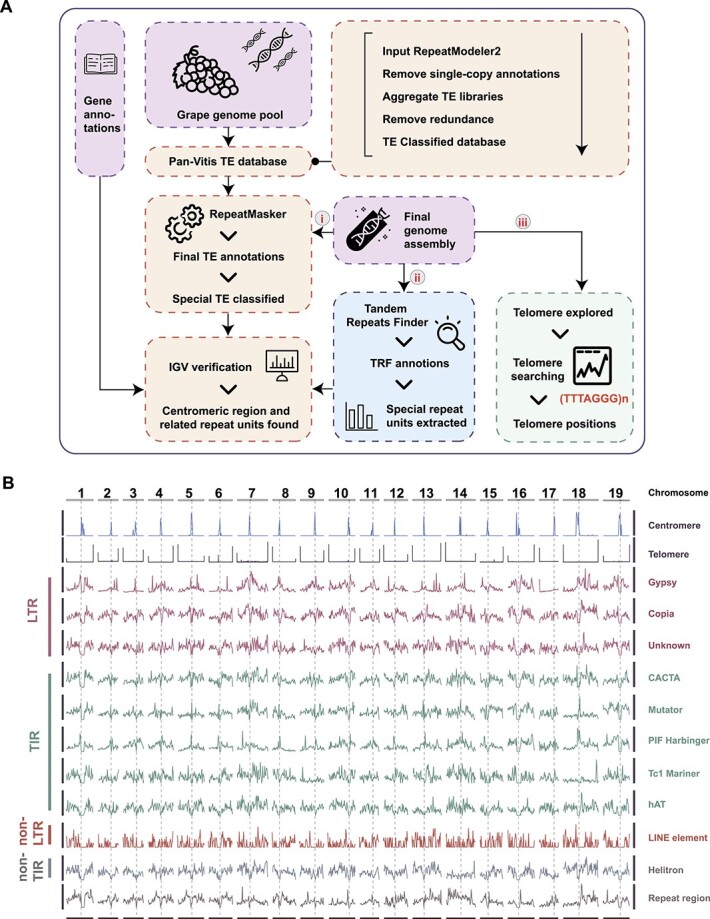
Repeat annotation in PN_T2T reference genome. (**A**) Dataflow of centromere and telomere predictions. (**B**) Chromosomal distribution of telomeres, centromeres, and different types of TE. Dashed vertical lines indicate the center locations of predicted centromeres.

Compared with the 12X.v0 genome, a substantial improvement of several metrics was observed in our PN_T2T assembly. The contig N50 length of PN_T2T was ~250 times higher than that of 12X.v0 (25.93 Mb versus 102 kb), and all 9429 gaps in 12X.v0 and 3391 gaps present in PN40024.v4 were filled in the PN_T2T genome (Table 1, Supplementary Data Table S1, Fig. 1A). As shown in Fig. 1C, 28 gaps in 12X.v0 were filled in PN_T2T, the largest gap being 16 951 bp in the 1-Mb syntenic region on chromosome 18 (Fig. 1C). Orientation errors in 12X.v0 were also corrected, such as inversions and translocations compared with PN_T2T (Fig. 1A, Supplementary Data Fig. S3). For example, two large inversions, which were located surrounding the centromere of chromosome 3 and at the ends of chromosome 5, with the length of 4.9 and 1.9 Mb, were observed between two versions of the assembly, respectively (Fig. 1A and B, Fig. S8). Moreover, 19 centromeres and 36 out of the 38 telomeres were detected on the PN_T2T genome assembly, except one telomere on chromosome 15 and one telomere on chromosome 17. A total of 37 534 genes and 41 064 transcripts were annotated, among which 24 526 (86.01%), 27 696 (78.83%), and 27 717 (78.75%) were shared with older versions PN40024.v2.1 (https://phytozome-next.jgi.doe.gov/info/Vvinifera_v2_1) and PN40024.v4.1 (https://grapedia.org/genomes/), respectively (Supplementary Data Table S2). A total of 5472 (14.58%) genes were not found to correspond in any of the three versions. A total of 97.9% of completely assembled genes was assessed by the BUSCO analysis, and structural domains were detected in 35 508 sequences out of 40 307 unique sequences (88.1%), while PN40024.v4.1 has 38 364 unique sequences, and 29 688 sequences were detected with structural domains (77.4%, Supplementary Data Table S2).

Based on the species-specific pan-TE database constructed by RepeatModeler2, the repeats were detected with the pipeline shown in Fig. 2A. Finally, 66.47% of our gap-free grape genome was marked as repetitive sequences (Fig. 1D). As a comparison, 62.47% of the repetitive sequences were identified in the 12X.v0 genome using the same pipeline (Supplementary Data Table S3). Among the repeats predicted in the PN_T2T genome, the largest portion comprises TEs (63.90%), with a total length of 316 Mb (59.96% and 292 Mb in 12X.v0). The TEs mainly consisted of the long terminal repeat (LTR) type (47.54%), predominantly Gypsy (20.22%) and Copia (19.67%) elements. In total, we detected 276 rDNA sequences, representing 0.019% of the genome.

**Figure 3:**
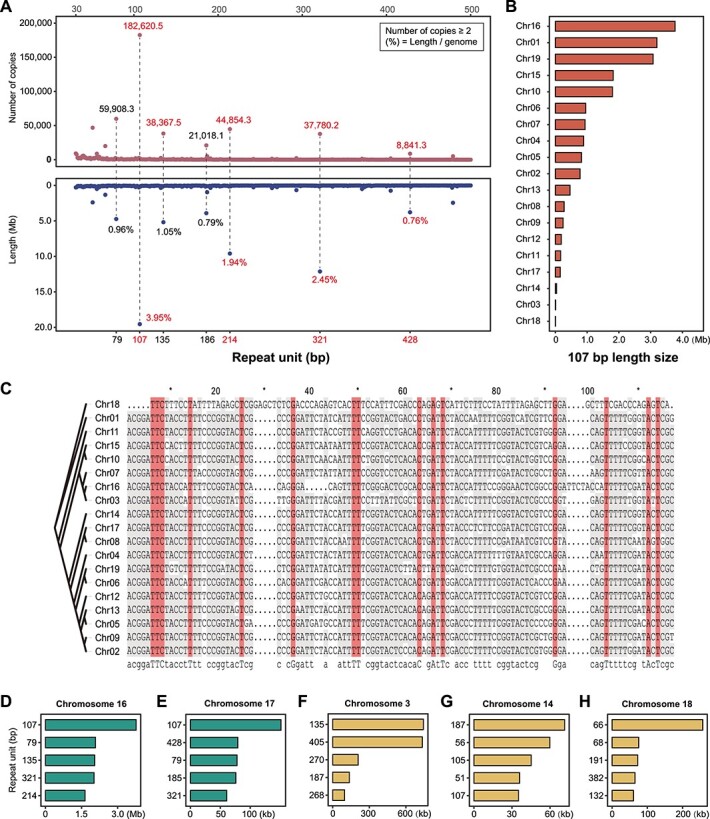
Schematic illustration of centromeric repeat units in the PN_T2T genome. (**A**) Distribution of different repeat unit lengths in the whole genome. The number of different repeat unit copies is indicated in the upper part of the graphs while the chromosomal percentage of different repeat units is shown in the lower part. (**B**) Total length of 107-bp repeat unit copies in each chromosome. (**C**) Alignment of the 107-bp repeat units among 19 chromosomes. (**D**–**H**) Total length of different repeat units in chromosomes 16, 17, 3, 14, and 18, respectively.

### Identification of telomeres and centromeres

To access the telomeric and centromeric regions in PN_T2T, we identified the telomeres and centromeres using the pipeline described in Fig. 2A. For telomeres, we checked the 150-kb sequences at both ends of each chromosome, and the length of the telomere repeat unit was set to range from 5 to 12 bp. Finally, the telomere repeat unit (TTTAGGG/CCCTAAA) was detected, which was the most abundant in the genome and carried by all chromosomes. The same telomere repeat unit was reported in grapes by Melters *et al*. [11] and Castro *et al*. [45]. We further predicted the telomeres in 36 out of 38 telomeres in the PN_T2T genome, except the short arms of chromosome 15 and chromosome 17 (Figs 1A and 2B, Supplementary Data Table S4). Among them, the longest telomere (31 kb) was in the short arm of chromosome 8, with 4479 repeats, while the shortest telomere (1260 bp) was in the long arm of chromosome 7, with only 180 repeats.

To detect centromeric regions, we scanned candidate repeats from 30 to 500 bp along the genome. Tandem Repeats Finder (TRF) found 470 different repeat units in the PN_T2T genome. The 107-bp repeats were the most abundant unit in the whole genome, which had 182 620.5 (copies ≥2) repetitions accounted for ~3.95% of the total genome sequence, followed by 321 bp (2.45%), 214 bp (1.94%), and 135 bp (1.05%) (Fig. 3A). Interestingly, we found the sequences of 214- and 321-bp repeat units consisted of two and three copies of the 107-bp repeat unit, respectively. The TE analyses also supported the centromeric feature of this 107-bp repetitive unit (Fig. 2). Thus, the centromeres were recognized mainly based on 107-bp repeats, and localized on all 19 chromosomes (Figs 1A and 2B, Supplementary Data Table S5). As shown in Fig. 3B, the total length of 107-bp repeats varied from 1.4 kb to 3.8 Mb, but the sequences of the 107-bp repeats were highly conserved among chromosomes (Fig. 3C). The 107-bp repeats were the most abundant in all chromosomes, except chromosomes 3, 14, and 18 (Fig. 3D–H, Supplementary Data Table S6). We found that the 187-bp was the main repeat unit in chromosome 14 and was scattered throughout the whole chromosome, and that 51-, 56-, 105-, and 107-bp repeat units were highly overlapped and enriched in the centromere, which showed a core region in the chromosome through IGV visualization (Supplementary Data Fig. S4). The centromeric repeat unit in chromosome 3 was the 135-bp repeat and its integer multiples (270 and 405 bp). For chromosome 18, 66 bp and its integer multiple 132 bp were the main repeat units (Supplementary Data Fig. S4).

**Figure 4:**
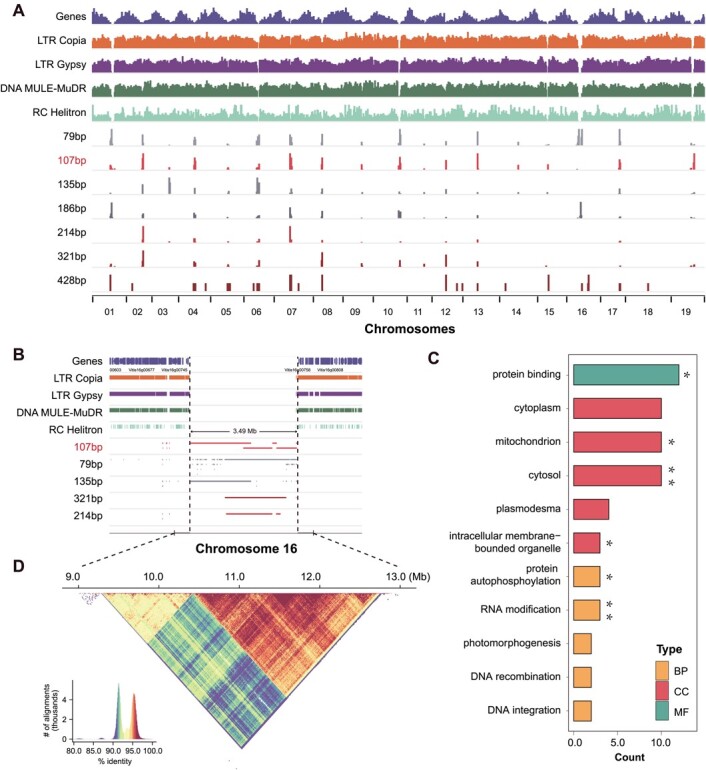
Characteristics and distribution of repeat unit copies in centromeres. (**A**) Distribution of genes, TEs, and different repeat units in the whole genome. (**B**) Visualization of the predicted centromeric region on chromosome 16 in IGV. (C) GO functional annotation of genes captured in centromeres. MF, molecular function; CC, cellular component; BP, biological process. Significant *P*-values for enrichment: ^*^P < .05. ^**^P < .01. (**D**) The triangle shows sequence similarity within each haplotype, colored by identity.

To locate the centromeric repeats, we further examined the relationship between TEs and centromeres. LTR retrotransposons or centromeric retrotransposons (CRs) were usually mixed with tandem repeats and enriched in plant centromeric regions [46, 47]. We found (Fig. 4A) that the genes and TE repeats, such as LTR (Gypsy and Copia), DNA TE (MULE-MuDR), and RC (Helitron), had a low density in the special region when the enormous centromeric tandem repeats enriched in the chromosome were viewed in Integrative Genomics Viewer (IGV) (Fig. 2, Supplementary Data Fig. S4). We then inferred the region with centromeric repeats and low TE density as the centromeres after zooming one by one (Supplementary Data Fig. S4, Supplementary Data Table S5). The pattern of 107 bp was the target, which was highly linked with the centromeric region in grapes. However, there were likely different repeat units and patterns that appeared on chromosomes 3, 14, and 18 (Fig. 3F–H). The scattering of transposons and the distribution of the centromere showed that specific sequence-defined repeat superfamilies were correlated or anticorrelated, to various levels, with centromeric proximity (Figs 2B and 4A), forming density gradients that are the main chromosome-scale repeat-associated features, presumably reflecting overall chromatin structure (Supplementary Data Fig. S4).

To detect the captured genes, we then screened all genes in these regions in the highly linked centromeric region. Interestingly, we found 343 genes (Supplementary Data Tables S7 and S8) captured in the centromeres, which included 179 genes with Uni-Prot ID through BLASTP. Through GO (Gene Ontology) functional annotation, 12 genes were enriched in protein binding (molecular function, MF), such as *VviAMP1* (Uni-Prot ID Q9M1S8), involved in ethylene, gibberellin, and abscisic acid signaling pathways [48, 49]. In addition, we found 10 genes enriched in the cellular component (CC) of the cytosol, mitochondrion and cytoplasm, including auxin transport protein *VviBIG* (Uni-Prot ID Q9SRU2), which influences general growth and development in plants [50]; fumarate hydratase 1 *VviFUM1* (Uni-Prot ID P93033), which catalyzes the active of mitochondrial Krebs cycle-associated enzyme [51]; and 6-phosphogluconate dehydrogenase, decarboxylating 2 *VviPGD2* (Uni-Prot ID Q9FWA3), which plays a key role in the development of the male gametophytes and the interaction between the pollen tube and the ovule [52]. Moreover, RNA modification, protein autophosphorylation, DNA integration, DNA recombination, and photomorphogenesis appeared enriched while exploring biological process (BP) related terms (Fig. 4C).

### Gene clusters in the grapevine reference genome

**Figure 5:**
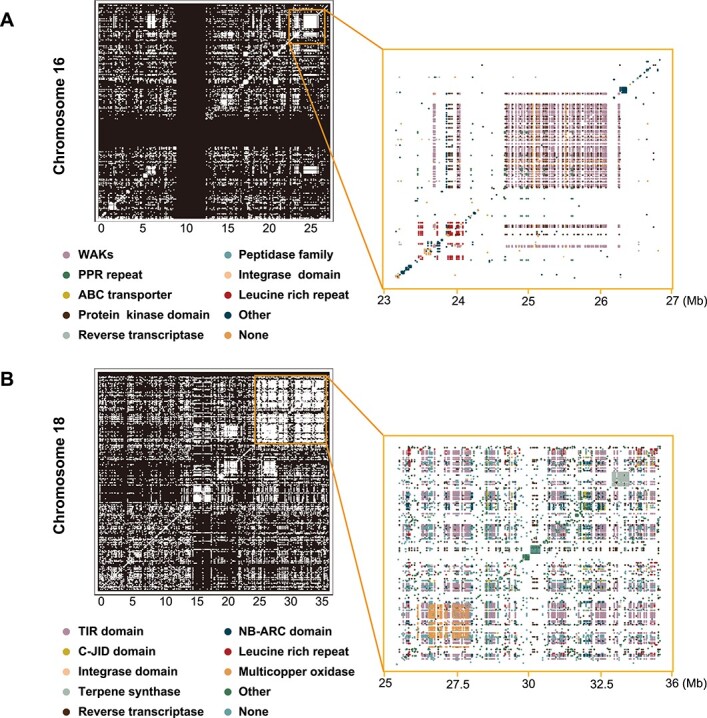
Schematic of identified gene clusters. (**A**) Gene clusters in chromosomes 16 and 16: 22–27 Mb. (**B**) Gene clusters in chromosomes 18 and 18: 25–36 Mb. The graphs on the right represent the regions in orange boxes on the left. Different colors indicate different gene clusters. None, genes for which no domain has been identified by Pfam database; Other, other gene clusters with small numbers of genes.

To infer the gene clusters in the grapevine genome, protein-to-protein alignments among the PN40024 protein-coding genes exposed a rich panoply of duplication structures in terms of genomic positions and functions. Prominent and complex tandem-like blocks of high-similarity genes could be seen via visualizations of all–versus–all alignments (Supplementary Data Fig. S5). We found a total of 377 gene clusters in the grapevine reference genome (Supplementary Data Table S9). These duplications often involved local rearrangements and could extend to megabases with dozens to hundreds of genes involved (Fig. 5). On chromosome 16 (23–27 Mb), there were 599 enriched-domain genes mainly including WAKs (Wall associated receptor kinase galacturonan binding), PPR repeat, Leucine-rich, ABC transporter, Integrase domain, Peptidase family, Protein kinase and Reverse transcriptase (Fig. 5A). On chromosome 18 (25–36 Mb), there were 1 237 genes enriched in domains mainly including Integrase domain, C-JID domain, NB-ARC domain, Leucine rich repeat, Multicopper oxidase, Reverse transcriptase, Terpene synthase, and TIR. Our results show that many of the strongly enriched structural domains are part of the structural domains of plant disease resistance genes (R genes), including NB-ARC, TIR, and structures identified by the Colis database. We analyzed the domain architecture of our 41 064 PN_T2T PCGs and identified 3 381 possible R genes. Collectively, these R genes and gene clusters in grapes highlight a tremendous opportunity for exploring plant defense mechanisms.

### Heterozygous regions remaining after nine generations of selfing

**Figure 6:**
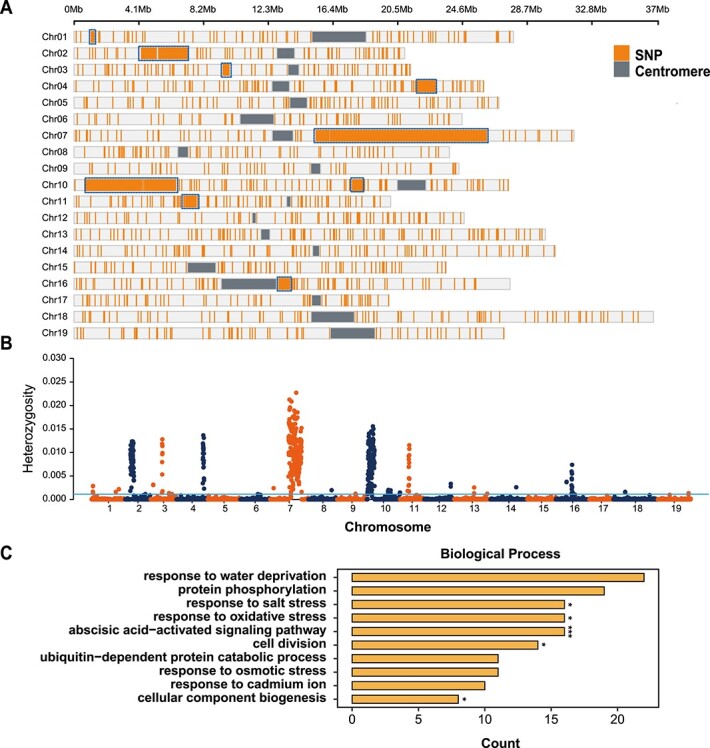
Characterization of heterozygous regions in PN40024. (**A**) Heterozygous sites shared in all four PN40024 samples. The gray bars indicate the centromere region while the orange lines indicate the heterozygous sites that exist in all samples. Blue boxes highlight the large heterozygous fragments. (**B**) Heterozygosity in the PN40024 genome calculated with no overlapping 100-kb windows across four samples. (**C**) GO enrichment analysis of genes contained in heterozygous sites shown in (**A**). Significant *P*-values for enrichment: ^*^P < .05, ^***^P < .001.

Based on the PN_T2T genome assembly, the resequencing data of four PN40024 clones were downloaded from NCBI and analyzed [32, 53]. A total of 244 215 SNPs were detected, among which 208 330 SNPs (85.3%) were shared in all four samples while the other 35 886 SNPs were only present in one to three samples (Fig. 6A). Interestingly, we found nine hotspots of heterozygous SNPs on chromosomes 1, 2, 3, 4, 7, 10, 11, and 16 (Fig. 5A, Supplementary Data Fig. S6). To further investigate the highly heterozygous regions, we examined the top 5% heterozygosity windows and identified a total of nine large continuous fragments (chromosome 1, 1.1–1.3 Mb; chromosome 2, 4.2–7.2 Mb; chromosome 3, 9.4–9.9 Mb; chromosome 4, 21.8–22.9 Mb; chromosome 7, 15.3–26.2 Mb; chromosome 10, 0.7–6.5 Mb, 17.6–18.3 Mb; chromosome 11, 7.1–7.8 Mb; chromosome 16, 13.0–13.5 Mb). The GO enrichment analysis of the genes in these regions showed that the most significantly enriched terms were response to water deprivation, protein phosphorylation, cell division, response to oxidative stress, and response to salt stress, which were closely associated with key physiological activities in plants (Supplementary Data Tables S10 and S11, Fig. 6C, Supplementary Data Fig. S7). We further phased these nine hotspots of heterozygous regions on the PN_T2T reference genome (Supplementary Data File 2).

## Discussion

A complete reference genome is essential for crop genetic studies and breeding purposes. The latest version of the PN40024.v4 assembly improved the reference resource by including long-read sequences and by gathering a gold-standard annotation [31]. Nevertheless, these previous versions still possessed thousands of gaps and lacked repetitive regions, centromeres, and telomeres, all of which limited access to variants within these regions. On occasion such unreachable regions underlie quantitative trait loci (QTLs) for important agronomic traits, such as berry color and sex determination on chromosome 2 [30, 54–56] and disease resistance on chromosome 14 [57, 58]. A full reference genome has therefore great potential to reveal the missing heritabilities of important polygenic agronomic traits, increasing genetic gain in grapevine breeding.

More and more investigations suggest the important functions of gene clusters, with a total of 377 gene clusters being detected in PN_T2T. The grapevine genome is also widely used in studies of plant evolution and comparative genomics because of its important phylogenetic position in the evolution of eudicots [32]. The T2T version could be widely used in plant evolutionary genomics, especially the repetitive sequences, centromeres, and telomeres. The T2T gap-free reference genome has incorporated gene annotations of previous versions with more accurate TE annotation (up to ~67% of the genome), which will be an important resource for grapevine functional genomics and breeding.

### Architecture and context of plant centromeres

The centromeric region ranges from kilobases to gigabases in length, including >90% tandem repeats [59]. The centromere is among the last great unknowns in genomics, since it was inaccessible by previous sequencing technologies. Assemblies often collapse due to the highly repetitive nature of the centromeric region. We assembled and annotated centromeres for all 19 chromosomes of the grapevine genome (Fig. 1). Most of the chromosomes have a single centromere while others could have multiple centromeric regions—the so-called holocentromere [60, 61]. On chromosomes 16 and 18 we found tandem repeats in many regions, while on other chromosomes only a single peak was detected (Fig. 2B), suggesting that the structure of the centromeric region might be more complicated and requires further investigation.

In the PN40024 grapevine reference genome there are three major repetitive patterns across the 19 chromosomes, suggesting different chromosomal evolutionary histories (Fig. 3D–H). On chromosomes 3, 14, and 18, we found 135-, 56-, and 66-bp tandem repeats, respectively (Supplementary Data Fig. S4), while on other chromosomes the major unit of tandem repeats was 107 bp (Figs 3D–H and 4B and D). The evolutionary histories of the centromeres of each grapevine chromosome are still an open question to be addressed with all *Vitis* genomes. Previous comparative genomic analyses suggested that the centromere is conservative among closely related species with a constant number of chromosomes [9]. Transformation of centromeric structures occurs during chromosome division and fusion when the number of chromosomes changes throughout evolution. The muscadine grape (*Vitis rotundifolia*) has 20 chromosomes, with chromosomes 7 and 13 collinear with subgenus *Vitis* chromosome 7, which is associated with a chromosome fusion event [62]. Only one centromeric region is left on chromosome 7 in our grapevine reference genome (Fig. 2B, Supplementary Data Fig. S4), suggesting one centromere was lost during the evolution of the genus *Vitis*.

Centromeric architecture shaped the content within the genome, population genetic diversity within species, and genetic differentiation among species. Population genetic analyses have previously revealed that the genetic variants in the centromeric region are highly linked, with much lower genetic diversity compared with chromosome arms [63]. The centromeres capture tens to thousands of genes that are highly linked to the centromeric tandem repeats. These genes, along with the centromeric region, are functional as supergenes [64]. In total, we found 343 captured genes (Supplementary Data Table S7) in the centromeric region in the grapevine reference genome. Interestingly, the genes are mainly involved in the ethylene, gibberellin, and abscisic acid signaling pathways [48, 49].

### Hotspots of heterozygosity in a nearly homozygous genotype

The current plant used to build the grapevine reference genome originated from the ‘Helfensteiner’ cultivar selfed for nine generations, which resulted in a 99.8% homozygous genome (Supplementary Data Fig. S2A–D). The remaining heterozygous sites are still of interest as they could represent hotspots of required heterozygosity, with lethal consequences if found in the homozygous state. Thus, we collected Illumina resequencing reads for four clones of PN40024 maintained in different international laboratories. Interestingly, the heterozygous SNPs and structure variants (SVs) were enriched in specific regions when mapped to PN_T2T. In total, we found 208 330 heterozygous SNPs shared by the four samples, and 35 886 SNPs specific to one to three samples. The former is more likely the original variant of PN40024 after nine generations of selfing while the latter could be somatic variants generated during distribution and tissue culture in the different laboratories. Interestingly, we found that hotspots of common variants were enriched in central biological processes, including the oxidation–reduction process and protein phosphorylation. The hotspots on chromosome 2 also covered the sex-determination QTL region (Fig. 6), which complicated the mining of the sex-determination genes [30, 56], because the candidate genes were not present in the old version of the reference genome. It has been reported that, during the clonal reproduction of fruit trees, such heterozygous deleterious variants accumulate in the genome [30, 65]. The clonal processes hide recessive deleterious variants, including small SNPs and indels and large structural variants, in a heterozygous state [30, 55]. Strong inbreeding depression has been commonly observed in clonal crops, including potato, cassava, citrus, and grapevine [55, 66–68], since the strongly deleterious variants in these genomic regions have been exposed to lethal or strong recessive selection during selfing cycles. In grapevine breeding, inbreeding and outcrossing depression were commonly detected because the hidden heterozygous recessive deleterious variants that increased during clonal propagation were exposed during sexual reproduction.

Altogether, and still acknowledging all previous sequencing efforts, our work represents the completion of a full T2T sequence of the grape reference genome. This assembly, together with the previous manually curated annotation, currently being transferred into PN_T2T, should represent the gold standard for the grapevine community. In line with this forecast, the T2T assembly and its updated annotation are available for download at the Grape Genomics Encyclopedia (GRAPEDIA; https://grapedia.org/), where it will be used along with different application program interfaces, including gene cards, transcriptomic data visualizations, and software for variation–gene expression–phenotype associations.

## Materials and methods

### Sample collection and genome sequencing

PN40024 is a line that belongs to one of the near-homozygous lines originally derived from the ‘Helfensteiner’ cultivar [31] by successive selfing steps, estimated to be close to 97% homozygosity as tested by SSR markers [32]. We got this inbred material from INRAE under a Material Transfer Agreement (MTA) and transplanted it in the greenhouse belonging to AGIS (Agricultural Genomics Institute at Shenzhen, Chinese Academy of Agricultural Sciences, Shenzhen, China) for subsequent experiments. Young leaves and ovules from PN40024 were flash-frozen in liquid nitrogen. Genomic DNA and RNA were isolated using the DNeasy Plant Mini Kit (Qiagen) following the manufacturer’s instructions. For PacBio HiFi sequencing, two single-molecule real-time cells were sequenced on a PacBio Sequel II platform, and a total of 21 Gb of HiFi reads was generated using CCS (https://github.com/PacificBiosciences/ccs) with the default parameter for the sequenced accessions. For RNA-seq, 10 μg of poly(A) mRNA that isolated from total RNA was used for preparing Illumina RNA-seq libraries for each sample. These libraries were then sequenced using the Illumina HiSeq™ 2000 system in accordance with the manufacturer’s instructions.

### Telomere-to-telomere genome assembly

Initially, the PN40024 genome was assembled by incorporating PacBio single-molecule real-time long-read sequences. Reads generated by the PacBio Sequel II platform were self-corrected, trimmed, and assembled by hifiasm, using default parameters (https://github.com/chhylp123/hifiasm) [43]. The initial output of hifiasm (v.0.13) yielded the p_ctg draft assembly. Genome heterozygosity was estimated using a *k*-mer-based approach by GenomeScope 2.0 [69]; it was estimated to be close to 99.8% homozygosity (Supplementary Data Fig. S2A–D). Then, homology-based scaffolds were generated with MUMmer (v.4.0.0) [70] ‘scaffold’, using the 12X.v0 reference genome (Supplementary Data Fig. S3). By applying MUMmer tools, we ordered and oriented the contig-level assemblies into 19 chromosomes, and joined the adjacent contigs to generate a scaffold with 100 N. Finally, we adjusted the assembly manually through aligning the genome sequencing data from the previous version of PN40024, which was mapped to the genome assembly by minimap2 (v.2.21) and visualized in IGV (v.2.12.3) software to observe whether the gap regions were supported by reads (Supplementary Data Fig. S1). Filling and closing of the gaps with the selected and assigned contigs were performed by mapping the 50-bp sequences around the gap to continuous long reads of PN40024.v4 and obtaining the gapless T2T PN40024 assembly for all 19 grape chromosomes. The assembly was inspected based on BUSCO [71] completeness and the duplication score. For the phasing of highly heterozygous regions, minimap2 was used to align all reads to the PN_T2T assembly. The primary contigs assembled by hifiasm and ragtag were used to phase these contigs into two haplotypes.

### Annotation of genes and transposable elements

We have used a self-developed method for genome annotation. The putative genes were first searched for by using transcripts and Uni-Prot as evidence. A preliminary gene model was then built for the putative genes and further search was performed using AUGUSTUS (v.3.4.0) [72]. All the found putative genes fragments were then filtered, including genes involving duplicated regions, genes with coding sequence lengths shorter than 90 and genes not supported by any evidence. We attempted to complement missing genes and the complete genes were subjected to alternative splicing analyses. Finally, all the results were examined by hidden Markov models downloaded from the Pfam database to obtain the final gene models. Interproscan (v.5.56–89.0) [73] was used for function annotation for our assembly, and Pfam (v.34.0) [74] and Coils (v.2.2.1) [75] were used for the identification of structural domains (https://github.com/unavailable-2374/Genome-Wide-Annotation-Pipeline).

The primary repeat analysis is outlined in Fig. 2A and began with the construction of a pan-*Vitis* database of repeat families by RepeatModeler (open-2.0.3) [76] and a series of scripts, which was then applied with RepeatMasker (open-4.1.2). For building this pan-*Vitis* repeat database we downloaded 17 *Vitis* genomes from NCBI, then used RepeatModeler2 to identify TE families. After that, we got 17 consensus fasta files of TE families and by removing the single-copy and failed annotations we aggregated these files. We used NCBI-BLAST+2.9.0 [77] to remove some redundant sequences (−i 80%, −l 80%). Next, we got the final file of repeat identity, then used deepTE [78] with the Plant model to classify the unclassified repeat elements. Finally, the repetitive sequence of the complete reference genome was annotated by RepeatMasker.

### Genome comparison between different versions of the grapevine reference genome

To compare previous versions of the grapevine genome with PN_T2T, we aligned the genomes using minimap2 and indexed the alignment BAM file using SAMtools (minimap2 -ax asm5 -t 4 --eqx A.fa B.fa | samtools sort -O BAM - > A_B.bam, samtools index A_B.bam). Next, to detect structural variations between genomes, we needed to find synteny and structural rearrangements between the genomes. For this, we used SyRI (syri -c A_B.bam -r A.fa -q B.fa -F B --prefix A_B). Finally, Plotsr was used to generate the graph (plotsr --sr A_Bsyri.out --sr B_Csyri.out --sr C_Dsyri.out --genomes genomes.txt -o output_plot.pdf, https://github.com/schneebergerlab/plotsr). MUMmer (v.4.0.0) was used to compare the 12X.v0 genome with the reference genome PN_T2T using whole-genome alignments [70]. First, we aligned the two genome sequences using nucmer (nucmer --mum) and then filtered one-to-one alignments with a minimum alignment length of 10 000 bp (delta-filter -i 95 -l 10 000).

SAMtools (v.1.7) was used to extract the sequence of chromosome 18 (25.0–26.0 Mb) in 12X.v0 and align the sequence in PN_T2T. The gap information was detected with a python script (getgaps.py) and finally we used LINKVIEW2 (https://github.com/YangJianshun/LINKVIEW2) to visualize the alignment results.

### Identification of telomeres and centromeres

The telomere repeat units were explored by using the TIDK (v.0.2.0) (https://github.com/tolkit/telomeric-identifier) with options tidk explore -f genome.fa --minimum 5 --maximum 12 -o tidk_explore -t 2 --log --dir telomere_find --extension TSV. Then the whole genome was searched using the following parameters: tidk search -f genome.fa -s TTTAGGG -o tidk_search --dir telomere_find. Finally, we completed the rapid statistics of telomeres based on the TIDK plot and used the R script to visualize the telomere peak.

For centromere annotation, TRF (v.4.09) [79] was used to finish tandem repeat annotation with the parameters trf genome.fa 2 7 7 80 10 50 500 -f -d -m, and then we merged the results of annotation by using TRF2GFF (https://github.com/Adamtaranto/TRF2GFF). To complete the data statistics and visualization, we used information extracted by using the awk command in the Linux system and analyzed the results in IGV (v.2.12.3) [80]. We used four softwares to show more details about the centromeric region: Iqtree (v. 2.1.4-beta) [81] was used to achieve the phylogenetic tree (options: -m GTR + I + G -bb 1000 -bnni -alrt 1000); itol (v.6) [82] was used to visualize the phylogenetic tree; GeneDoc (v.2.7.0) (https://github.com/karlnicholas/genedoc) was used to achieve multiple sequence alignment; and R script was used to plot the data statistics and typeset details.

To detect the functions of the genes captured in the centromeric regions, we downloaded the protein sequence library of Swiss-Prot (2022/08/30, https://ftp.ncbi.nlm.nih.gov/blast/db/FASTA/) for a local blast. After this, we extracted all the protein sequences of PN_T2T blasted by diamond (v.2.0.15) (parameter: -k 1 -e 0.00001, https://github.com/python-diamond/Diamond). We further uploaded the Swiss-Prot ID to DAVID (https://david.ncifcrf.gov/tools.jsp) and completed GO enrichment and annotation. Finally, data visualization was completed by our R scripts.

### Identification of gene clusters

To define the clustered genes in the reference genome, protein sequences were extracted using gffread and then filtered by e-value <1e-5 and similarity >30% using BLASTP for all-versus-all alignments. The filtered alignment results were combined with functional annotations to filter out alignment results that did not share the same structural domains. Finally, we determined the presence of gene clusters by identifying three consecutive identical Pfam accessions (https://www.ebi.ac.uk/interpro/entry/pfam/#table), using such Pfam accessions as seeds, and going up and down 30 genes to find genes with the same Pfam accessions. In total, 377 gene clusters were found (Supplementary Data Table S8).

### Heterozygosity in PN40024 clones

Four resequencing samples were downloaded from the NCBI database (SRR6156373, SRR8835144, SRR8835157, SRR8835168) and mapped to the newly assembled PN_T2T genome for SNP calling. Quality-controlled reads were mapped to the genome using bwa (v.0.7.15) with the default parameters. SAMtools (v.1.4) and GATK (v.4.1.8) were used for sorting and indexing the bam file with no duplicates. The gvcf files were combined in GATK and were used to join calling SNPs across all samples. To obtain high-quality SNPs, we performed strict filtering of the SNP calls based on the following criteria: (i) SNPs with more than two alleles were removed in all samples in vcftools with parameters --min-alleles 2 --max-alleles 2; (ii) we removed the SNPs with quality scores (GQ) <30 (--minGQ 30) and missing rate 0 (--max-missing 1); (iii) SNPs with minor allele frequencies (MAFs) ≥.01 to remove the invariable sites.

## Acknowledgements

This work was supported by the National Natural Science Fund for Excellent Young Scientists Fund Program (Overseas) to Y.Z., the National Key Research and Development Program of China (grant 2019YFA0906200), the Agricultural Science and Technology Innovation Program (CAAS-ZDRW202101), the Shenzhen Science and Technology Program (grant KQTD2016113010482651), and the BMBF-funded de.NBI Cloud within the German Network for Bioinformatics Infrastructure (de.NBI). We thank Bianca Frommer, Marie Lahaye, David Navarro-Payá, Marcela K. Tello-Ruiz, and Kapeel Chougule for their help in analyzing the RNA-seq data and in running the gene annotation pipeline. This study is also based upon work from COST Action CA17111 INTEGRAPE and from the COST Innovators Grant GRAPEDIA (IG17111), supported by COST (European Cooperation in Science and Technology). JTM is supported by PID2021-128865NB-I00 and RYC-2017-23 645 grants from AEI (Spain).

## Author contributions

Y.Z. conceived and designed the project with H.X., Z.C., and C.R. The PN40024 sample was provided by Z.C. under an MTA signed with INRAE. X.S., W.L., X.X., and Z.M. performed the tissue culture of the sample in the greenhouse. X.S., X.W., H.X., N.W., F.Z., H.X., H.Z. and Y.W. performed the bioinformatic analyses. A.V., K.A., D.H., J.G., J.T.M., D.W., Z.L., X.L., and W.L. performed the gene annotation. Y.P., S.H., Z.L., W.L., X.W., Y.F., Y.W., H.W. and C.L. assisted in bioinformatics analyses. X.S., S.C., X.W., H.X., and Y.Z. wrote the manuscript with comments and input from all authors.

## Data availability

All PacBio sequence data have been deposited in the NCBI Sequence Read Archive under project number PRJNA882193 and the National Genomics Data Center (NGDC) Genome Sequence Archive (GSA) (https://ngdc.cncb.ac.cn/gsa/), with BioProject number PRJCA012093. The assembly and annotation as well as the sequences of centromeres and heterozygous regions have been deposited in zenodo: https://zenodo.org/record/7751391#.ZBgVmcJBy3A. The assembly and its annotation will be also hosted in the GRAPEDIA portal (https://grapedia.org/).

## Code availability

All the scripts and pipelines used in this study have been archived in GitHub: https://github.com/zhouyflab.

## Conflict of interest statement

The authors declare no conflict of interest.

## Supplementary data

Supplementary data is available at *Horticulture Research* online.

## Supplementary Material

Web_Material_uhad061

## References

[1] Lander ES , Linton LM, Birren B et al. Initial sequencing and analysis of the human genome. Nature. 2001;409:860–921. 11237011 10.1038/35057062

[2] Venter JC , Adams MD, Myers EW et al. The sequence of the human genome. Science. 2001;291:1304–51. 11181995 10.1126/science.1058040

[3] Rice ES , Green RE. New approaches for genome assembly and scaffolding. Annu Rev Anim Biosci. 2019;7:17–40. 30485757 10.1146/annurev-animal-020518-115344

[4] Giani AM , Gallo GR, Gianfranceschi L et al. Long walk to genomics: history and current approaches to genome sequencing and assembly. Comput Struct Biotechnol J. 2020;18:9–19. 31890139 10.1016/j.csbj.2019.11.002 PMC6926122

[5] Nurk S , Koren S, Rhie A et al. The complete sequence of a human genome. Science. 2022;376:44–53. 35357919 10.1126/science.abj6987 PMC9186530

[6] Talbert PB , Henikoff S. What makes a centromere? Exp Cell Res. 2020;389:111895. 32035948 10.1016/j.yexcr.2020.111895

[7] Naish M , Alonge M, Wlodzimierz P et al. The genetic and epigenetic landscape of the *Arabidopsis* centromeres. Science. 2021;374:eabi7489. 34762468 10.1126/science.abi7489 PMC10164409

[8] Sundararajan K , Straight AF. Centromere identity and the regulation of chromosome segregation. Front Cell Dev Biol. 2022;10:914249. 35721504 10.3389/fcell.2022.914249 PMC9203049

[9] Liao Y , Zhang X, Li B et al. Comparison of *Oryza sativa* and *Oryza brachyantha* genomes reveals selection-driven gene escape from the centromeric regions. Plant Cell. 2018;30:1729–44. 29967288 10.1105/tpc.18.00163 PMC6139686

[10] Rudd MK , Wray GA, Willard HF. The evolutionary dynamics of alpha-satellite. Genome Res. 2006;16:88–96. 16344556 10.1101/gr.3810906 PMC1356132

[11] Melters DP , Bradnam KR, Young HA et al. Comparative analysis of tandem repeats from hundreds of species reveals unique insights into centromere evolution. Genome Biol. 2013;14:R10. 23363705 10.1186/gb-2013-14-1-r10 PMC4053949

[12] Fajkus J , Sýkorová E, Leitch AR. Telomeres in evolution and evolution of telomeres. Chromosome Res. 2005;13:469–79. 16132812 10.1007/s10577-005-0997-2

[13] Podlevsky JD , Chen JJ. Evolutionary perspectives of telomerase RNA structure and function. RNA Biol. 2016;13:720–32. 27359343 10.1080/15476286.2016.1205768 PMC4993307

[14] Turner KJ , Vasu V, Griffin DK. Telomere biology and human phenotype. Cell. 2019;8:73. 10.3390/cells8010073 PMC6356320 30669451

[15] Coulon S , Vaurs M. Telomeric transcription and telomere rearrangements in quiescent cells. J Mol Biol. 2020;432:4220–31. 32061930 10.1016/j.jmb.2020.01.034

[16] Yuan X , Dai M, Xu D. Telomere-related markers for cancer. Curr Top Med Chem. 2020;20:410–32. 31903880 10.2174/1568026620666200106145340 PMC7475940

[17] Engin AB , Engin A. The connection between cell fate and telomere. Adv Exp Med Biol. 2021;1275:71–100. 33539012 10.1007/978-3-030-49844-3_3

[18] Kobayashi T . How does genome instability affect lifespan?: roles of rDNA and telomeres. Genes Cells. 2011;16:617–24. 21605287 10.1111/j.1365-2443.2011.01519.x PMC3178783

[19] Xu Y , Wu Y, Wang L et al. Identification of curcumin as a novel natural inhibitor of rDNA transcription. Cell Cycle. 2020;19:3362–74. 33171062 10.1080/15384101.2020.1843817 PMC7751654

[20] Sasaki M , Kobayashi T. Gel electrophoresis analysis of rDNA instability in *Saccharomyces cerevisiae*. Methods Mol Biol. 2021;2153:403–25. 32840795 10.1007/978-1-0716-0644-5_28

[21] Kille B , Balaji A, Sedlazeck FJ et al. Multiple genome alignment in the telomere-to-telomere assembly era. Genome Biol. 2022;23:182. 36038949 10.1186/s13059-022-02735-6 PMC9421119

[22] Logsdon GA , Vollger MR, Eichler EE. Long-read human genome sequencing and its applications. Nat Rev Genet. 2020;21:597–614. 32504078 10.1038/s41576-020-0236-x PMC7877196

[23] Miga KH , Sullivan BA. Expanding studies of chromosome structure and function in the era of T2T genomics. Hum Mol Genet. 2021;30:R198–205. 34302168 10.1093/hmg/ddab214 PMC8631062

[24] Wang B , Yang X, Jia Y et al. High-quality *Arabidopsis thaliana* genome assembly with Nanopore and HiFi long reads. Genomics Proteomics Bioinformatics. 2022;20:4–13. 34487862 10.1016/j.gpb.2021.08.003 PMC9510872

[25] Belser C , Baurens FC, Noel B et al. Telomere-to-telomere gapless chromosomes of banana using nanopore sequencing. Commun Biol. 2021;4:1047. 34493830 10.1038/s42003-021-02559-3 PMC8423783

[26] Deng Y , Liu S, Zhang Y et al. A telomere-to-telomere gap-free reference genome of watermelon and its mutation library provide important resources for gene discovery and breeding. Mol Plant. 2022;15:1268–84. 35746868 10.1016/j.molp.2022.06.010

[27] Zhang Y , Fu J, Wang K et al. The telomere-to-telomere gap-free genome of four rice parents reveals SV and PAV patterns in hybrid rice breeding. Plant Biotechnol J. 2022;20:1642–4. 35748695 10.1111/pbi.13880 PMC9398309

[28] Yue J , Chen Q, Wang Y et al. Telomere-to-telomere and gap-free reference genome assembly of the kiwifruit *Actinidia chinensis*. Hortic Res. 2023;10:uhac264. 36778189 10.1093/hr/uhac264 PMC9909506

[29] Grassi F , De Lorenzis G. Back to the origins: background and perspectives of grapevine domestication. Int J Mol Sci. 2021;22:4518. 10.3390/ijms22094518 PMC8123694 33926017

[30] Zhou Y , Minio A, Massonnet M et al. The population genetics of structural variants in grapevine domestication. Nat Plants. 2019;5:965–79. 31506640 10.1038/s41477-019-0507-8

[31] Velt A , Frommer B, Blanc S et al. An improved reference of the grapevine genome reasserts the origin of the PN40024 highly-homozygous genotype. G3 (Bethesda). 2023;**13**. 10.1093/g3journal/jkad067 PMC10151409 36966465

[32] Jaillon O , Aury JM, Noel B et al. The grapevine genome sequence suggests ancestral hexaploidization in major angiosperm phyla. Nature. 2007;449:463–7. 17721507 10.1038/nature06148

[33] Canaguier A , Grimplet J, di Gaspero G et al. A new version of the grapevine reference genome assembly (12X.v2) and of its annotation (VCost.v3). Genom Data. 2017;14:56–62. 28971018 10.1016/j.gdata.2017.09.002 PMC5612791

[34] Navarro-Payá D , Santiago A, Orduña L et al. The grape gene reference catalogue as a standard resource for gene selection and genetic improvement. Front Plant Sci. 2021;12:803977. 35111182 10.3389/fpls.2021.803977 PMC8801485

[35] Massonnet M , Cochetel N, Minio A et al. The genetic basis of sex determination in grapes. Nat Commun. 2020;11:2902. 32518223 10.1038/s41467-020-16700-z PMC7283251

[36] Vondras AM , Lerno L, Massonnet M et al. Rootstock influences the effect of grapevine leafroll-associated viruses on berry development and metabolism via abscisic acid signalling. Mol Plant Pathol. 2021;22:984–1005. 34075700 10.1111/mpp.13077 PMC8295520

[37] Minio A , Cochetel N, Vondras AM et al. Assembly of complete diploid-phased chromosomes from draft genome sequences. G3 (Bethesda). 2022;12:jkac143. 10.1093/g3journal/jkac143 PMC9339290 35686922

[38] Chin C-S , Peluso P, Sedlazeck FJ et al. Phased diploid genome assembly with single-molecule real-time sequencing. Nat Methods. 2016;13:1050–4. 27749838 10.1038/nmeth.4035 PMC5503144

[39] Minio A , Massonnet M, Figueroa-Balderas R et al. Diploid genome assembly of the wine grape Carménère. G3 (Bethesda). 2019;9:1331–7. 30923135 10.1534/g3.119.400030 PMC6505170

[40] Minio A , Massonnet M, Figueroa-Balderas R et al. Iso-Seq allows genome-independent transcriptome profiling of grape berry development. G3 (Bethesda). 2019;9:755–67. 30642874 10.1534/g3.118.201008 PMC6404599

[41] Roach MJ , Johnson DL, Bohlmann J et al. Population sequencing reveals clonal diversity and ancestral inbreeding in the grapevine cultivar Chardonnay. PLoS Genet. 2018;14:e1007807. 30458008 10.1371/journal.pgen.1007807 PMC6279053

[42] Maestri S , Gambino G, Lopatriello G et al. 'Nebbiolo' genome assembly allows surveying the occurrence and functional implications of genomic structural variations in grapevines (*Vitis vinifera* L.). BMC Genomics. 2022;23:159. 35209840 10.1186/s12864-022-08389-9 PMC8867635

[43] Cheng H , Concepcion GT, Feng X et al. Haplotype-resolved de novo assembly using phased assembly graphs with hifiasm. Nat Methods. 2021;18:170–5. 33526886 10.1038/s41592-020-01056-5 PMC7961889

[44] Mascher M , Wicker T, Jenkins J et al. Long-read sequence assembly: a technical evaluation in barley. Plant Cell. 2021;33:1888–906. 33710295 10.1093/plcell/koab077 PMC8290290

[45] Castro C , Carvalho A, Gaivão I et al. Evaluation of copper-induced DNA damage in *Vitis vinifera* L. using Comet-FISH. Environ Sci Pollut Res Int. 2021;28:6600–10. 33006094 10.1007/s11356-020-10995-7

[46] Guo X , Su H, Shi Q et al. De novo centromere formation and centromeric sequence expansion in wheat and its wide hybrids. PLoS Genet. 2016;12:e1005997. 27110907 10.1371/journal.pgen.1005997 PMC4844185

[47] Fernandes JB , Wlodzimierz P, Henderson IR. Meiotic recombination within plant centromeres. Curr Opin Plant Biol. 2019;48:26–35. 30954771 10.1016/j.pbi.2019.02.008

[48] Saibo NJ , Vriezen WH, de Grauwe L et al. A comparative analysis of the *Arabidopsis* mutant amp1-1 and a novel weak amp1 allele reveals new functions of the AMP1 protein. Planta. 2007;225:831–42. 17006669 10.1007/s00425-006-0395-9

[49] Shi H , Ye T, Wang Y et al. *Arabidopsis* ALTERED MERISTEM PROGRAM 1 negatively modulates plant responses to abscisic acid and dehydration stress. Plant Physiol Biochem. 2013;67:209–16. 23603279 10.1016/j.plaphy.2013.03.016

[50] Gil P , Dewey E, Friml J et al. BIG: a calossin-like protein required for polar auxin transport in *Arabidopsis*. Genes Dev. 2001;15:1985–97. 11485992 10.1101/gad.905201 PMC312751

[51] Zubimendi JP , Martinatto A, Valacco MP et al. The complex allosteric and redox regulation of the fumarate hydratase and malate dehydratase reactions of *Arabidopsis thaliana* Fumarase 1 and 2 gives clues for understanding the massive accumulation of fumarate. FEBS J. 2018;285:2205–24. 29688630 10.1111/febs.14483

[52] Hölscher C , Lutterbey MC, Lansing H et al. Defects in peroxisomal 6-phosphogluconate dehydrogenase isoform PGD2 prevent gametophytic interaction in *Arabidopsis thaliana*. Plant Physiol. 2016;171:192–205. 26941195 10.1104/pp.15.01301 PMC4854672

[53] Magris G , di Gaspero G, Marroni F et al. Genetic, epigenetic and genomic effects on variation of gene expression among grape varieties. Plant J. 2019;99:895–909. 31034726 10.1111/tpj.14370

[54] Fournier-Level A , le Cunff L, Gomez C et al. Quantitative genetic bases of anthocyanin variation in grape (*Vitis vinifera* L. ssp. *sativa*) berry: a quantitative trait locus to quantitative trait nucleotide integrated study. Genetics. 2009;183:1127–39. 19720862 10.1534/genetics.109.103929 PMC2778965

[55] Zhou Y , Massonnet M, Sanjak JS et al. Evolutionary genomics of grape (*Vitis vinifera* ssp. *vinifera*) domestication. Proc Natl Acad Sci USA. 2017;114:11715–20. 29042518 10.1073/pnas.1709257114 PMC5676911

[56] Zou C , Massonnet M, Minio A et al. Multiple independent recombinations led to hermaphroditism in grapevine. Proc Natl Acad Sci USA. 2021;118:2023548118. 10.1073/pnas.2023548118 PMC8053984 33837155

[57] Riaz S , Tenscher AC, Rubin J et al. Fine-scale genetic mapping of two Pierce's disease resistance loci and a major segregation distortion region on chromosome 14 of grape. Theor Appl Genet. 2008;117:671–81. 18516585 10.1007/s00122-008-0802-7

[58] Morales-Cruz A , Aguirre-Liguori J, Massonnet M et al. Multigenic resistance to *Xylella fastidiosa* in wild grapes (*Vitis* sps.) and its implications within a changing climate. bioRxiv. 2022.10.08.511428. 10.1038/s42003-023-04938-4 PMC10229667 37253933

[59] McKinley KL , Cheeseman IM. The molecular basis for centromere identity and function. Nat Rev Mol Cell Biol. 2016;17:16–29. 26601620 10.1038/nrm.2015.5 PMC8603311

[60] Steiner FA , Henikoff S. Holocentromeres are dispersed point centromeres localized at transcription factor hotspots. eLife. 2014;3:e02025. 24714495 10.7554/eLife.02025 PMC3975580

[61] Hofstatter PG , Thangavel G, Lux T et al. Repeat-based holocentromeres influence genome architecture and karyotype evolution. Cell. 2022;185:3153–3168.e18. 35926507 10.1016/j.cell.2022.06.045

[62] Cochetel N , Minio A, Massonnet M et al. Diploid chromosome-scale assembly of the *Muscadinia rotundifolia* genome supports chromosome fusion and disease resistance gene expansion during *Vitis* and *Muscadinia* divergence. G3 (Bethesda). 2021;11:jkab033. 10.1093/g3journal/jkab033 PMC8049426 33824960

[63] Kawabe A , Forrest A, Wright SI et al. High DNA sequence diversity in pericentromeric genes of the plant *Arabidopsis lyrata*. Genetics. 2008;179:985–95. 18505875 10.1534/genetics.107.085282 PMC2429891

[64] Thompson MJ , Jiggins CD. Supergenes and their role in evolution. Heredity. 2014;113:1–8. 24642887 10.1038/hdy.2014.20 PMC4815649

[65] Xiao H , Zhongjie L, Wang N et al. Adaptive and maladaptive introgression in grapevine domestication Proc Natl Acad Sci USA. 2023 10.1073/pnas.2222041120 PMC10268302 37276420

[66] Ramu P , Esuma W, Kawuki R et al. Cassava haplotype map highlights fixation of deleterious mutations during clonal propagation. Nat Genet. 2017;49:959–63. 28416819 10.1038/ng.3845

[67] Zhang C , Yang Z, Tang D et al. Genome design of hybrid potato. Cell. 2021;184:3873–3883.e12. 34171306 10.1016/j.cell.2021.06.006

[68] Wang N , Song X, Ye J et al. Structural variation and parallel evolution of apomixis in citrus during domestication and diversification. Natl Sci Rev. 2022;9:nwac114. 36415319 10.1093/nsr/nwac114 PMC9671666

[69] Ranallo-Benavidez TR , Jaron KS, Schatz MC. GenomeScope 2.0 and Smudgeplot for reference-free profiling of polyploid genomes. Nat Commun. 2020;11:1432. 32188846 10.1038/s41467-020-14998-3 PMC7080791

[70] Marçais G , Delcher AL, Phillippy AM et al. MUMmer4: a fast and versatile genome alignment system. *PLoS Comput Biol*. 2018;14:e1005944. 10.1371/journal.pcbi.1005944 PMC5802927 29373581

[71] Simão FA , Waterhouse RM, Ioannidis P et al. BUSCO: assessing genome assembly and annotation completeness with single-copy orthologs. Bioinformatics. 2015;31:3210–2. 26059717 10.1093/bioinformatics/btv351

[72] Stanke M , Keller O, Gunduz I et al. AUGUSTUS: ab initio prediction of alternative transcripts. Nucleic Acids Res. 2006;34:W435–9. 16845043 10.1093/nar/gkl200 PMC1538822

[73] Jones P , Binns D, Chang HY et al. InterProScan 5: genome-scale protein function classification. Bioinformatics. 2014;30:1236–40. 24451626 10.1093/bioinformatics/btu031 PMC3998142

[74] Mistry J , Chuguransky S, Williams L et al. Pfam: the protein families database in 2021. Nucleic Acids Res. 2021;49:D412–d419. 33125078 10.1093/nar/gkaa913 PMC7779014

[75] Fitzkee NC , Fleming PJ, Rose GD. The protein coil library: a structural database of nonhelix, nonstrand fragments derived from the PDB. Proteins. 2005;58:852–4. 15657933 10.1002/prot.20394

[76] Flynn JM , Hubley R, Goubert C et al. RepeatModeler2 for automated genomic discovery of transposable element families. Proc Natl Acad Sci USA. 2020;117:9451–7. 32300014 10.1073/pnas.1921046117 PMC7196820

[77] Altschul SF , Gish W, Miller W et al. Basic local alignment search tool. J Mol Biol. 1990;215:403–10. 2231712 10.1016/S0022-2836(05)80360-2

[78] Yan H , Bombarely A, Li S. DeepTE: a computational method for de novo classification of transposons with convolutional neural network. Bioinformatics. 2020;36:4269–75. 32415954 10.1093/bioinformatics/btaa519

[79] Benson G . Tandem repeats finder: a program to analyze DNA sequences. Nucleic Acids Res. 1999;27:573–80. 9862982 10.1093/nar/27.2.573 PMC148217

[80] Thorvaldsdóttir H , Robinson JT, Mesirov JP. Integrative Genomics Viewer (IGV): high-performance genomics data visualization and exploration. Brief Bioinform. 2013;14:178–92. 22517427 10.1093/bib/bbs017 PMC3603213

[81] Minh BQ , Schmidt HA, Chernomor O et al. IQ-TREE 2: new models and efficient methods for phylogenetic inference in the genomic era. Mol Biol Evol. 2020;37:1530–4. 32011700 10.1093/molbev/msaa015 PMC7182206

[82] Letunic I , Bork P. Interactive tree of life (iTOL) v5: an online tool for phylogenetic tree display and annotation. Nucleic Acids Res. 2021;49:W293–6. edna 33885785 10.1093/nar/gkab301 PMC8265157

