# Supplementary material for: The complete reference genome for grapevine (Vitis vinifera L.) genetics and breeding
Source: Hortic Res. 2023 Apr 4;10(5):uhad061. doi: 10.1093/hr/uhad061 (PMC10199708; doi:10.1093/hr/uhad061)
Supplement: Web_Material_uhad061 [file web_material_uhad061.zip › FigureS1-S8_new.pdf]

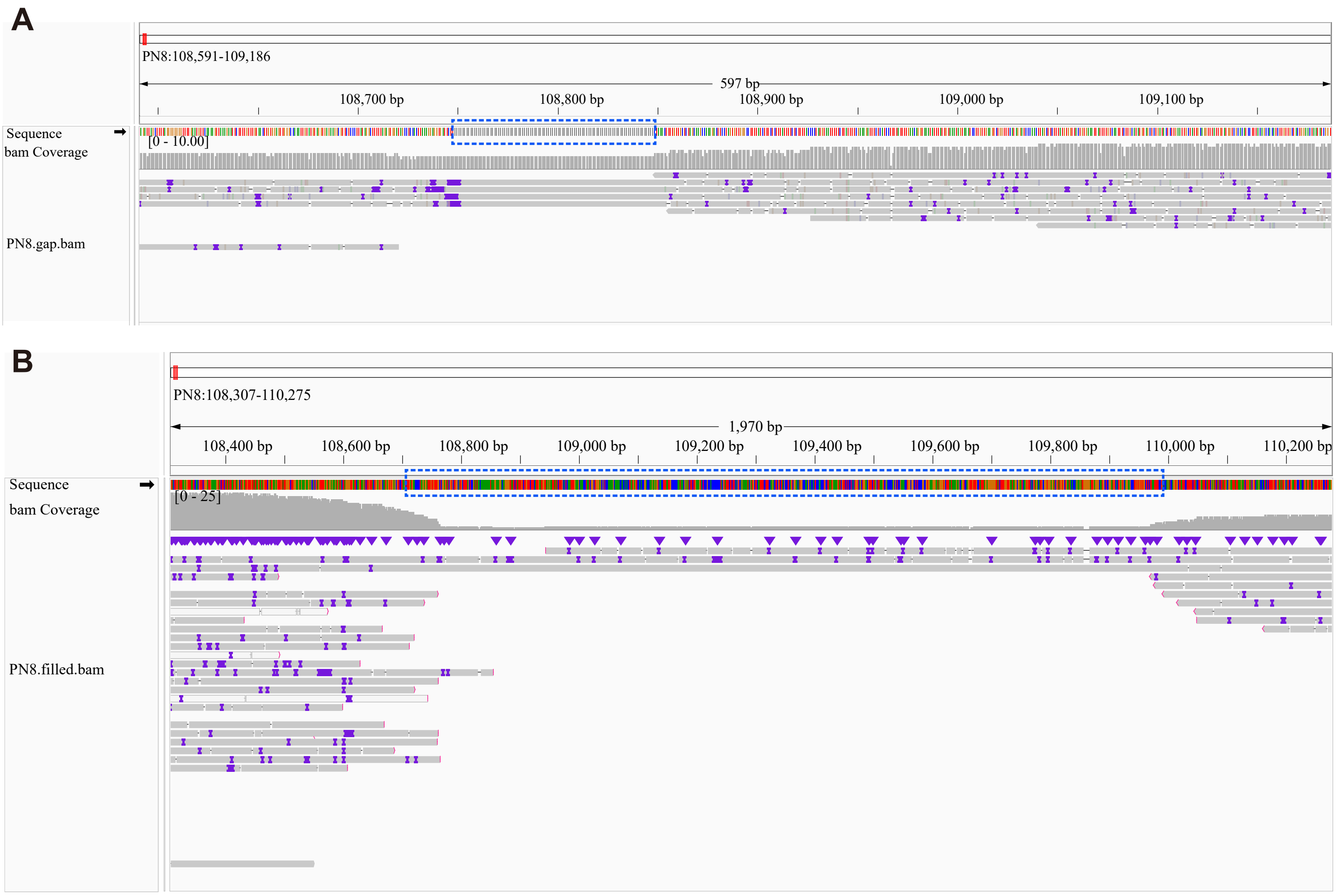

Figure S1 IGV visualization to view genome comparison results.
A.There is a gap (blue dotted line area, gap default size 100bp) at 108749bp~108848bp of chromosome 8. B. The genome alignment visualization of the 1222bp sequence filled in the gap region of A

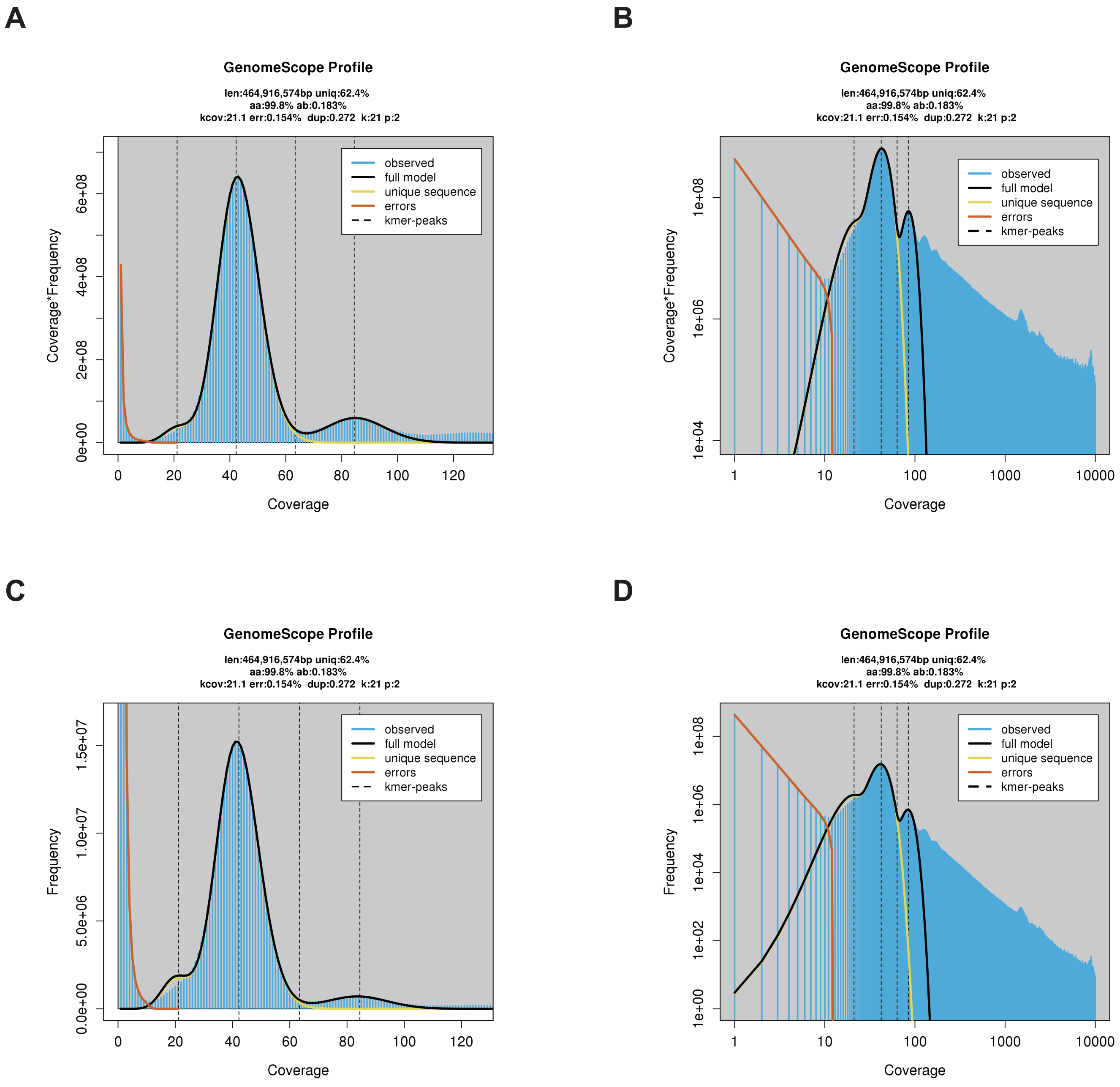

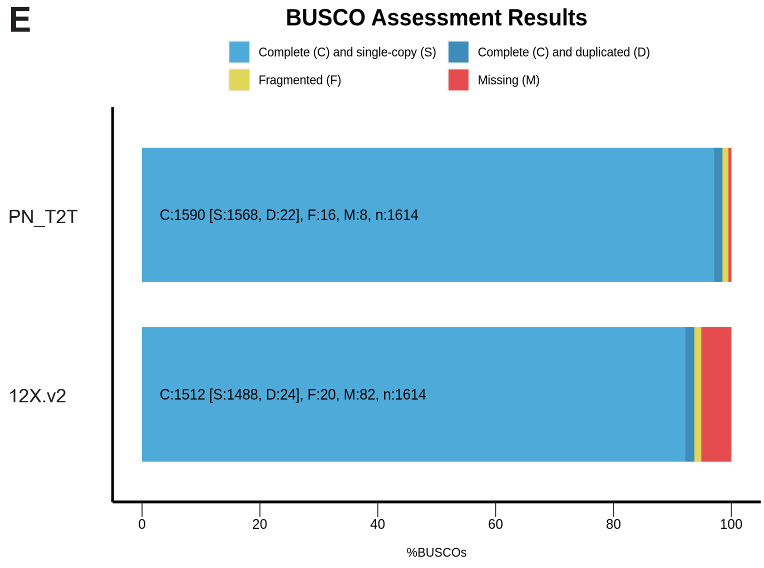

Figure S2: **Assessment of heterozygosity by K-mer and BUSCO.**

A

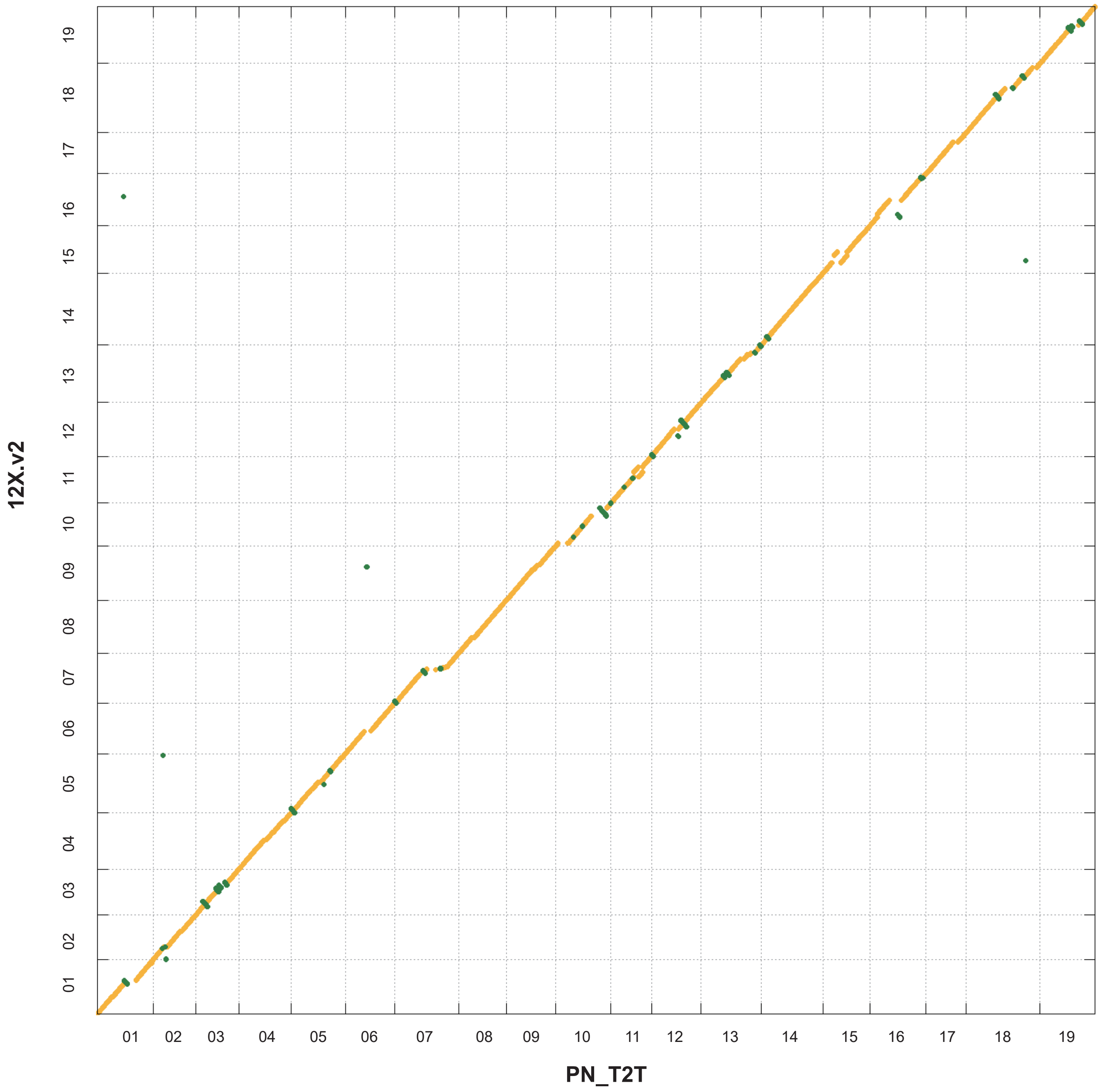

Figure S3: **Comparison of genomic collinearity between 12X.v2 and PN_T2T assembly.** Dotplot depicting reverse (green) alignments between 12X.v2 and PN_T2T.

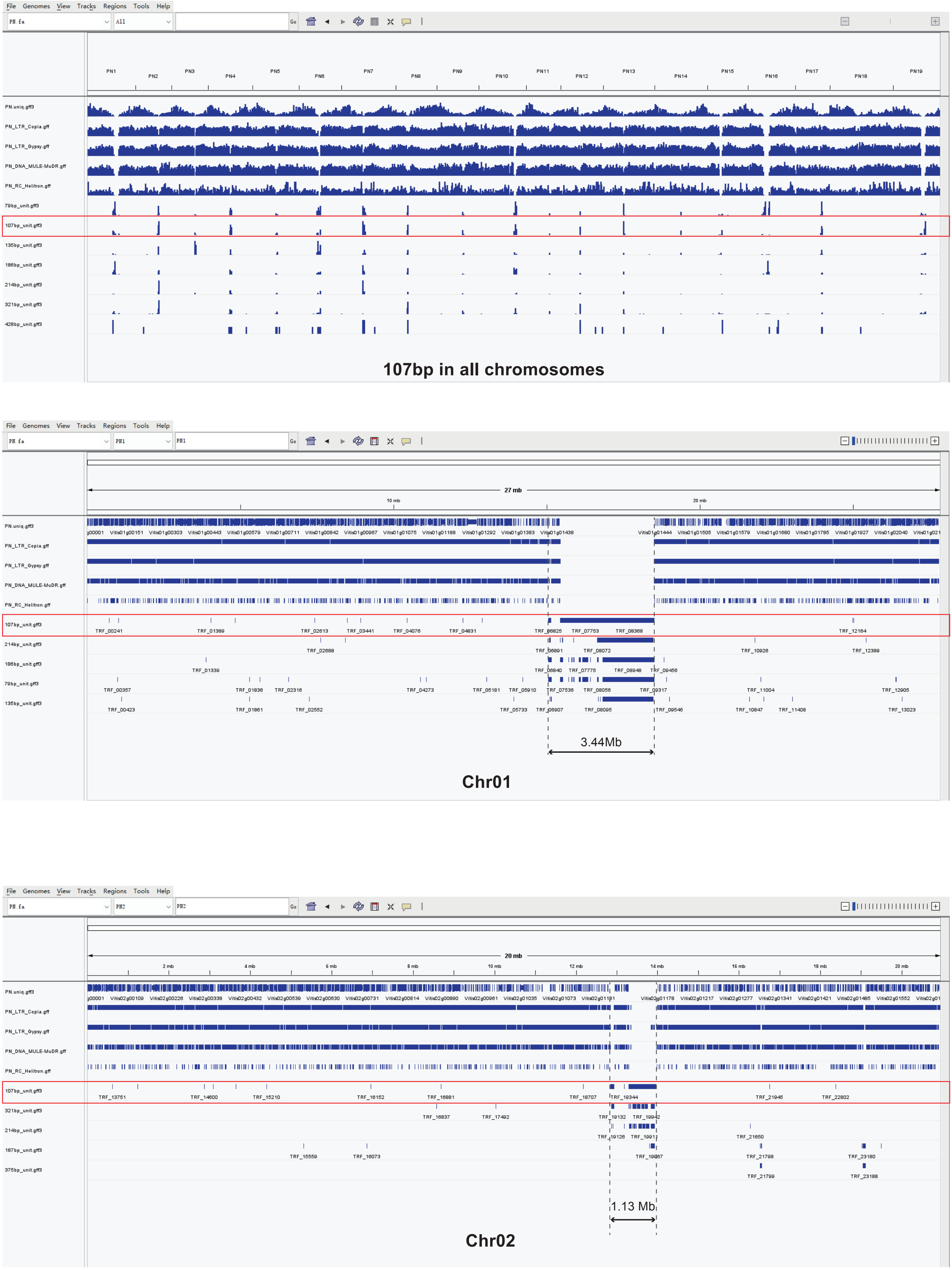

File Genomes View Tracks Regions Tools Help
PN.uniq.gff3
PN_LTR_Copia.gff
PN_LTR_Gypsy.gff
PN_DNA_MULE-MuDR.gff
PN_RC_Helitron.gff
79bp_unit.gff3
107bp_unit.gff3
135bp_unit.gff3
186bp_unit.gff3
214bp_unit.gff3
321bp_unit.gff3
428bp_unit.gff3
107bp in all chromosomes
27 mb
10 mb
20 mb
3.44Mb
Chr01
20 mb
187bp_unit.gff3
375bp_unit.gff3
1.13 Mb
Chr02

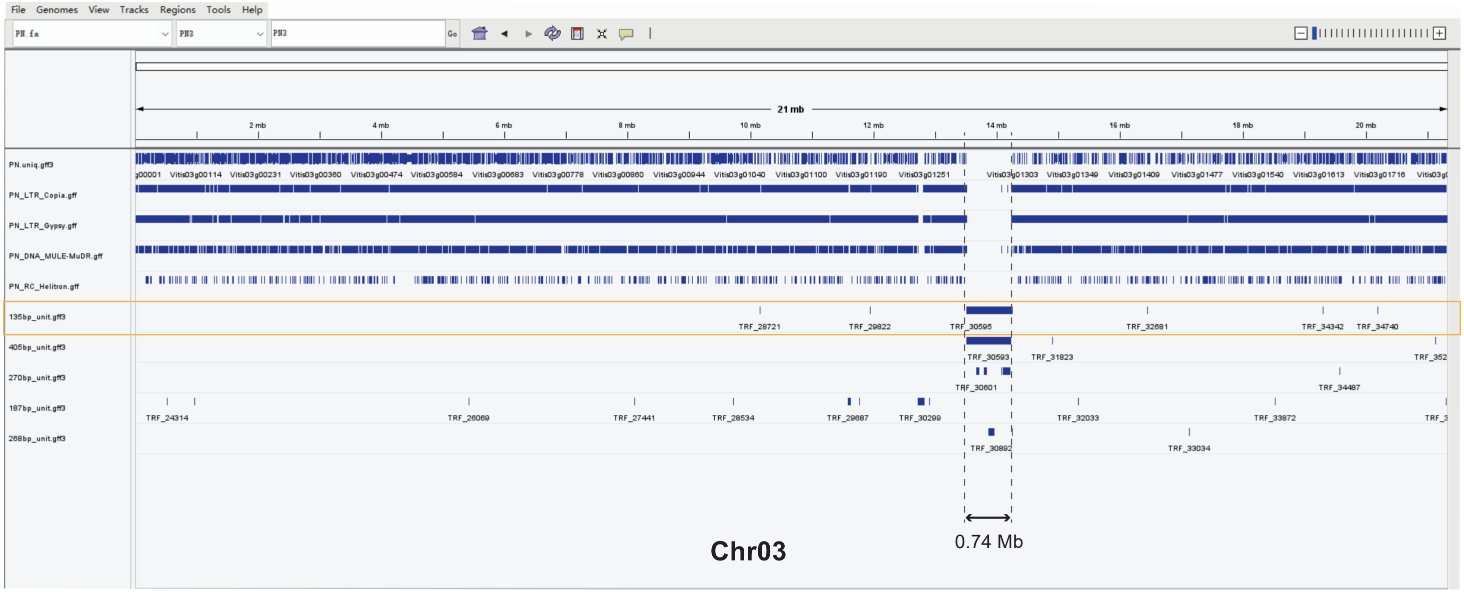
File Genomes View Tracks Regions Tools Help
21 mb
PN.uniq.gff3
PN_LTR_Copia.gff
PN_LTR_Gypsy.gff
PN_DNA_MULE-MuDR.gff
PN_RC_Helitron.gff
135bp_unit.gff3
405bp_unit.gff3
270bp_unit.gff3
187bp_unit.gff3
268bp_unit.gff3
0.74 Mb
Chr03

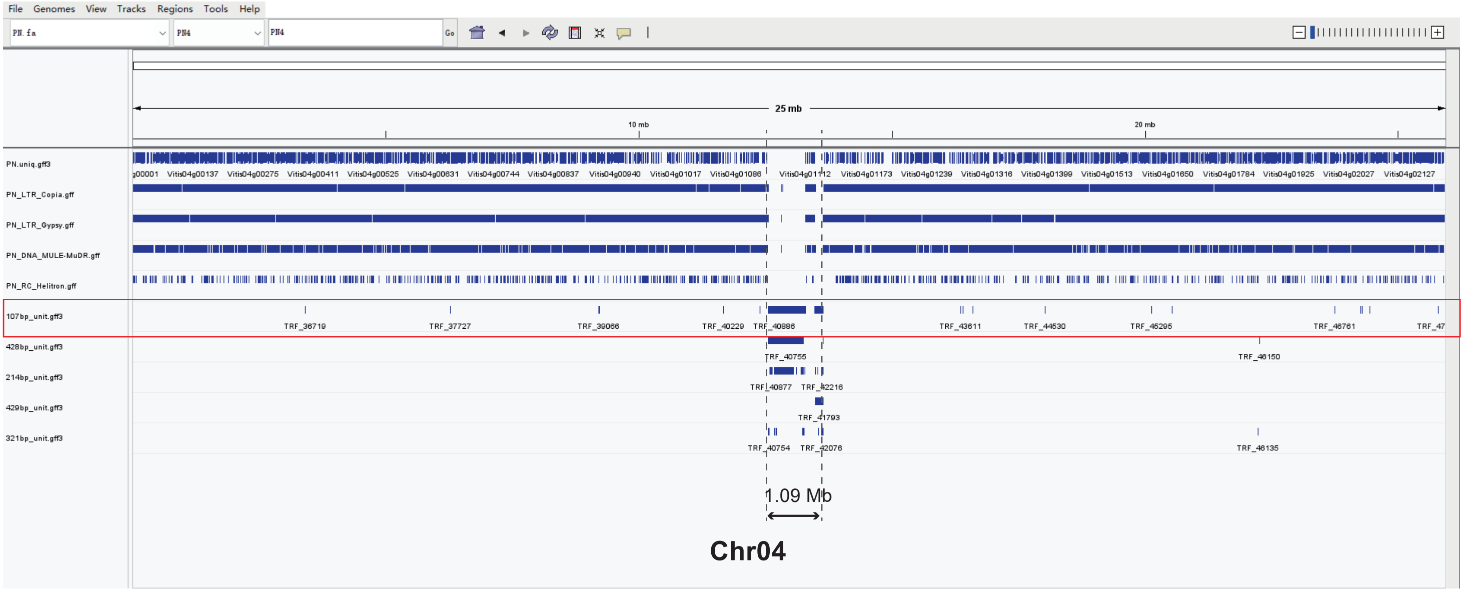
File Genomes View Tracks Regions Tools Help
25 mb
PN.uniq.gff3
PN_LTR_Copia.gff
PN_LTR_Gypsy.gff
PN_DNA_MULE-MuDR.gff
PN_RC_Helitron.gff
107bp_unit.gff3
428bp_unit.gff3
214bp_unit.gff3
429bp_unit.gff3
321bp_unit.gff3
1.09 Mb
Chr04

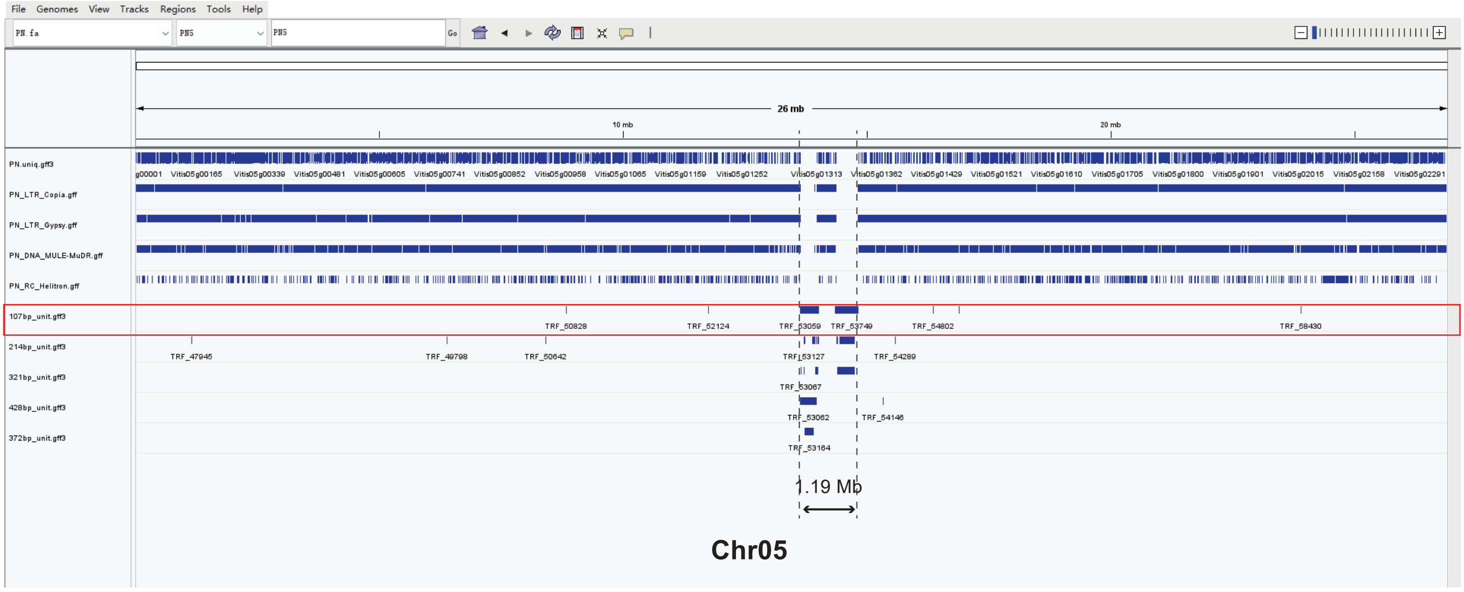
File Genomes View Tracks Regions Tools Help
26 mb
PN.uniq.gff3
PN_LTR_Copia.gff
PN_LTR_Gypsy.gff
PN_DNA_MULE-MuDR.gff
PN_RC_Helitron.gff
107bp_unit.gff3
214bp_unit.gff3
321bp_unit.gff3
428bp_unit.gff3
372bp_unit.gff3
1.19 Mb
Chr05

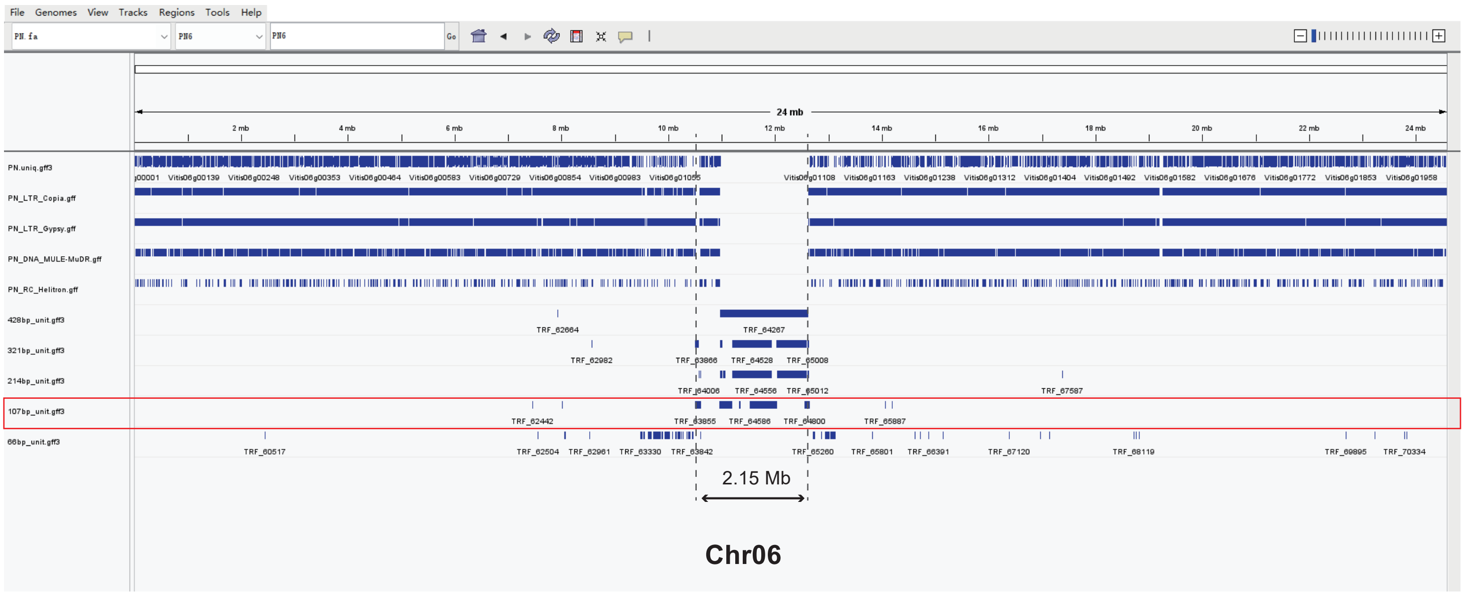
File Genomes View Tracks Regions Tools Help
PN.fa
PN6
24 mb
PN.uniq.gff3
PN_LTR_Copia.gff
PN_LTR_Gypsy.gff
PN_DNA_MULE-MuDR.gff
PN_RC_Helitron.gff
428bp_unit.gff3
321bp_unit.gff3
214bp_unit.gff3
107bp_unit.gff3
66bp_unit.gff3
2.15 Mb
Chr06

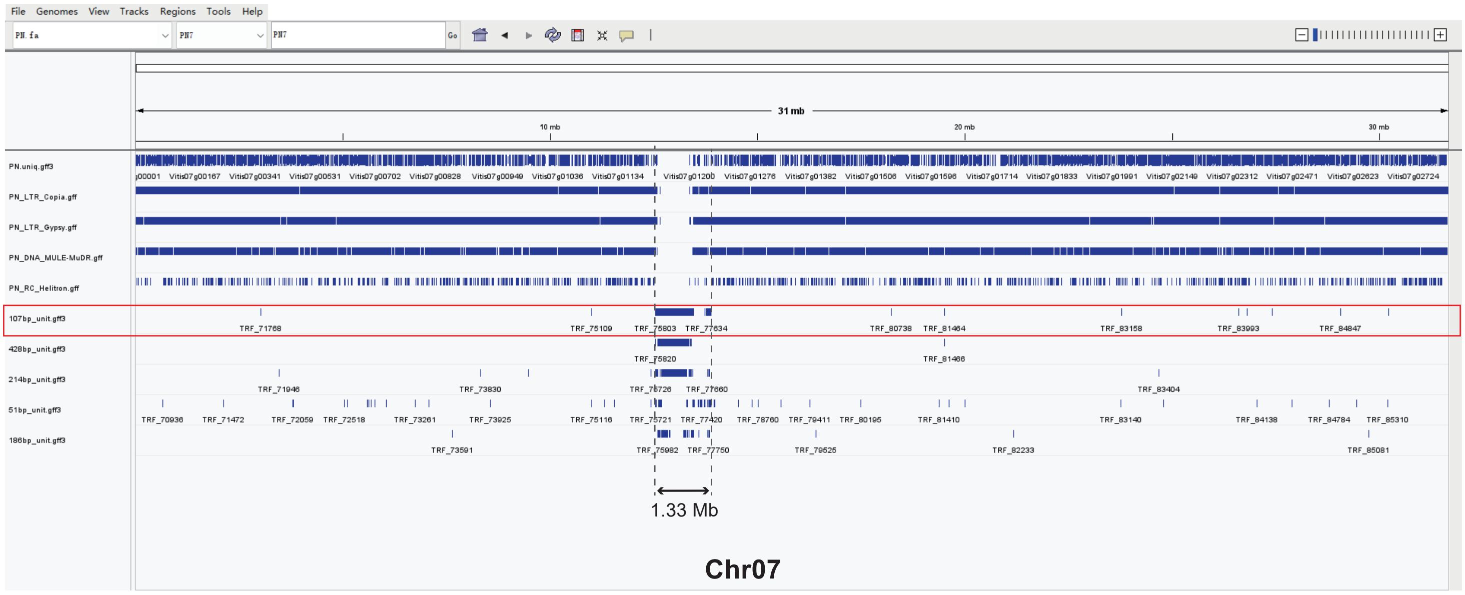
File Genomes View Tracks Regions Tools Help
PN.fa
PN7
31 mb
PN.uniq.gff3
PN_LTR_Copia.gff
PN_LTR_Gypsy.gff
PN_DNA_MULE-MuDR.gff
PN_RC_Helitron.gff
107bp_unit.gff3
428bp_unit.gff3
214bp_unit.gff3
51bp_unit.gff3
186bp_unit.gff3
1.33 Mb
Chr07

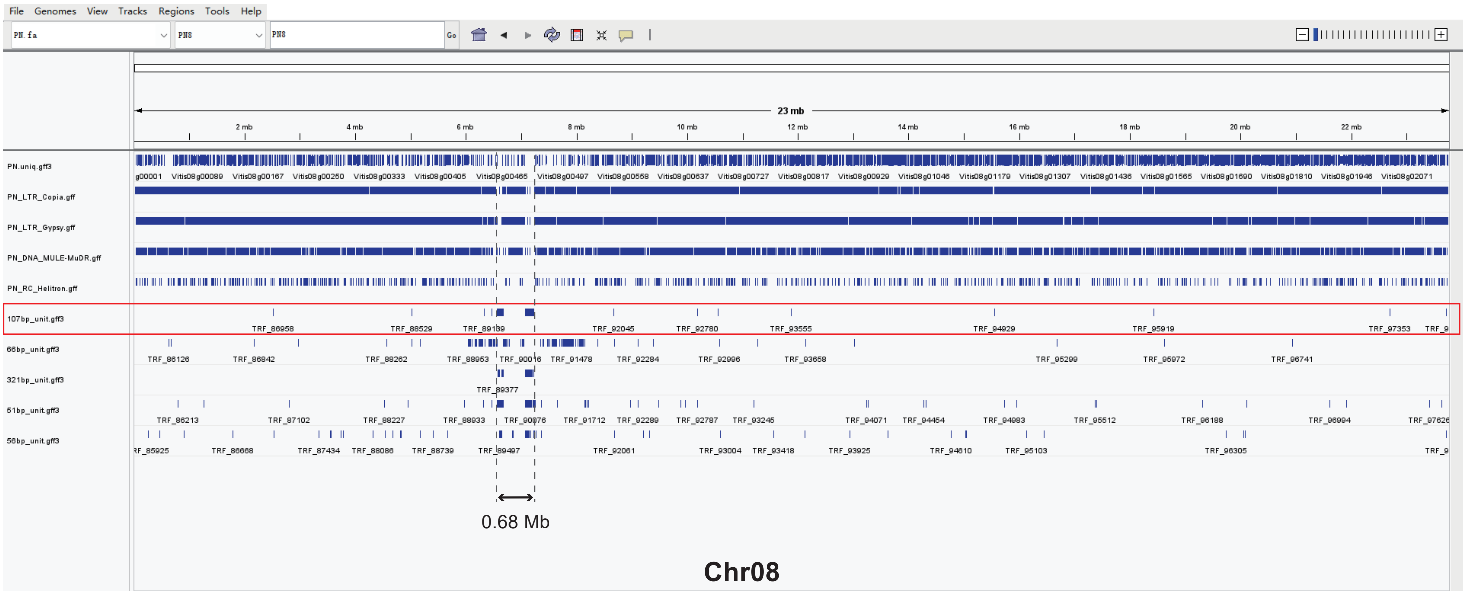
File Genomes View Tracks Regions Tools Help
PN.fa
PN8
23 mb
PN.uniq.gff3
PN_LTR_Copia.gff
PN_LTR_Gypsy.gff
PN_DNA_MULE-MuDR.gff
PN_RC_Helitron.gff
107bp_unit.gff3
66bp_unit.gff3
321bp_unit.gff3
51bp_unit.gff3
56bp_unit.gff3
0.68 Mb
Chr08

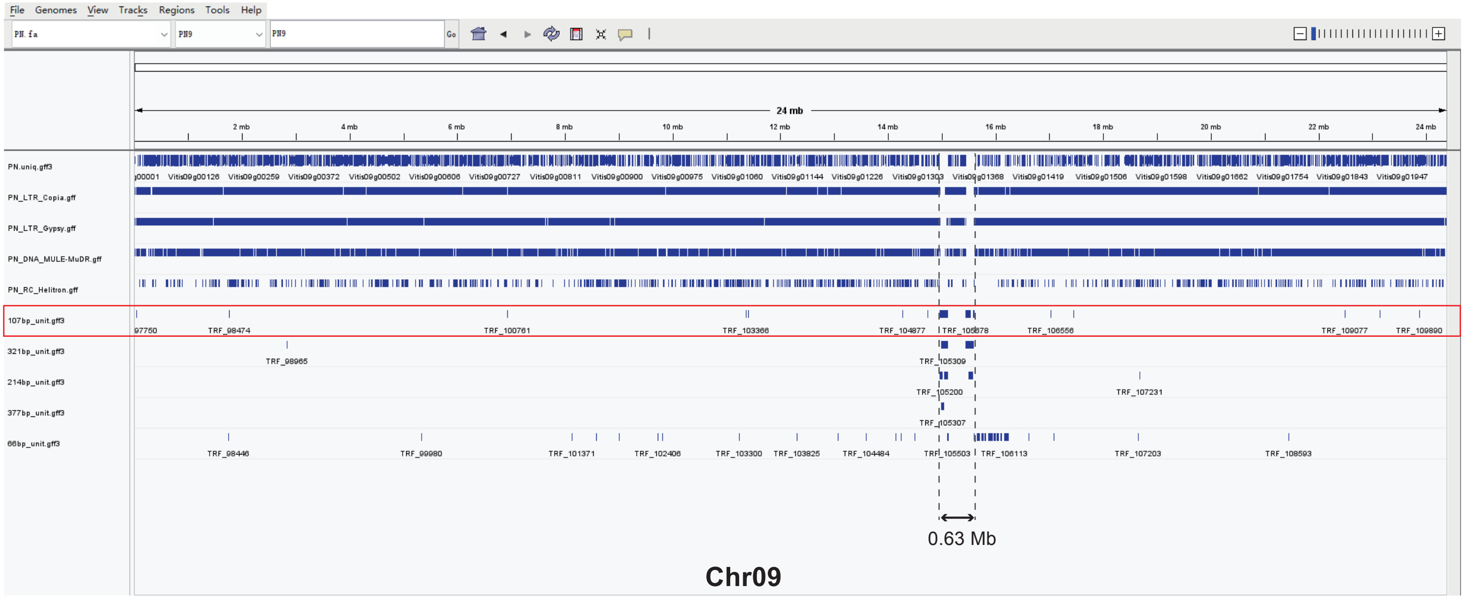
24 mb
0.63 Mb
Chr09

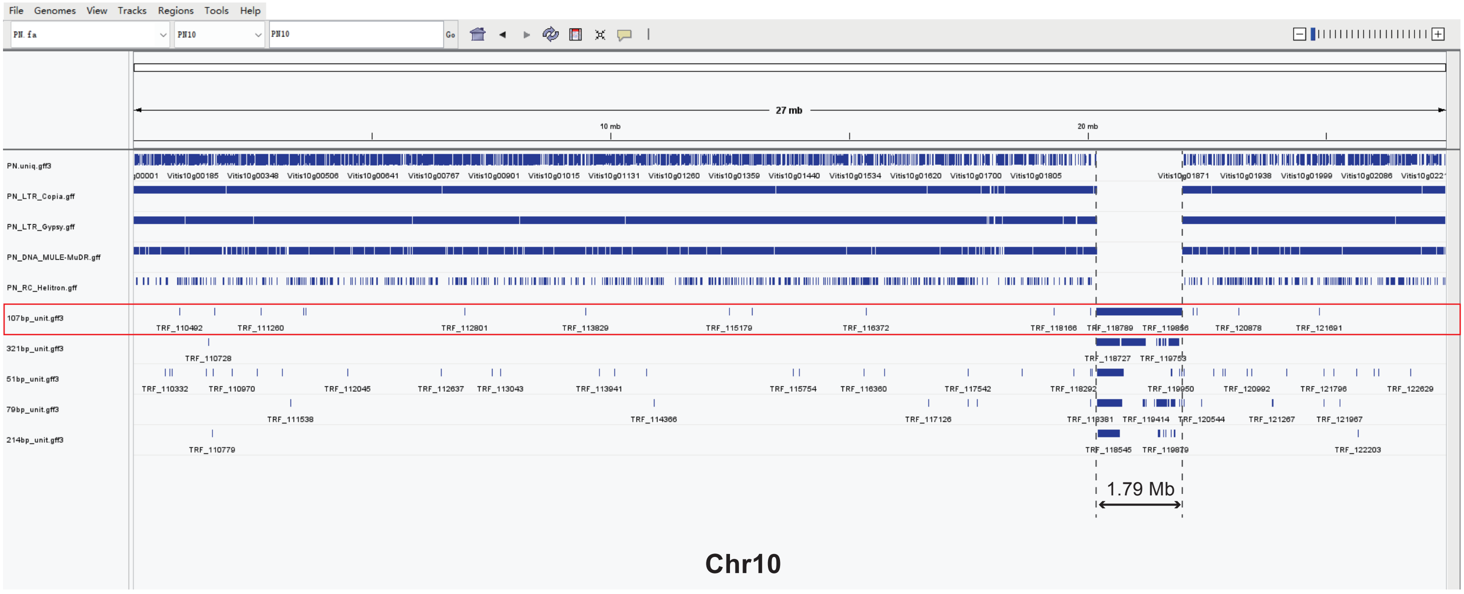
27 mb
1.79 Mb
Chr10

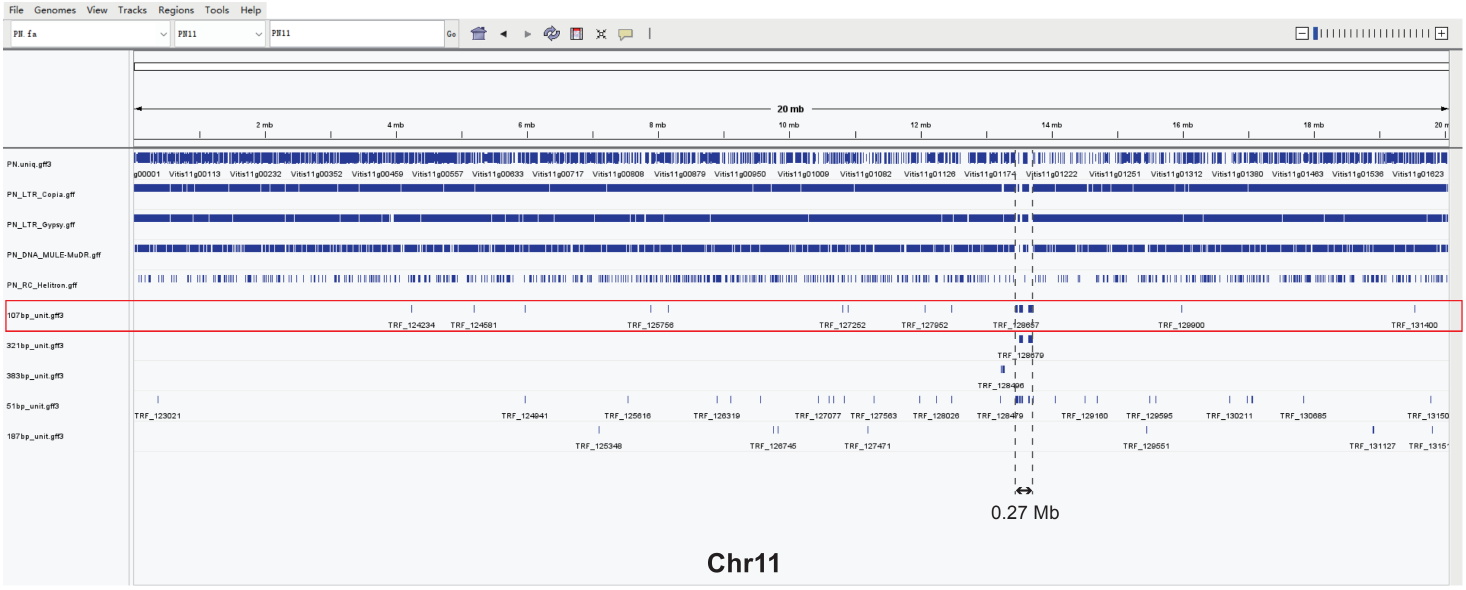
20 mb
0.27 Mb
Chr11

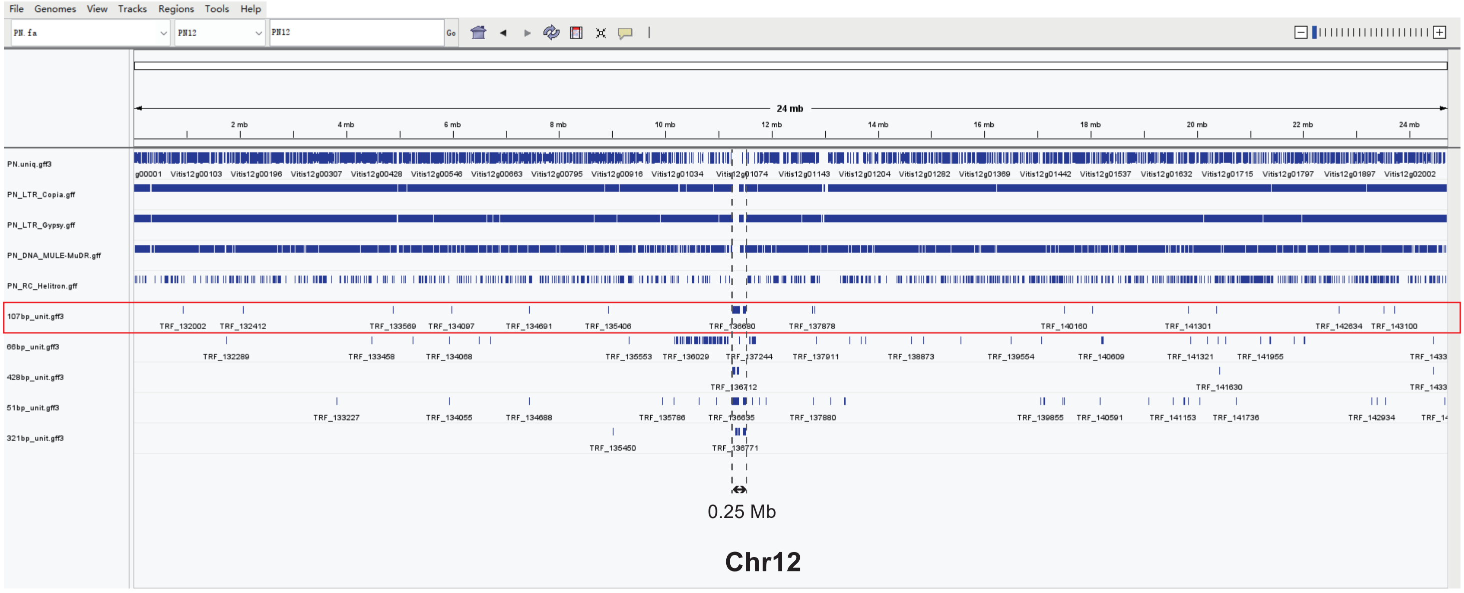
0.25 Mb
Chr12

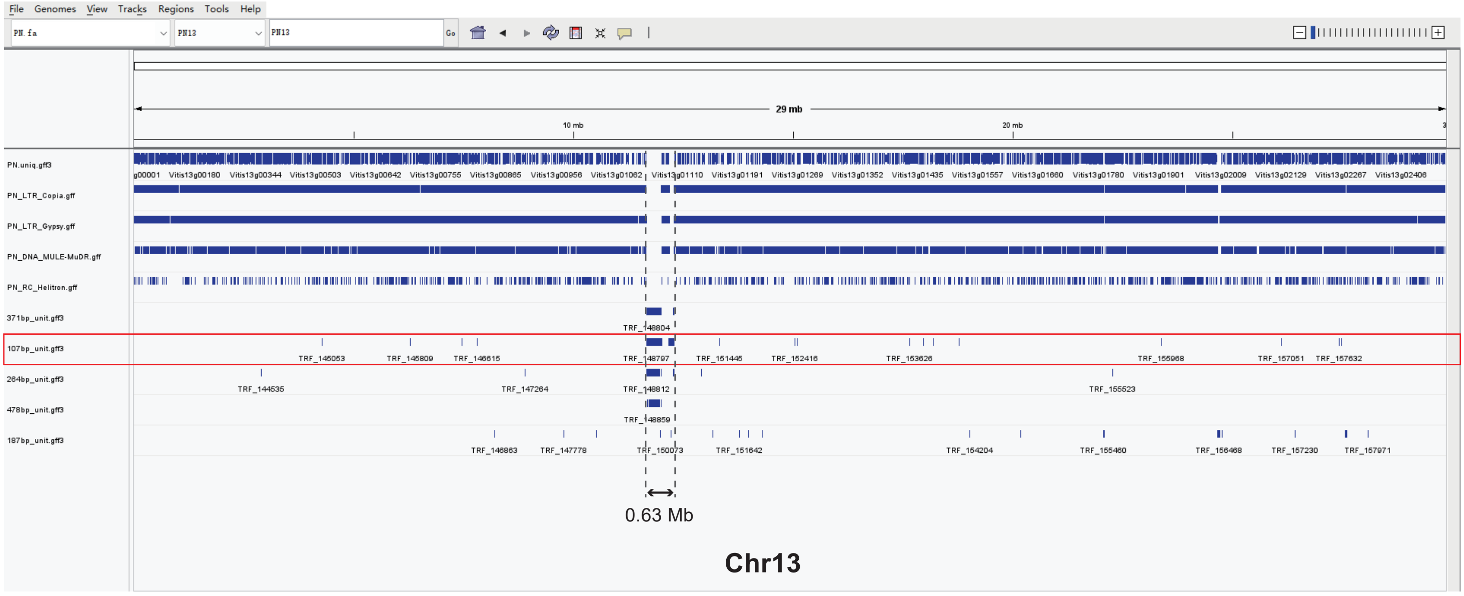
0.63 Mb
Chr13

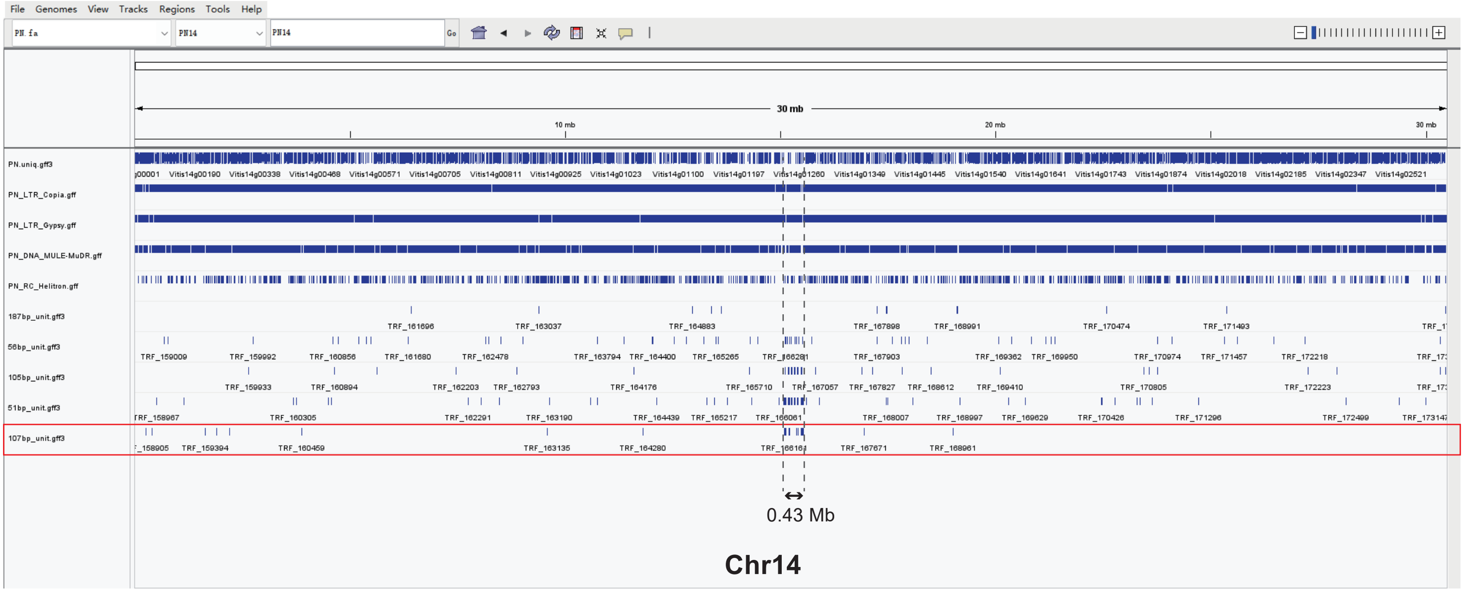
0.43 Mb
Chr14

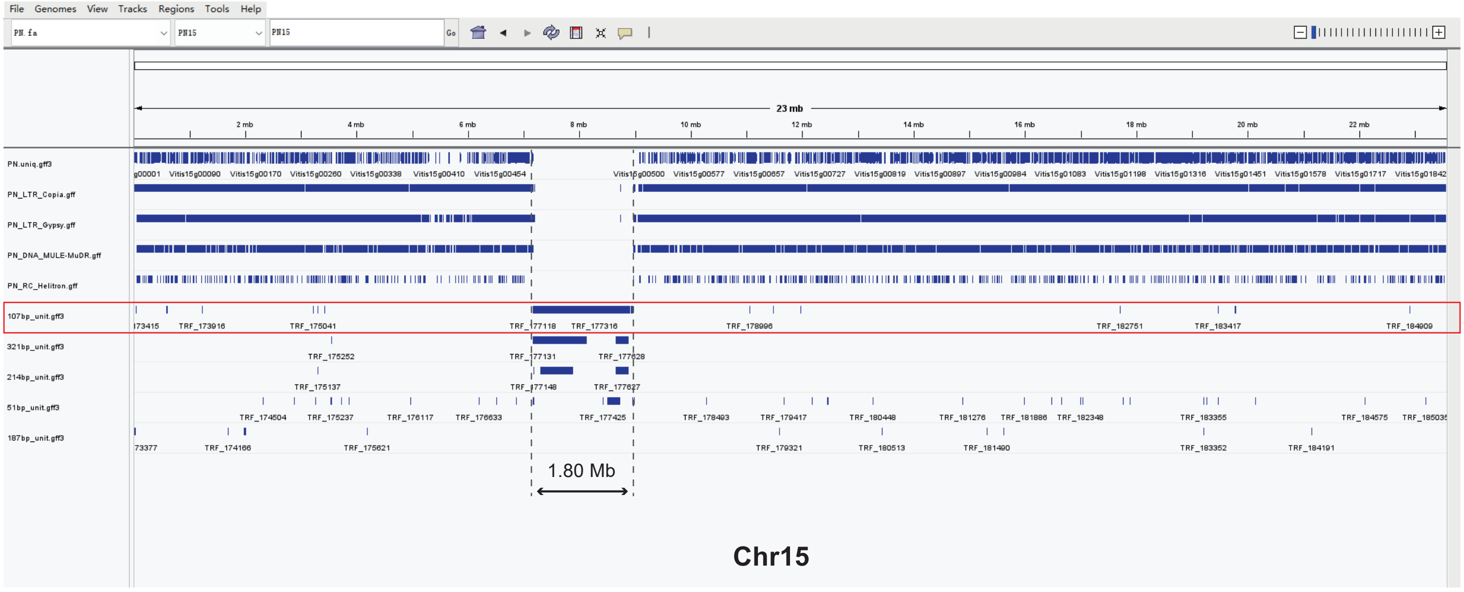
PN.uniq.gff3
PN_LTR_Copia.gff
PN_LTR_Gypsy.gff
PN_DNA_MULE-MuDR.gff
PN_RC_Helitron.gff
107bp_unit.gff3
321bp_unit.gff3
214bp_unit.gff3
51bp_unit.gff3
187bp_unit.gff3
23 mb
1.80 Mb
Chr15

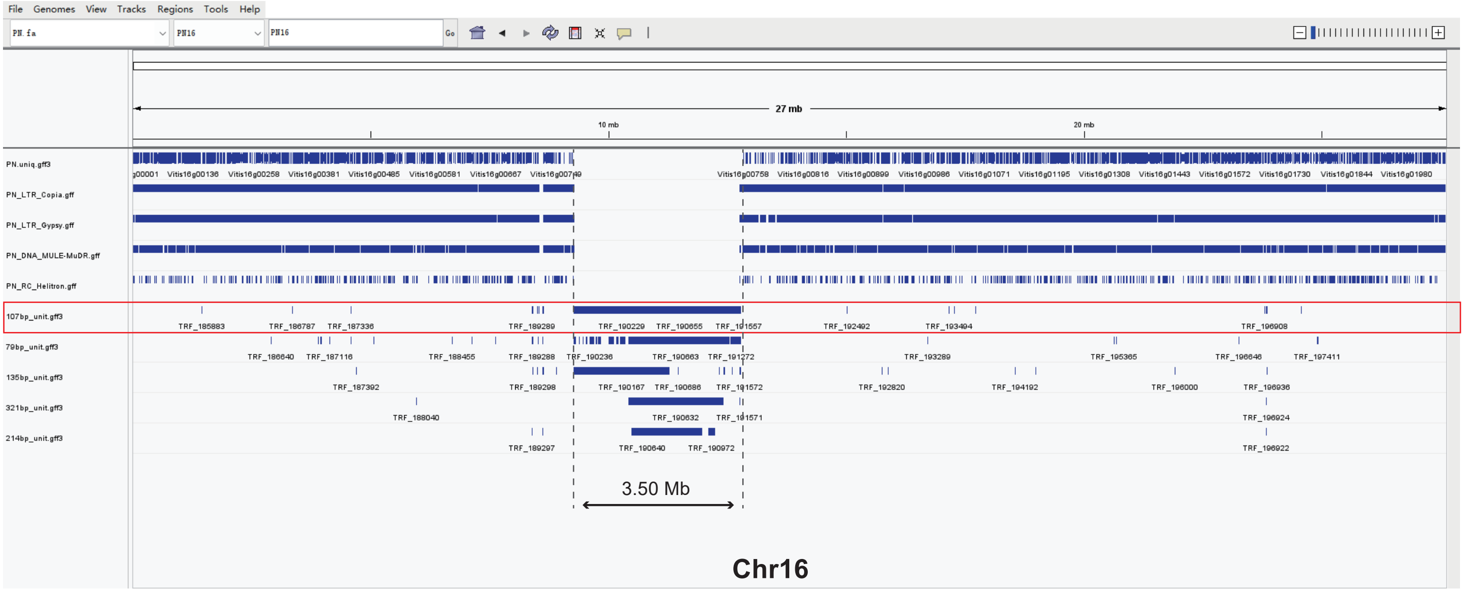
PN.uniq.gff3
PN_LTR_Copia.gff
PN_LTR_Gypsy.gff
PN_DNA_MULE-MuDR.gff
PN_RC_Helitron.gff
107bp_unit.gff3
79bp_unit.gff3
135bp_unit.gff3
321bp_unit.gff3
214bp_unit.gff3
27 mb
3.50 Mb
Chr16

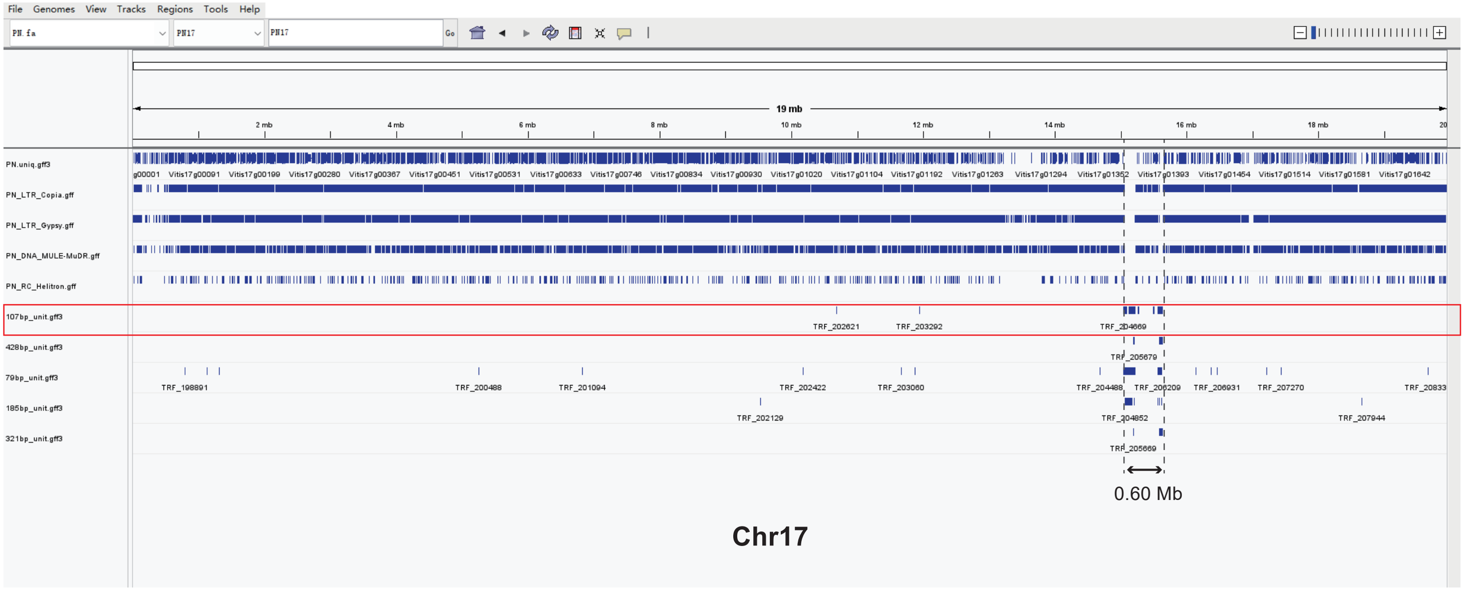
PN.uniq.gff3
PN_LTR_Copia.gff
PN_LTR_Gypsy.gff
PN_DNA_MULE-MuDR.gff
PN_RC_Helitron.gff
107bp_unit.gff3
428bp_unit.gff3
79bp_unit.gff3
185bp_unit.gff3
321bp_unit.gff3
19 mb
0.60 Mb
Chr17

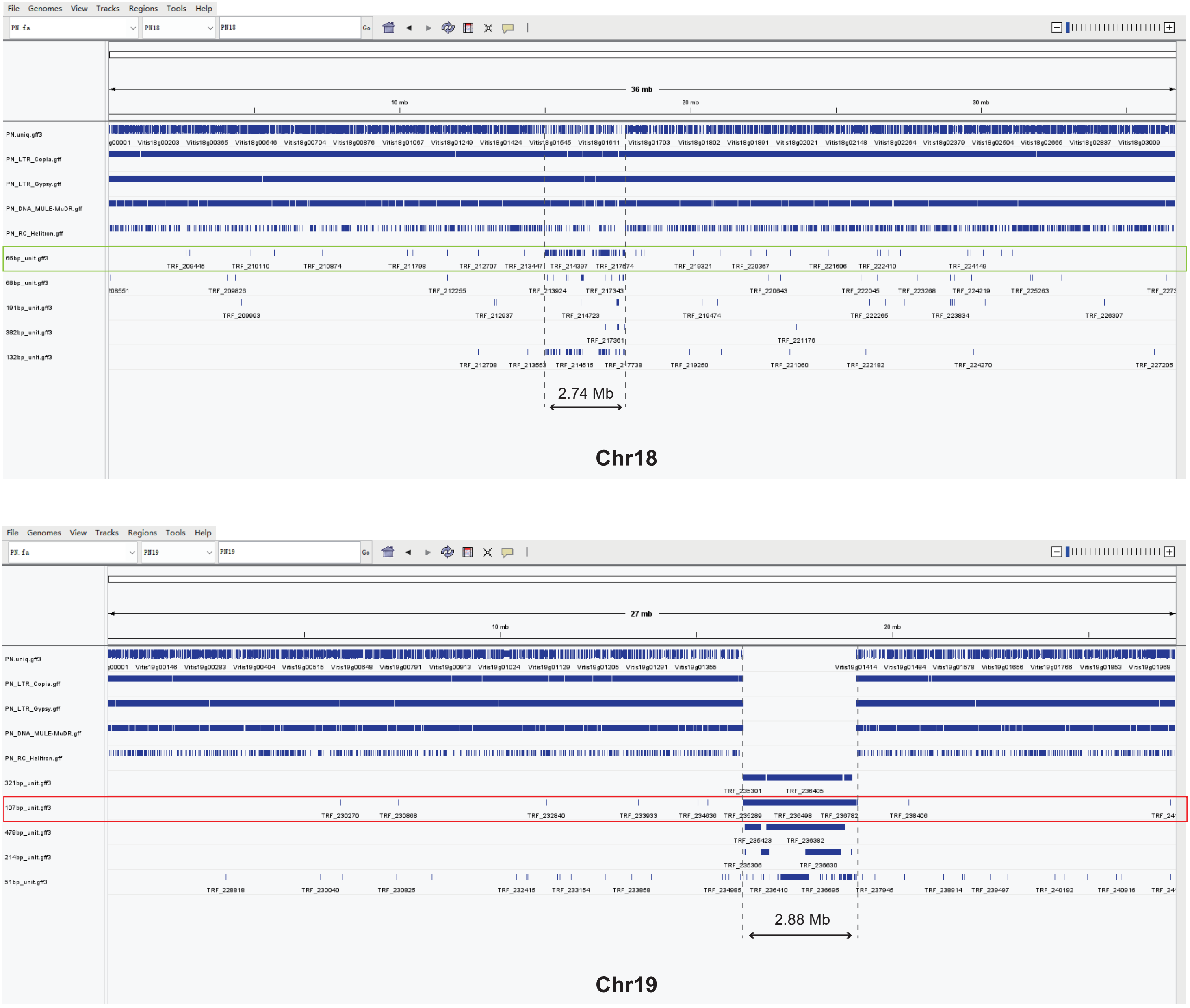

Figure S4: Visualization of TE repeats and different tandem repeat units on whole genome in IGV.

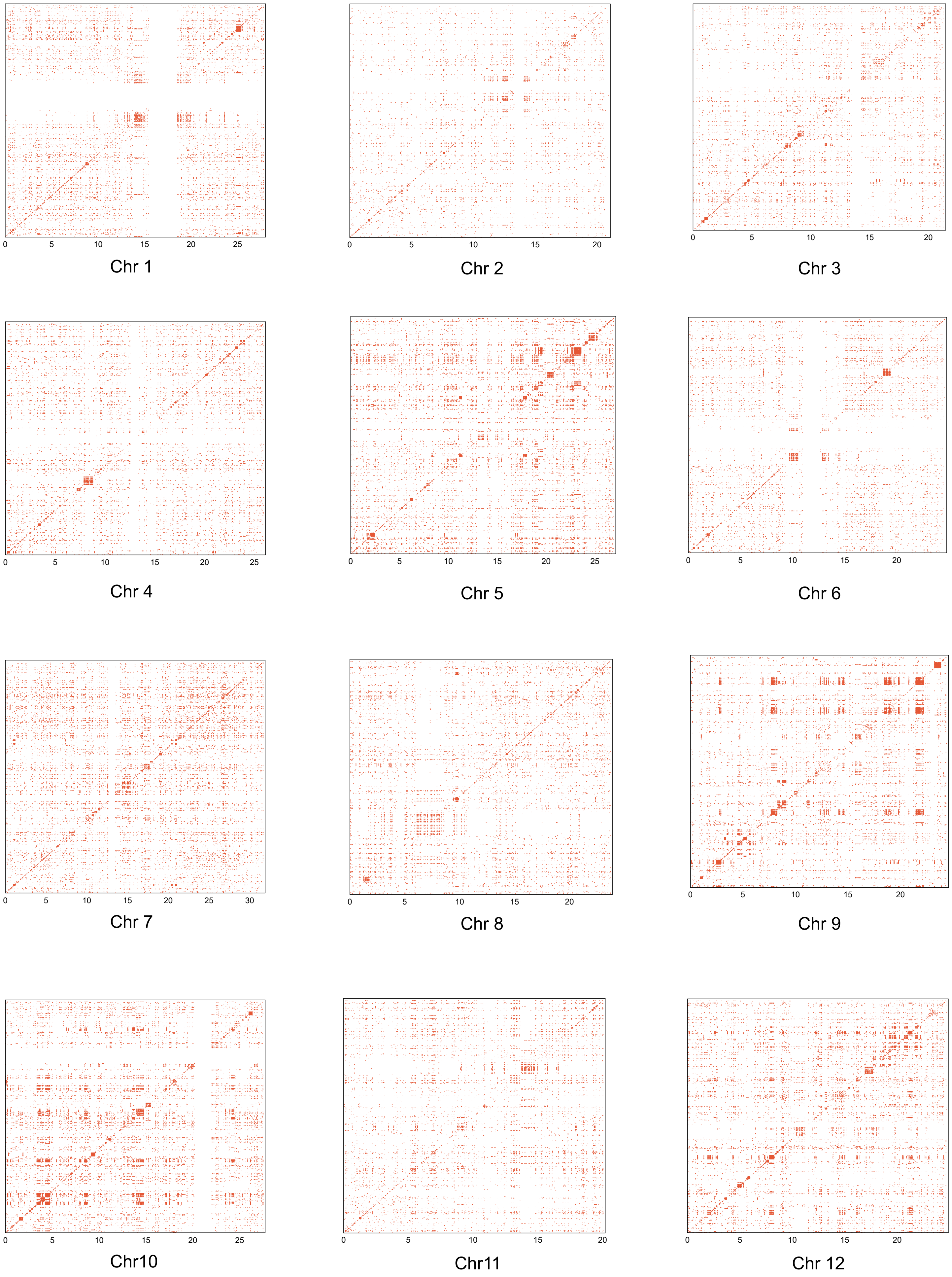

0 5 10 15 20 25
Chr 1
0 5 10 15 20
Chr 2
0 5 10 15 20
Chr 3
0 5 10 15 20 25
Chr 4
0 5 10 15 20 25
Chr 5
0 5 10 15 20
Chr 6
0 5 10 15 20 25 30
Chr 7
0 5 10 15 20
Chr 8
0 5 10 15 20
Chr 9
0 5 10 15 20 25
Chr10
0 5 10 15 20
Chr11
0 5 10 15 20
Chr 12

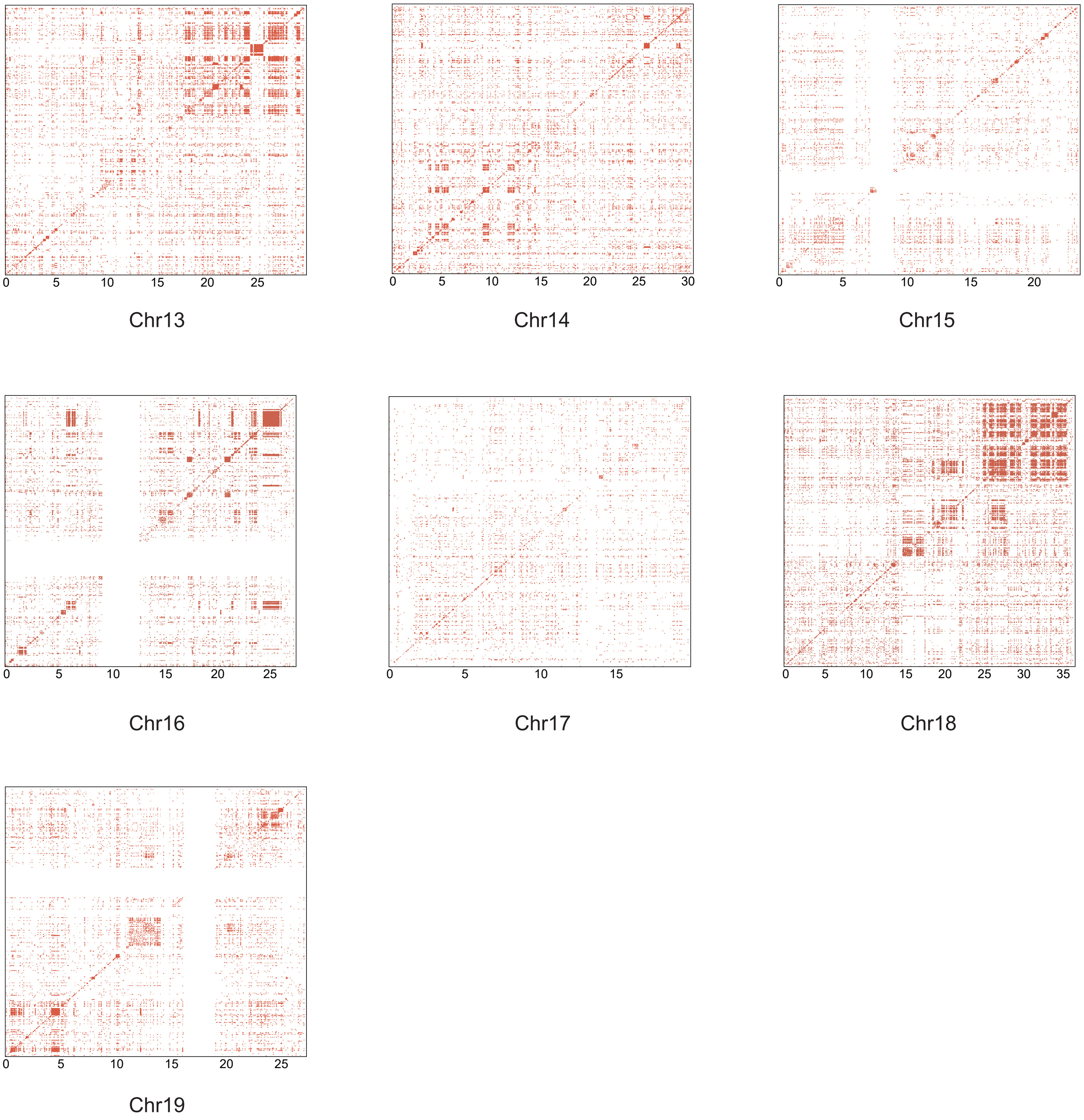

Figure S5: The gene clusters on each chromosome.

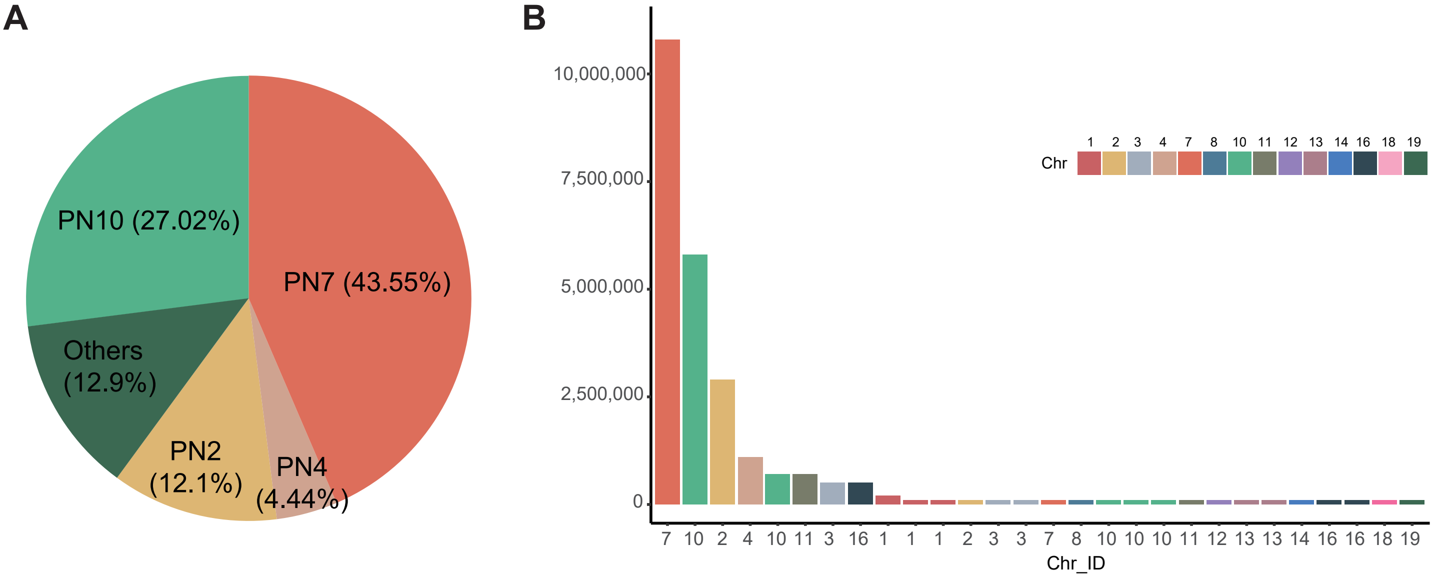

Figure S6: **Figure S5 Summary of heterozygous regions in PN40024.** A. Distribution on chromosome of the heterozygous sites that were shared in all four PN40024 samples. B. The large heterozygous fragments sorted by decreasing length.

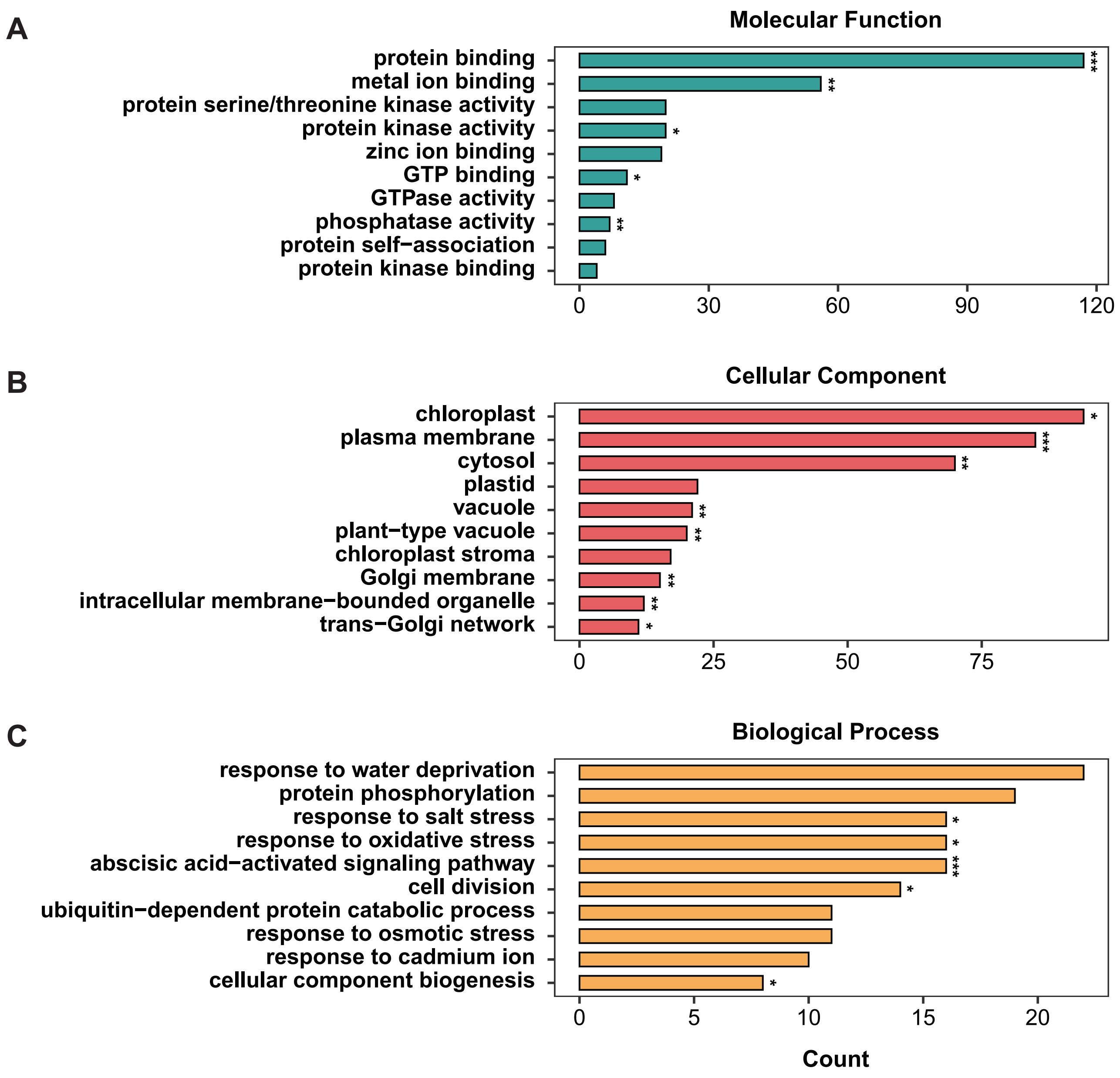

Figure S7 :GO enrichment analysis of genes contained heterozygosous sites showed in 6A.
A. Significantly enrichmented biological process. B. Significantly enrichmented GO terms in cellular component. C. Significantly enrichmented molecular function. Enrichment significant p-value: *, P< 0.05. **, P<0.01. ***, P<0.001.

A

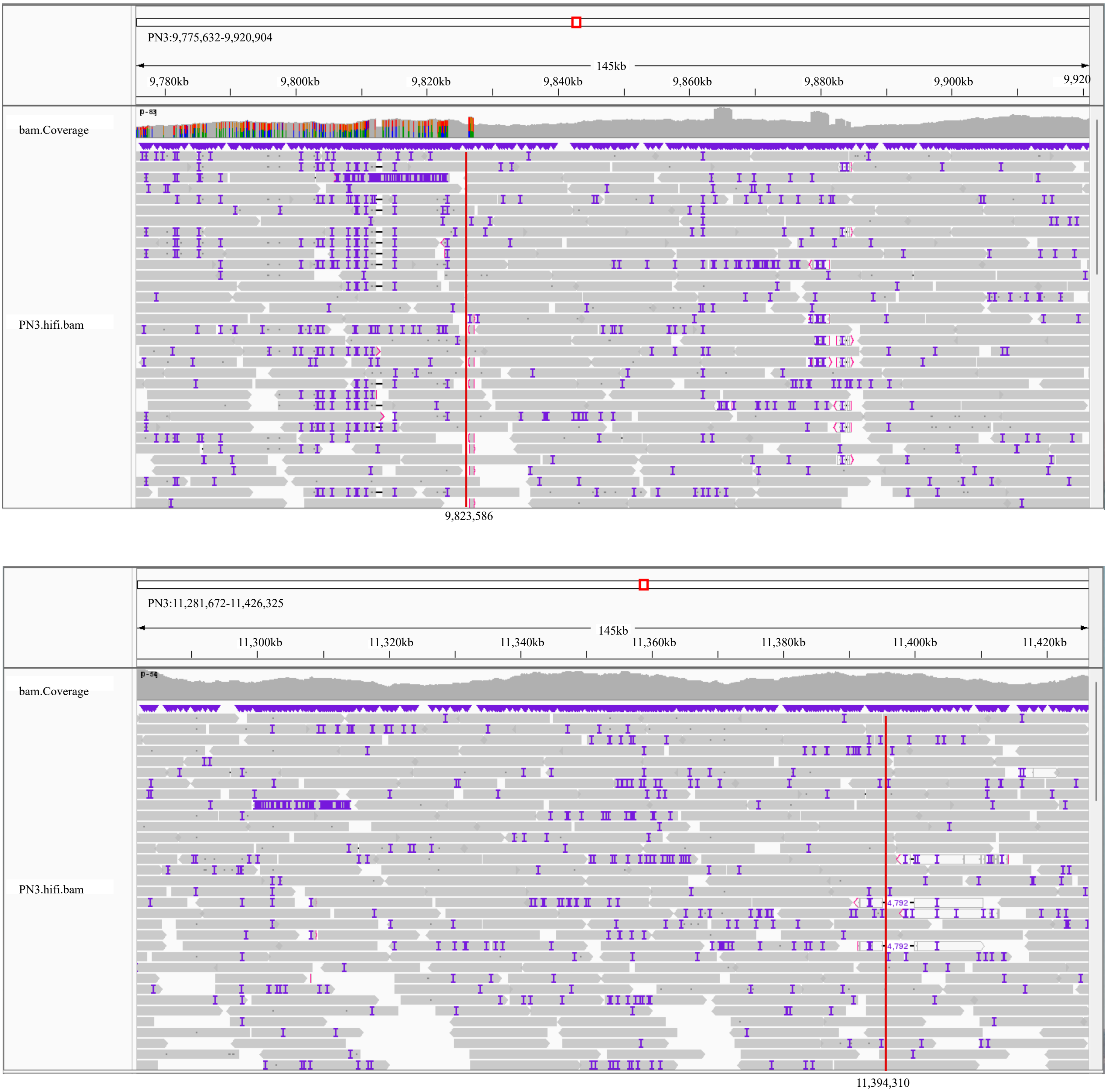
PN3:9,775,632-9,920,904
145kb
9,780kb
9,800kb
9,820kb
9,840kb
9,860kb
9,880kb
9,900kb
9,920
bam.Coverage
PN3.hifi.bam
9,823,586
PN3:11,281,672-11,426,325
145kb
11,300kb
11,320kb
11,340kb
11,360kb
11,380kb
11,400kb
11,420kb
bam.Coverage
PN3.hifi.bam
11,394,310

Chr 3

B

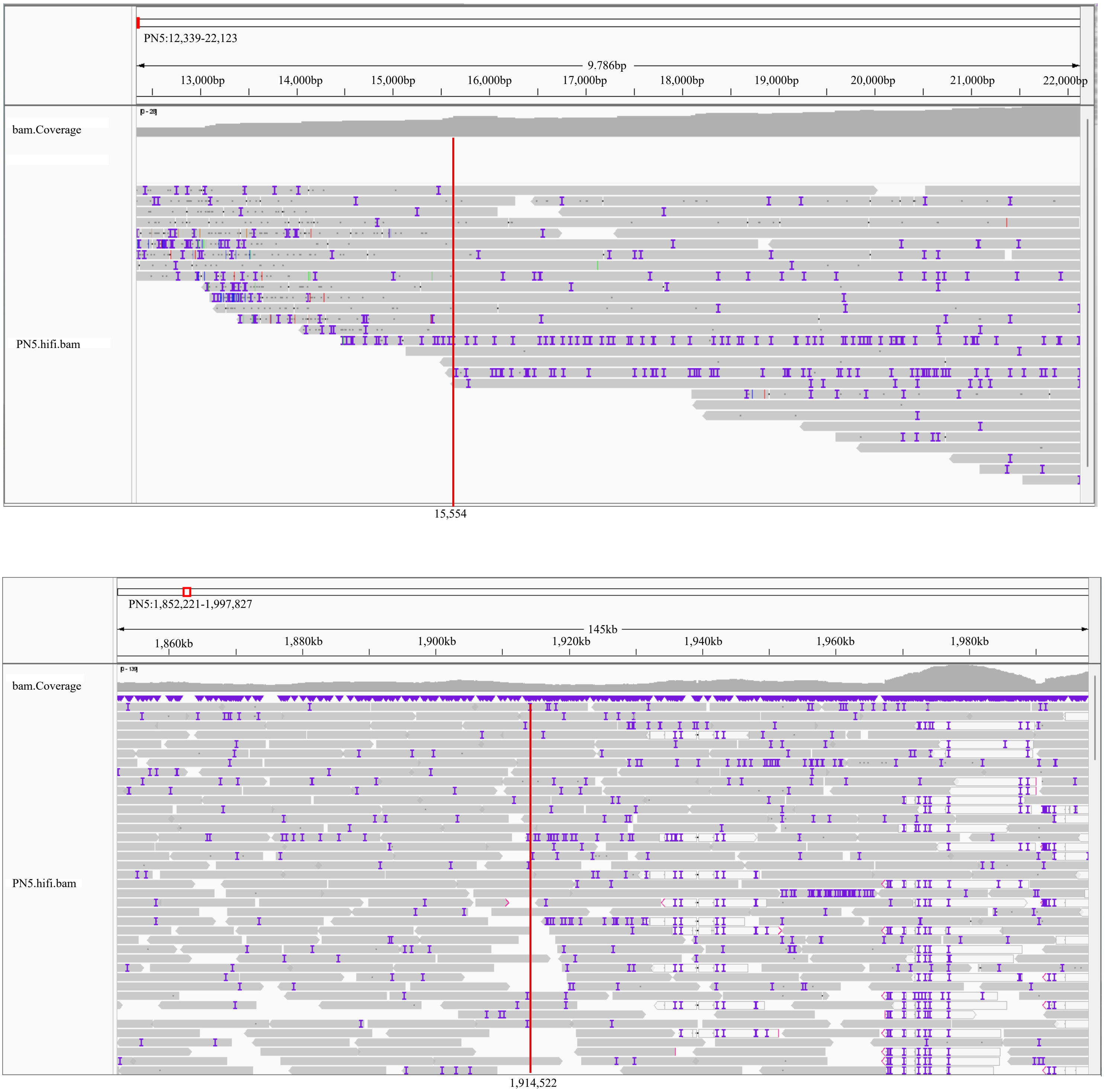
PN5:12,339-22,123
9.786bp
13,000bp
14,000bp
15,000bp
16,000bp
17,000bp
18,000bp
19,000bp
20,000bp
21,000bp
22,000bp
bam.Coverage
PN5.hifi.bam
15,554
PN5:1,852,221-1,997,827
145kb
1,860kb
1,880kb
1,900kb
1,920kb
1,940kb
1,960kb
1,980kb
bam.Coverage
PN5.hifi.bam
1,914,522

Chr 5

C

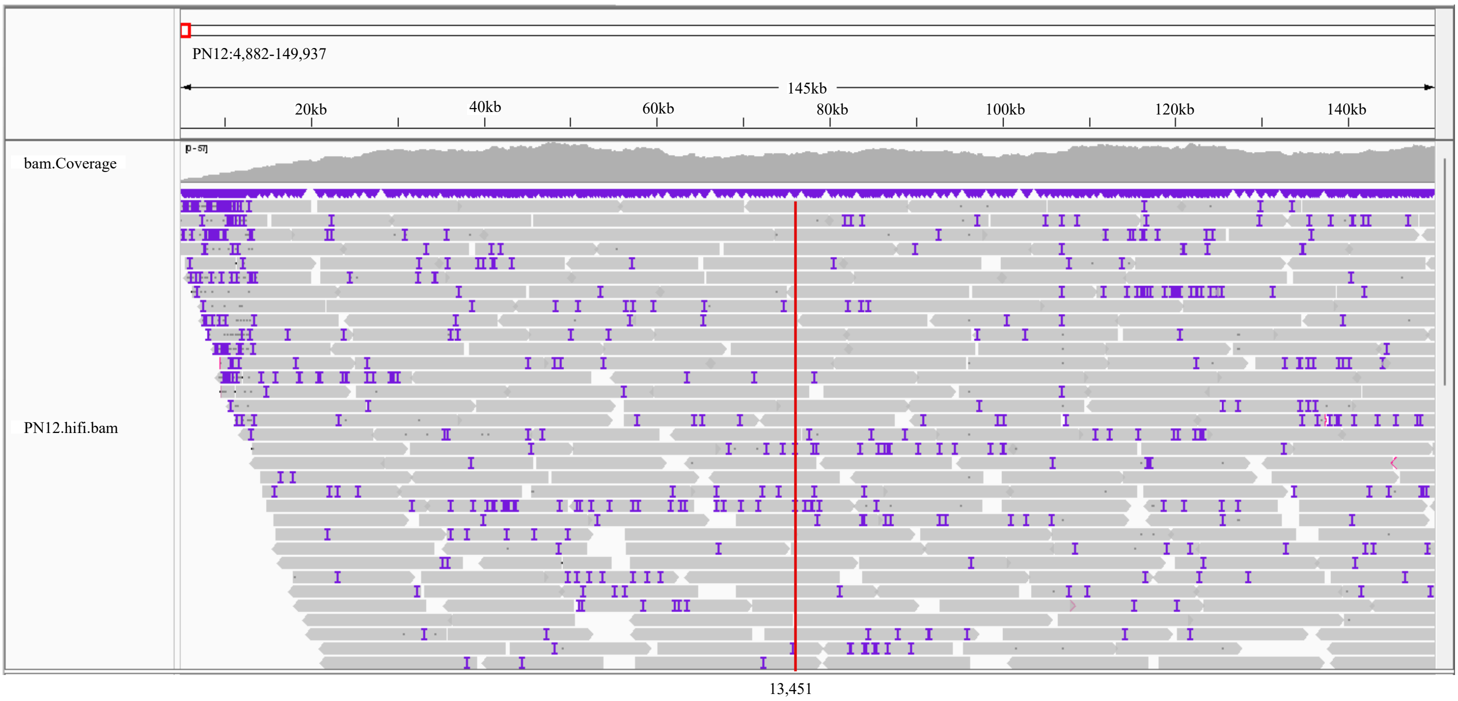

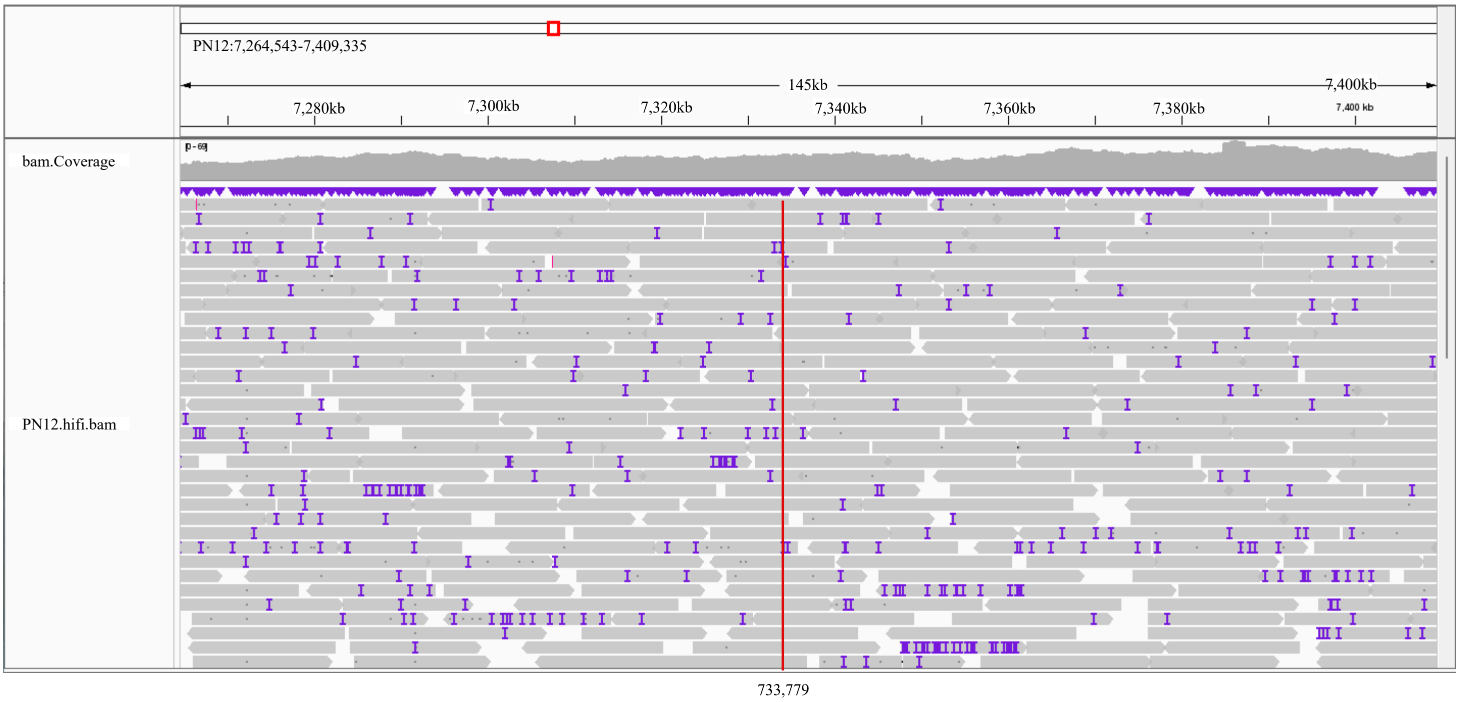

Chr12

Figure S8: Combined with the inversion shown in Fig 1A, IGV visualization was used to show whether the inversion on the 3,5,12 chromosome was real (the red line represents the breakpoint)
