# Supplementary material for: The complete reference genome for grapevine (Vitis vinifera L.) genetics and breeding
Source: Hortic Res. 2023 Apr 4;10(5):uhad061. doi: 10.1093/hr/uhad061 (PMC10199708; doi:10.1093/hr/uhad061)
Supplement: Web_Material_uhad061 [file web_material_uhad061.zip › Table S1-9.docx]

| **Table S1 Comparison of chromosome length of PN_T2T and 12X.v2 assembly.** | | |
| --- | --- | --- |
| **Chr ID** | **PN_T2T_Chr_length(bp)** | **12X.v2_Chr_length(bp)** |
| Chr01 | 27,822,162 | 23,037,639 |
| Chr02 | 20,941,263 | 18,779,844 |
| Chr03 | 21,317,290 | 19,341,862 |
| Chr04 | 25,934,928 | 23,867,706 |
| Chr05 | 26,899,771 | 25,021,643 |
| Chr06 | 24,571,969 | 21,508,407 |
| Chr07 | 31,654,362 | 21,026,613 |
| Chr08 | 23,763,023 | 22,385,789 |
| Chr09 | 24,372,199 | 23,006,712 |
| Chr10 | 27,504,061 | 18,140,952 |
| Chr11 | 20,048,508 | 19,818,926 |
| Chr12 | 24,706,008 | 22,702,307 |
| Chr13 | 29,842,242 | 24,396,255 |
| Chr14 | 30,475,315 | 30,274,277 |
| Chr15 | 23,565,456 | 20,304,914 |
| Chr16 | 27,608,946 | 22,053,297 |
| Chr17 | 19,942,836 | 17,126,926 |
| Chr18 | 36,684,271 | 29,360,087 |
| Chr19 | 27,218,600 | 24,021,853 |

**Table S2 Comparison of gene annotation among PN_T2T and PN40024.V2.1, PN40024.v4, PN40024.v4.1.**

| **Table S3 Summary statistics table of transposon annotation on 12X.v2 and PN_T2T assembly.** | | | | | |
| --- | --- | --- | --- | --- | --- |
| File name: PN_T2T | | | | | |
| sequences:19 | | | | | |
| total length: 494873210 bp | | | | | |
| GC level: 35.07 % | | | | | |
| bases masked: 328929883 bp (66.47 %) | | | | | |
|  | **Number of elements*** | | **length occupied** | | **percentage of sequence** |
| Retroelements | 498174 | | 241027616bp | | 48.71% |
| SINEs: | 358 | | 61316bp | | 0.01% |
| Penelope | 0 | | 0bp | | 0.00% |
| LINEs: | 12740 | | 5721201bp | | 1.16% |
| CRE/SLACS | 0 | | 0bp | | 0.00% |
| L2/CR1/Rex | 0 | | 0bp | | 0.00% |
| R1/LOA/Jockey | 0 | | 0bp | | 0.00% |
| R2/R4/NeSL | 0 | | 0bp | | 0.00% |
| RTE/Bov-B | 4428 | | 1048350bp | | 0.21% |
| L1/CIN4 | 8312 | | 4672851bp | | 0.94% |
| LTR elements: | 485076 | | 235245099bp | | 47.54% |
| BEL/Pao | 3433 | | 1408153bp | | 0.28% |
| Ty1/Copia | 234814 | | 97318004bp | | 19.67% |
| Gypsy/DIRS1 | 203449 | | 100065181bp | | 20.22% |
| Retroviral | 4981 | | 1201591bp | | 0.24% |
| DNA transposons | 321722 | | 52072577bp | | 10.52% |
| hobo-Activator | 164706 | | 34153977bp | | 6.90% |
| Tc1-IS630-Pogo | 242 | | 147719bp | | 0.03% |
| En-Spm | 0 | | 0bp | | 0.00% |
| MuDR-IS905 | 0 | | 0bp | | 0.00% |
| PiggyBac | 68 | | 21708bp | | 0.00% |
| Tourist/Harbinger | 29451 | | 3099958bp | | 0.63% |
| Other (Mirage,P-element, Transib) | 0 | | 0bp | | 0.00% |
| Rolling-circles | 17337 | | 6831572bp | | 1.38% |
| Unclassified: | 98550 | | 23130691bp | | 4.67% |
| Total interspersed repeats: | - | | 316230884bp | | 63.90% |
| Small RNA: | 352 | | 158634bp | | 0.03% |
| Satellites: | 0 | | 0bp | | 0.00% |
| Simple repeats: | 109280 | | 4609786bp | | 0.93% |
| Low complexity: | 22253 | | 1099007bp | | 0.22% |
| * Most repeats fragmented by insertions or deletions have been counted as one element | | | | | |
| RepeatMasker version 4.1.2-p1, sensitive mode run with rmblastn version 2.9.0+ | | | | | |
| The query was compared to classified sequences in "vitis.TElib.novel.fa" | | | | | |
| FamDB: | | | | | |
|  | |  | |  |  |
| File name: 12X.v2 | | | | | |
| sequences: 1907 | | | | | |
| total length: 486196837 bp | | | | | |
| GC level: 34.56 % | | | | | |
| bases masked: 303719475 bp (62.47 %) | | | | | |
|  | | **Number of elements*** | **length occupied** | | **percentage of sequence** |
| Retroelements | | 503687 | 217819122bp | | 44.80% |
| SINEs: | | 382 | 69835bp | | 0.01% |
| Penelope | | 0 | 0bp | | 0.00% |
| LINEs: | | 12965 | 5631535bp | | 1.16% |
| CRE/SLACS | | 0 | 0bp | | 0.00% |
| L2/CR1/Rex | | 0 | 0bp | | 0.00% |
| R1/LOA/Jockey | | 0 | 0bp | | 0.00% |
| R2/R4/NeSL | | 0 | 0bp | | 0.00% |
| RTE/Bov-B | | 4441 | 1057810bp | | 0.22% |
| L1/CIN4 | | 8524 | 4573725bp | | 0.94% |
| LTR elements: | | 490340 | 212117752 bp | | 43.63% |
| BEL/Pao | | 3446 | 1357749bp | | 0.28% |
| Ty1/Copia | | 237308 | 96042963bp | | 19.75% |
| Gypsy/DIRS1 | | 205714 | 97817275bp | | 20.12% |
| Retroviral | | 4982 | 1194678bp | | 0.25% |
| DNA transposons | | 322614 | 50858685bp | | 10.46% |
| hobo-Activator | | 164692 | 33100992bp | | 6.81% |
| Tc1-IS630-Pogo | | 242 | 148072bp | | 0.03% |
| En-Spm | | 0 | 0bp | | 0.00% |
| MuDR-IS905 | | 0 | 0bp | | 0.00% |
| PiggyBac | | 71 | 24045bp | | 0.00% |
| Tourist/Harbinger | | 29620 | 3101762bp | | 0.64% |
| Other (Mirage,P-element, Transib) | | 0.00% | 0bp | | 0.00% |
| Rolling-circles | | 17310 | 6761562bp | | 1.39% |
| Unclassified: | | 98485 | 22850239bp | | 4.70% |
| Total interspersed repeats: | | - | 291528046bp | | 59.96% |
| Small RNA: | | 345 | 101346bp | | 0.02% |
| Satellites: | | 0 | 0bp | | 0.00% |
| Simple repeats: | | 110117 | 4226495bp | | 0.87% |
| Low complexity: | | 22543 | 1102026bp | | 0.23% |
| * Most repeats fragmented by insertions or deletions have been counted as one element | | | | | |
| RepeatMasker version 4.1.2-p1, sensitive mode run with rmblastn version 2.9.0+ | | | | | |
| The query was compared to classified sequences in "vitis.TElib.novel.fa" | | | | | |
| FamDB: | | | | | |

| **Table S4 Characterization of telomeres on each chromosome.** | | | | | | | | | | |
| --- | --- | --- | --- | --- | --- | --- | --- | --- | --- | --- |
|  | **CCCTAAA** | | | | | | **TTTAGGG** | | | |
|  | **Start_position** | | **End_position** | **Repeat_number** | **Repeat_length** | **Start_position** | | **End_position** | **Repeat_number** | **Repeat_length** |
| Chr01 | | - | 10,000 | 644 | 4,508 | 27,810,000 | | 27,822,162 | 2,958 | 20,706 |
| Chr02 | | - | 20,000 | 1,364 | 9,548 | 20,860,000 | | 20,941,263 | 1,924 | 13,468 |
| Chr03 | | - | 10,000 | 812 | 5,684 | 21,300,000 | | 21,317,290 | 750 | 5,250 |
| Chr04 | | - | 10,000 | 780 | 5,460 | 25,920,000 | | 25,934,928 | 2,089 | 14,623 |
| Chr05 | | - | 20,000 | 2,137 | 14,959 | 26,870,000 | | 26,899,771 | 733 | 5,131 |
| Chr06 | | - | 70,000 | 744 | 5,208 | 24,520,000 | | 24,571,969 | 2,010 | 14,070 |
| Chr07 | | - | 10,000 | 180 | 1,260 | 31,650,000 | | 31,654,362 | 443 | 3,101 |
| Chr08 | | - | 40,000 | 4,479 | 31,353 | 23,750,000 | | 23,761,901 | 950 | 6,650 |
| Chr09 | | - | 50,000 | 1,935 | 13,545 | 24,340,000 | | 24,372,199 | 1,406 | 9,842 |
| Chr10 | | - | 170,000 | 1,607 | 11,249 | 27,500,000 | | 27,504,061 | 1,659 | 11,613 |
| Chr11 | | - | 20,000 | 1,610 | 11,270 | 20,040,000 | | 20,048,508 | 1,502 | 10,514 |
| Chr12 | | - | 20,000 | 1,948 | 13,636 | 24,690,000 | | 24,706,008 | 1,623 | 11,361 |
| Chr13 | | - | 20,000 | 1,685 | 11,795 | 29,830,000 | | 29,842,242 | 1,931 | 13,517 |
| Chr14 | | - | 80,000 | 1,912 | 13,384 | 30,460,000 | | 30,475,315 | 790 | 5,530 |
| Chr15 | | - | - | - | - | 23,550,000 | | 23,565,456 | 828 | 5,796 |
| Chr16 | | - | 10,000 | 467 | 3,269 | 27,600,000 | | 27,608,946 | 2,077 | 14,539 |
| Chr17 | | - | 20,000 | 1,621 | 11,347 | - | | - | - | - |
| Chr18 | | - | 10,000 | 1,191 | 8,337 | 36,670,000 | | 36,684,271 | 1,529 | 10,703 |
| Chr19 | | - | 40,000 | 416 | 2,912 | 27,220,000 | | 27,218,600 | 1,159 | 8,113 |

| **Table S5 Summary statistics table of centromeres.** | | | | | | | |
| --- | --- | --- | --- | --- | --- | --- | --- |
| **Chr ID** | **Start** | **End** | **Centromirc length** | **Start_TRF** | **End_TRF** | **Filter** | **107bp sequence alignment** |
| Chr01 | 15058323 | 18498588 | 3,440,265 | ID=TRF_06825 | ID=TRF_09345 | 107bp (copies > 2.0) | TRF_07753_copies_7111.3 |
| Chr02 | 12853764 | 13981401 | 1,127,637 | ID=TRF_19105 | ID=TRF_20483 | 107bp (copies > 2.0) | TRF_19588_copies_375.3 |
| Chr03 | 13509959 | 14254027 | 744,068 | ID=TRF_30595 | ID=TRF_31500 | 135bp (copies > 2.0) | TRF_30739_copies_2.5 |
| Chr04 | 12561177 | 13649342 | 1,088,165 | ID=TRF_40749 | ID=TRF_42295 | 107bp (copies > 2.0) | TRF_41210_copies_759.4 |
| Chr05 | 13637421 | 14825803 | 1,188,382 | ID=TRF_53059 | ID=TRF_53862 | 107bp (copies > 2.0) | TRF_53640_copies_1749.9 |
| Chr06 | 10502173 | 12649428 | 2,147,255 | ID=TRF_63855 | ID=TRF_65173 | 107bp (copies > 2.0) | TRF_64525_copies_1237.3 |
| Chr07 | 12546375 | 13875549 | 1,329,174 | ID=TRF_75803 | ID=TRF_78106 | 107bp (copies > 2.0) | TRF_76815_copies_824.5 |
| Chr08 | 6566735 | 7245262 | 678,527 | ID=TRF_89332 | ID=TRF_90951 | 107bp (copies > 2.0) | TRF_90879_copies_133.1 |
| Chr09 | 14971629 | 15597964 | 626,335 | ID=TRF_105186 | ID=TRF_105752 | 107bp (copies > 2.0) | TRF_105692_copies_528.5 |
| Chr10 | 20196477 | 21984661 | 1,788,184 | ID=TRF_118448 | ID=TRF_120156 | 107bp (copies > 2.0) | TRF_119325_copies_6090.1 |
| Chr11 | 13446850 | 13717651 | 270,801 | ID=TRF_128657 | ID=TRF_128888 | 107bp (copies > 2.0) | TRF_128874_copies_490.9 |
| Chr12 | 11260923 | 11513970 | 253,047 | ID=TRF_136680 | ID=TRF_137229 | 107bp (copies > 2.0) | TRF_136763_copies_366.4 |
| Chr13 | 11666622 | 12291982 | 625,360 | ID=TRF_148797 | ID=TRF_150855 | 107bp (copies > 2.0) | TRF_149131_copies_179.5 |
| Chr14 | 15103386 | 15538063 | 434,677 | ID=TRF_166161 | ID=TRF_166927 | 107bp (copies > 2.0) | TRF_166199_copies_26.2 |
| Chr15 | 7170066 | 8965387 | 1,795,321 | ID=TRF_177118 | ID=TRF_177807 | 107bp (copies > 2.0) | TRF_177316_copies_6920.0 |
| Chr16 | 9285205 | 12780129 | 3,494,924 | ID=TRF_189988 | ID=TRF_191598 | 107bp (copies > 2.0) | TRF_190655_copies_8766.8 |
| Chr17 | 15042439 | 15647460 | 605,021 | ID=TRF_204669 | ID=TRF_206544 | 107bp (copies > 2.0) | TRF_206544_copies_145.9 |
| Chr18 | 15013184 | 17754640 | 2,741,456 | ID=TRF_213834 | ID=TRF_217810 | 66bp (copies > 2.0) | TRF_221779_copies_4.1 |
| Chr19 | 16196018 | 19078334 | 2,882,316 | ID=TRF_235289 | ID=TRF_237744 | 107bp (copies > 2.0) | TRF_236640_copies_7272.1 |

Repeat sequence: centromere.fasta

| **Table S6 Summary of different repeat units on centromeres.** | | | | | | | | | | | | | | |
| --- | --- | --- | --- | --- | --- | --- | --- | --- | --- | --- | --- | --- | --- | --- |
|  | **unit** | **copies** | **length** |  | **unit** | **copies** | **length** | |  | | **unit** | | **copies** | **length** |
| **chr01** | 107 | 29886.5 | 3197856 | **chr08** | 107 | 2569.4 | 274925.8 | | **chr15** | | 107 | | 17011.5 | 1820231 |
|  | 214 | 8643 | 1849602 |  | 66 | 2499.2 | 164947.2 | |  | | 321 | | 3725 | 1195725 |
|  | 186 | 9358.9 | 1740755 |  | 321 | 359 | 115239 | |  | | 214 | | 3816.2 | 816666.8 |
|  | 79 | 21287.6 | 1681720 |  | 51 | 976.5 | 49801.5 | |  | | 51 | | 4559.5 | 232534.5 |
|  | 135 | 12390.7 | 1672745 |  | 56 | 796.1 | 44581.6 | |  | | 187 | | 212.5 | 39737.5 |
| **chr02** | 107 | 7242.4 | 774936.8 | **chr09** | 107 | 2259.5 | 241766.5 | | **chr16** | | 107 | | 35224.2 | 3768989 |
|  | 321 | 1410.1 | 452642.1 |  | 321 | 724.8 | 232660.8 | |  | | 79 | | 26308.8 | 2078395 |
|  | 214 | 1546.9 | 331036.6 |  | 214 | 766 | 163924 | |  | | 135 | | 15158 | 2046330 |
|  | 187 | 685.6 | 128207.2 |  | 377 | 159 | 59943 | |  | | 321 | | 6295 | 2020695 |
|  | 375 | 314.8 | 118050 |  | 66 | 903.4 | 59624.4 | |  | | 214 | | 7686.7 | 1644954 |
| **chr03** | 135 | 5505 | 743175 | **chr10** | 107 | 16792.9 | 1796840 | | **chr17** | | 107 | | 1415.4 | 151447.8 |
|  | 405 | 1813.6 | 734508 |  | 321 | 4059.8 | 1303196 | |  | | 428 | | 185.1 | 79222.8 |
|  | 270 | 771.8 | 208386 |  | 51 | 10911.9 | 556506.9 | |  | | 79 | | 987.8 | 78036.2 |
|  | 187 | 727.6 | 136061.2 |  | 79 | 6917.7 | 546498.3 | |  | | 185 | | 410.6 | 75961 |
|  | 268 | 363 | 97284 |  | 214 | 2376.1 | 508485.4 | |  | | 321 | | 189.2 | 60733.2 |
| **chr04** | 107 | 8281.1 | 886077.7 | **chr11** | 107 | 1531.9 | 163913.3 | | **chr18** | | 66 | | 3899.1 | 257,341 |
|  | 428 | 1690.8 | 723662.4 |  | 321 | 328.1 | 105320.1 | |  | | 68 | | 1109.4 | 75,439 |
|  | 214 | 2224 | 475936 |  | 383 | 67.2 | 25737.6 | |  | | 191 | | 381.2 | 72,809 |
|  | 429 | 356.8 | 153067.2 |  | 51 | 326.5 | 16651.5 | |  | | 382 | | 170.3 | 65,055 |
|  | 321 | 283.9 | 91131.9 |  | 187 | 73.9 | 13819.3 | |  | | 132 | | 459.7 | 60,680 |
| **chr05** | 107 | 7673.3 | 821043.1 | **chr12** | 107 | 1724.4 | 184510.8 | | **chr19** | | 321 | | 9751 | 3130071 |
|  | 214 | 1948.4 | 416957.6 |  | 66 | 1708.4 | 112754.4 | |  | | 107 | | 28732.5 | 3074378 |
|  | 321 | 1208.9 | 388056.9 |  | 428 | 223.3 | 95572.4 | |  | | 479 | | 5007.1 | 2398401 |
|  | 428 | 786.9 | 336793.2 |  | 51 | 1763.8 | 89953.8 | |  | | 214 | | 5318 | 1138052 |
|  | 372 | 513 | 190836 |  | 321 | 253.7 | 81437.7 | |  | | 51 | | 14678.6 | 748608.6 |
| **chr06** | 428 | 3873.4 | 1657815 | **chr13** | 371 | 1305.4 | 484303.4 |  | |  | |  | |  |
|  | 321 | 4375.9 | 1404664 |  | 107 | 4251.4 | 454899.8 |  | |  | |  | |  |
|  | 214 | 6207 | 1328298 |  | 264 | 1061.4 | 280209.6 |  | |  | |  | |  |
|  | 107 | 8931.6 | 955681.2 |  | 478 | 567.1 | 271073.8 |  | |  | |  | |  |
|  | 66 | 1780.5 | 117513 |  | 187 | 1070.3 | 200146.1 |  | |  | |  | |  |
| **chr07** | 107 | 8705.7 | 931509.9 | **chr14** | 187 | 382.2 | 71471.4 |  | |  | |  | |  |
|  | 428 | 1950.8 | 834942.4 |  | 56 | 1068.2 | 59819.2 |  | |  | |  | |  |
|  | 214 | 3566.4 | 763209.6 |  | 105 | 429.6 | 45108 |  | |  | |  | |  |
|  | 51 | 1517.2 | 77377.2 |  | 51 | 705.6 | 35985.6 |  | |  | |  | |  |
|  | 186 | 310.6 | 57771.6 |  | 107 | 328.3 | 35128.1 |  | |  | |  | |  |

**Table S7 GO enrichment analysis of genes on centromeres.**

| **Type** | **Term** | **Functions** |  | **Count** | **Percent** | **GeneRatio** | **PValue** | **Uniprot_ID** | **List Total** | **Pop Hits** | **Pop Total** | **Fold Enrichment** | **Bonferroni** | **Benjamini** | **FDR** |
| --- | --- | --- | --- | --- | --- | --- | --- | --- | --- | --- | --- | --- | --- | --- | --- |
| MF | GO:0005515 | protein binding | * | 12 | 16.21622 | 0.413793 | 0.037847 | C0LGN2, F4I171, Q8VXY4, Q01525, Q9ZSY8, Q9FWA3, O64884, Q9C865, P93033, B9DHT4, Q9M1S8, C0LGE0 | 29 | 4403 | 19266 | 1.81061502 | 0.794410411 | 0.935912437 | 0.935912437 |
| CC | GO:0005829 | cytosol | ** | 10 | 13.51351 | 0.333333 | 0.007555 | C0LGN2, Q94AH8, Q8VXY4, Q01525, Q9SRU2, Q9LNG5, Q9FWA3, P93033, Q9SD76, Q9M8R4 | 30 | 3286 | 26021 | 2.639582065 | 0.203490079 | 0.226655147 | 0.226655147 |
| CC | GO:0005739 | mitochondrion | * | 10 | 13.51351 | 0.333333 | 0.049614 | Q9SS83, P92555, Q01525, P92523, P93033, F4IM84, Q9M7J9, Q9FXA9, P92516, Q9SJK9 | 30 | 4483 | 26021 | 1.934790691 | 0.782727896 | 0.496137764 | 0.496137764 |
| CC | GO:0005737 | cytoplasm | . | 10 | 13.51351 | 0.333333 | 0.089247 | F4I171, Q8VXY4, Q01525, Q3E875, Q9FWA3, O64884, B9DHT4, Q9SD76, Q9M8R4, Q9FHM4 | 30 | 5007 | 26021 | 1.732308102 | 0.939464007 | 0.572805227 | 0.572805227 |
| CC | GO:0009506 | plasmodesma | . | 4 | 5.405405 | 0.133333 | 0.095468 | C0LGN2, Q8VZZ4, C0LGG7, Q9SRU2 | 30 | 985 | 26021 | 3.522301184 | 0.950713869 | 0.572805227 | 0.572805227 |
| CC | GO:0043231 | intracellular membrane-bounded organelle | * | 3 | 4.054054 | 0.1 | 0.03241 | Q9SS83, Q9FXA9, Q9SJK9 | 30 | 254 | 26021 | 10.24448819 | 0.62782831 | 0.486147555 | 0.486147555 |
| BP | GO:0009451 | RNA modification | ** | 3 | 4.054054 | 0.111111 | 0.005787 | Q9SS83, Q9FXA9, Q9SJK9 | 27 | 99 | 22556 | 25.31537598 | 0.417087805 | 0.532369009 | 0.532369009 |
| BP | GO:0046777 | protein autophosphorylation | * | 3 | 4.054054 | 0.111111 | 0.021699 | C0LGN2, C0LGG7, C0LGE0 | 27 | 198 | 22556 | 12.65768799 | 0.869999663 | 0.998151531 | 0.998151531 |
| BP | GO:0015074 | DNA integration | . | 2 | 2.702703 | 0.074074 | 0.057186 | Q94HW2, Q9ZT94 | 27 | 51 | 22556 | 32.7610748 | 0.995815725 | 1 | 1 |
| BP | GO:0006310 | DNA recombination | . | 2 | 2.702703 | 0.074074 | 0.073398 | Q94HW2, Q9ZT94 | 27 | 66 | 22556 | 25.31537598 | 0.999166184 | 1 | 1 |
| BP | GO:0009640 | photomorphogenesis |  | 2 | 2.702703 | 0.074074 | 0.077676 | Q9SRU2, Q9M1S8 | 27 | 70 | 22556 | 23.86878307 | 0.999457777 | 1 | 1 |

**The list of genes predicted on centromeres.**

| **Chr** | **Gene** | **Start_Pos** | **End_Pos** | **Forward_Reverse** | **Gene_ID** | **Swissprot_ID** | **Identity** | **Gene annotation** |
| --- | --- | --- | --- | --- | --- | --- | --- | --- |
| Chr01 | gene | 15168189 | 15168518 | + | **Vitis01g01292** | |  |  |
| Chr01 | gene | 15181763 | 15182659 | + | **Vitis01g01293** | P10978 | 48.6 | Retrovirus-related Pol polyprotein from transposon TNT 1-94 (POLX_TOBAC) |
| Chr01 | gene | 15189666 | 15204331 | + | Vitis01g01294 | Q852M4 | 65.3 | probable glutamate carboxypeptidase PLA3(LOC4334380) |
| Chr01 | gene | 15254680 | 15254952 | + | Vitis01g01295 |  |  |  |
| Chr01 | gene | 15256147 | 15256452 | + | **Vitis01g01296** | |  |  |
| Chr01 | gene | 15293428 | 15293974 | - | Vitis01g01297 | O81160 | 66.7 | Arginine decarboxylase(SPE2) |
| Chr01 | gene | 15370082 | 15382049 | - | Vitis01g01298 | Q9M1S8 | 62.2 | Peptidase M28 family protein(AMP1) |
| Chr01 | gene | 15384369 | 15416280 | - | Vitis01g01299 | Q3E875 | 83.8 | small G protein family protein / RhoGAP family protein(AT5G61530) |
| Chr01 | gene | 15414452 | 15414712 | - | Vitis01g01300 |  |  |  |
| Chr01 | gene | 15416344 | 15416667 | - | Vitis01g01301 |  |  |  |
| Chr02 | gene | 12953156 | 12953620 | + | **Vitis02g01118** | P10978 | 47.7 | Retrovirus-related Pol polyprotein from transposon TNT 1-94 (POLX_TOBAC) |
| Chr02 | gene | 12966041 | 12991379 | + | Vitis02g01119 |  |  |  |
| Chr02 | gene | 12994982 | 12995296 | + | Vitis02g01120 | P92555 | 50.7 | Uncharacterized mitochondrial protein AtMg01250(AtMg01250) |
| Chr02 | gene | 12997646 | 12998410 | - | Vitis02g01121 | P04146 | 35.6 | GIP-like(Gip) |
| Chr02 | gene | 12998786 | 13001972 | - | Vitis02g01122 | P10978 | 47.8 | Retrovirus-related Pol polyprotein from transposon TNT 1-94 (POLX_TOBAC) |
| Chr02 | gene | 13005005 | 13005217 | + | Vitis02g01123 | Q9FWA3 | 75.7 | 6-phosphogluconate dehydrogenase family protein(AT3G02360) |
| Chr02 | gene | 13009497 | 13019125 | + | Vitis02g01124 | P04323 | 30.1 | Retrovirus-related Pol polyprotein from transposon 17.6(pol) |
| Chr02 | gene | 13022676 | 13023129 | + | Vitis02g01125 | Q9LI00 | 72.3 | 6-phosphogluconate dehydrogenase, decarboxylating 1-like(LOC4339892) |
| Chr02 | gene | 13026412 | 13026939 | - | Vitis02g01126 | P04323 | 41.5 | Retrovirus-related Pol polyprotein from transposon 17.6(pol) |
| Chr02 | gene | 13039883 | 13040173 | + | Vitis02g01127 | Q9FHM4 | 60 | Microtubule associated protein (MAP65/ASE1) family protein(PLE) |
| Chr02 | gene | 13041977 | 13042267 | + | Vitis02g01128 | P93033 | 67.4 | fumarase 1(FUM1) |
| Chr02 | gene | 13045729 | 13054868 | - | Vitis02g01129 | Q6NU40 | 31 | chromosome transmission fidelity factor 18 L homeolog(chtf18.L) |
| Chr02 | gene | 13062074 | 13072625 | + | Vitis02g01130 | B9DHT4 | 55.6 | ARM repeat protein interacting with ABF2(ARIA) |
| Chr02 | gene | 13112671 | 13113378 | - | Vitis02g01131 | P0CT34 | 38.8 | retrotransposable element(Tf2-1) |
| Chr02 | gene | 13114521 | 13115162 | - | Vitis02g01132 |  |  |  |
| Chr02 | gene | 13130093 | 13130347 | - | Vitis02g01133 | Q0G9J5 | 91.3 | ribosomal protein S12(rps12) |
| Chr02 | gene | 13132809 | 13133255 | - | Vitis02g01134 | P04323 | 41.9 | Retrovirus-related Pol polyprotein from transposon 17.6(pol) |
| Chr02 | gene | 13181944 | 13189276 | - | Vitis02g01135 | B9DHT4 | 40.5 | ARM repeat protein interacting with ABF2(ARIA) |
| Chr02 | gene | 13211093 | 13222351 | + | Vitis02g01136 | Q6NU40 | 29.7 | chromosome transmission fidelity factor 18 L homeolog(chtf18.L) |
| Chr02 | gene | 13230741 | 13231019 | - | Vitis02g01137 | P92555 | 54.3 | Uncharacterized mitochondrial protein AtMg01250(AtMg01250) |
| Chr02 | gene | 13234164 | 13236007 | - | Vitis02g01138 |  |  |  |
| Chr02 | gene | 13270280 | 13270864 | - | Vitis02g01139 | P14381 | 30.3 | Transposon TX1 uncharacterized 149 kDa protein (YTX2_XENLA) |
| Chr02 | gene | 13277616 | 13282434 | + | Vitis02g01140 |  |  |  |
| Chr02 | gene | 13359187 | 13359885 | + | Vitis02g01141 | Q9SRU2 | 56.8 | auxin transport protein (BIG)(BIG) |
| Chr04 | gene | 13299698 | 13299943 | + | Vitis04g01046 |  |  |  |
| Chr04 | gene | 13323316 | 13330465 | + | Vitis04g01047 | C0LGE0 | 41.7 | Leucine-rich repeat transmembrane protein kinase(AT1G07650) |
| Chr04 | gene | 13409974 | 13427842 | - | Vitis04g01048 | B9DHT4 | 66 | ARM repeat protein interacting with ABF2(ARIA) |
| Chr04 | gene | 13443238 | 13454575 | + | Vitis04g01049 |  |  |  |
| Chr04 | gene | 13462434 | 13463971 | - | Vitis04g01050 |  |  |  |
| Chr04 | gene | 13482940 | 13483515 | - | **Vitis04g01051** | |  |  |
| Chr05 | gene | 13975095 | 13975769 | + | **Vitis05g01185** | |  |  |
| Chr05 | gene | 13978330 | 13978968 | + | **Vitis05g01186** | |  |  |
| Chr05 | gene | 13979038 | 13979988 | + | **Vitis05g01187** | Q7LHG5 | 35.8 |  |
| Chr05 | gene | 13992125 | 13994279 | + | **Vitis05g01188** | P04323 | 42.1 | Retrovirus-related Pol polyprotein from transposon 17.6(pol) |
| Chr05 | gene | 13993094 | 13994086 | + | **Vitis05g01189** | P03371 | 30.5 | Pol polyprotein(pol) |
| Chr05 | gene | 14020563 | 14021027 | + | **Vitis05g01190** | P10978 | 48.4 | Retrovirus-related Pol polyprotein from transposon TNT 1-94 (POLX_TOBAC) |
| Chr05 | gene | 14036607 | 14038388 | + | Vitis05g01191 | P14381 | 32.7 | Transposon TX1 uncharacterized 149 kDa protein (YTX2_XENLA) |
| Chr05 | gene | 14060801 | 14063148 | + | **Vitis05g01192** | |  |  |
| Chr05 | gene | 14064013 | 14066722 | + | **Vitis05g01193** | |  |  |
| Chr05 | gene | 14079444 | 14079716 | - | Vitis05g01194 |  |  |  |
| Chr05 | gene | 14083917 | 14084267 | + | Vitis05g01195 |  |  |  |
| Chr05 | gene | 14105410 | 14116177 | + | Vitis05g01196 | C0LGG7 | 36.6 | Leucine-rich repeat transmembrane protein kinase(AT1G53420) |
| Chr05 | gene | 14155854 | 14163993 | + | Vitis05g01197 |  |  |  |
| Chr05 | gene | 14167682 | 14168137 | + | Vitis05g01198 | A6MM82 | 91.6 | ribosomal protein S7(rps7) |
| Chr05 | gene | 14204156 | 14205589 | - | **Vitis05g01199** | Q94HW2 | 42.6 | Retrovirus-related Pol polyprotein from transposon RE1(RE1) |
| Chr05 | gene | 14209855 | 14210355 | + | Vitis05g01200 | Q9FXA9 | 40.9 | Tetratricopeptide repeat (TPR)-like superfamily protein(PGN) |
| Chr05 | gene | 14224712 | 14225165 | + | Vitis05g01201 | F4I171 | 67.7 | mediator of RNA polymerase II transcription subunit 15a-like protein(NRB4) |
| Chr05 | gene | 14232634 | 14233491 | + | **Vitis05g01202** | P04323 | 38.8 | Retrovirus-related Pol polyprotein from transposon 17.6(pol) |
| Chr05 | gene | 14239219 | 14239521 | + | **Vitis05g01203** | Q9SS83 | 43.6 | Pentatricopeptide repeat (PPR) superfamily protein(AT3G09040) |
| Chr05 | gene | 14240173 | 14241753 | + | **Vitis05g01204** | Q01899 | 62.9 | Heat shock 70 kDa protein, mitochondrial (HSP7M_PHAVU) |
| Chr05 | gene | 14308183 | 14335763 | - | Vitis05g01205 | C0LGN2 | 55.4 | Leucine-rich repeat transmembrane protein kinase(AT3G14840) |
| Chr05 | gene | 14357117 | 14357584 | + | **Vitis05g01206** | |  |  |
| Chr05 | gene | 14582260 | 14583925 | - | **Vitis05g01207** | |  |  |
| Chr06 | gene | 10623865 | 10628692 | - | Vitis06g01022 |  |  |  |
| Chr06 | gene | 10634969 | 10636771 | + | Vitis06g01023 | P14381 | 28.9 | Transposon TX1 uncharacterized 149 kDa protein (YTX2_XENLA) |
| Chr06 | gene | 10660218 | 10660553 | + | Vitis06g01024 | P92555 | 52.9 | Uncharacterized mitochondrial protein AtMg01250(AtMg01250) |
| Chr06 | gene | 10683609 | 10689883 | + | Vitis06g01025 | A5B4D2 | 94.6 | translation factor GUF1 homolog, chloroplastic(LOC100252626) |
| Chr06 | gene | 10693524 | 10699959 | - | Vitis06g01026 |  |  |  |
| Chr06 | gene | 10693879 | 10694899 | - | Vitis06g01027 |  |  |  |
| Chr06 | gene | 10715251 | 10730726 | + | Vitis06g01028 | C0LGG7 | 35.8 | Leucine-rich repeat transmembrane protein kinase(AT1G53420) |
| Chr06 | gene | 10783743 | 10783943 | + | Vitis06g01029 | A4GYN8 | 92.4 | ribosomal protein S12(rps12) |
| Chr06 | gene | 10802451 | 10802886 | + | **Vitis06g01030** | F4I171 | 69.4 | mediator of RNA polymerase II transcription subunit 15a-like protein(NRB4) |
| Chr06 | gene | 10811133 | 10813970 | - | Vitis06g01031 | Q9SJK9 | 47.5 | Tetratricopeptide repeat (TPR)-like superfamily protein(AT2G36980) |
| Chr06 | gene | 10817082 | 10817807 | + | Vitis06g01032 |  |  |  |
| Chr06 | gene | 10829330 | 10830691 | + | Vitis06g01033 | Q7LHG5 | 34.9 |  |
| Chr06 | gene | 10905946 | 10911705 | - | Vitis06g01034 | Q53P98 | 48.8 | signal peptide peptidase-like 2(LOC4350402) |
| Chr06 | gene | 10913710 | 10957699 | + | Vitis06g01035 | Q6NU40 | 26.7 | chromosome transmission fidelity factor 18 L homeolog(chtf18.L) |
| Chr06 | gene | 10934410 | 10936138 | - | Vitis06g01036 | Q94HW2 | 45.8 | Retrovirus-related Pol polyprotein from transposon RE1(RE1) |
| Chr06 | gene | 10962133 | 10962546 | - | **Vitis06g01037** | P10978 | 44.9 | Retrovirus-related Pol polyprotein from transposon TNT 1-94 (POLX_TOBAC) |
| Chr07 | gene | 12589514 | 12590503 | - | **Vitis07g01066** | |  |  |
| Chr07 | gene | 12590570 | 12591553 | - | **Vitis07g01067** | P04323 | 42.6 | Retrovirus-related Pol polyprotein from transposon 17.6(pol) |
| Chr07 | gene | 13466472 | 13466807 | - | Vitis07g01068 | Q9CPD5 | 35.5 | YggS family pyridoxal phosphate-dependent enzyme(PM_RS00575) |
| Chr07 | gene | 13477053 | 13477688 | + | Vitis07g01069 |  |  |  |
| Chr07 | gene | 13477789 | 13478535 | + | Vitis07g01070 |  |  |  |
| Chr07 | gene | 13481046 | 13482152 | + | **Vitis07g01071** | |  |  |
| Chr07 | gene | 13484875 | 13494860 | + | **Vitis07g01072** | P04323 | 38.8 | Retrovirus-related Pol polyprotein from transposon 17.6(pol) |
| Chr07 | gene | 13505774 | 13507552 | + | **Vitis07g01073** | |  |  |
| Chr07 | gene | 13508104 | 13508808 | + | Vitis07g01074 | Q9LNG5 | 47.9 | serine/threonine-protein phosphatase 7 long form-like protein(AT1G48120) |
| Chr07 | gene | 13513644 | 13514333 | + | **Vitis07g01075** | Q9M7J9 | 43.2 | Potassium transporter family protein(AT5G14880) |
| Chr07 | gene | 13517170 | 13517493 | + | Vitis07g01076 |  |  |  |
| Chr07 | gene | 13517583 | 13517816 | + | Vitis07g01077 | Q8LQJ8 | 72.7 | ATP-dependent zinc metalloprotease FTSH 5, mitochondrial-like(LOC4326311) |
| Chr07 | gene | 13570401 | 13572731 | - | Vitis07g01078 | Q7LHG5 | 40.9 |  |
| Chr07 | gene | 13574715 | 13575251 | - | **Vitis07g01079** | |  |  |
| Chr07 | gene | 13591826 | 13592230 | + | Vitis07g01080 |  |  |  |
| Chr07 | gene | 13597089 | 13597881 | + | Vitis07g01081 |  |  |  |
| Chr07 | gene | 13597912 | 13598610 | + | Vitis07g01082 | Q7LHG5 | 45 |  |
| Chr07 | gene | 13613406 | 13625571 | + | Vitis07g01083 |  |  |  |
| Chr07 | gene | 13724013 | 13727022 | - | Vitis07g01084 | Q71VM4 | 86.2 | importin subunit alpha-1a-like(LOC4327117) |
| Chr07 | gene | 13724650 | 13724910 | - | Vitis07g01085 | Q8VXY4 | 69 | TIP41-like family protein(AT4G34270) |
| Chr07 | gene | 13746237 | 13746536 | - | **Vitis07g01086** | |  |  |
| Chr07 | gene | 13772875 | 13773393 | + | **Vitis07g01087** | P03371 | 29.5 | Pol polyprotein(pol) |
| Chr07 | gene | 13782527 | 13783342 | + | **Vitis07g01088** | |  |  |
| Chr07 | gene | 13794035 | 13795257 | - | **Vitis07g01089** | |  |  |
| Chr08 | gene | 6736291 | 6738405 | - | **Vitis08g00429** | Q7LHG5 | 28.5 |  |
| Chr08 | gene | 6768087 | 6768794 | - | **Vitis08g00430** | |  |  |
| Chr08 | gene | 6769645 | 6771393 | - | **Vitis08g00431** | Q8I7P9 | 35.4 | Retrovirus-related Pol polyprotein from transposon opus(pol) |
| Chr08 | gene | 6772463 | 6773677 | - | **Vitis08g00432** | |  |  |
| Chr08 | gene | 6815384 | 6836923 | + | Vitis08g00433 | Q9C865 | 68.8 | SH3 domain-containing protein(AT1G31440) |
| Chr08 | gene | 6870986 | 6872764 | - | Vitis08g00434 | Q7LHG5 | 34.7 |  |
| Chr08 | gene | 6988492 | 6988818 | - | **Vitis08g00435** | |  |  |
| Chr08 | gene | 7020765 | 7022516 | - | Vitis08g00436 |  |  |  |
| Chr08 | gene | 7038305 | 7039744 | + | Vitis08g00437 | P04323 | 37.9 | Retrovirus-related Pol polyprotein from transposon 17.6(pol) |
| Chr08 | gene | 7039865 | 7041280 | - | Vitis08g00438 | P0CT42 | 31 | retrotransposable element(Tf2-7) |
| Chr08 | gene | 7039865 | 7041430 | - | Vitis08g00439 | Q7LHG5 | 33.7 |  |
| Chr08 | gene | 7046340 | 7046855 | + | **Vitis08g00440** | |  |  |
| Chr08 | gene | 7046397 | 7046855 | + | **Vitis08g00441** | |  |  |
| Chr08 | gene | 7049369 | 7049755 | + | **Vitis08g00442** | |  |  |
| Chr08 | gene | 7061074 | 7062066 | - | **Vitis08g00443** | |  |  |
| Chr08 | gene | 7061074 | 7070173 | - | **Vitis08g00444** | |  |  |
| Chr09 | gene | 15103689 | 15104367 | + | **Vitis09g01146** | Q9SD76 | 76 | alpha-glucan phosphorylase 2(PHS2) |
| Chr09 | gene | 15125264 | 15125629 | + | **Vitis09g01147** | |  |  |
| Chr09 | gene | 15125697 | 15126110 | + | **Vitis09g01148** | P10978 | 45.7 | Retrovirus-related Pol polyprotein from transposon TNT 1-94 (POLX_TOBAC) |
| Chr09 | gene | 15130556 | 15162466 | - | Vitis09g01149 | Q6NU40 | 25 | chromosome transmission fidelity factor 18 L homeolog(chtf18.L) |
| Chr09 | gene | 15162530 | 15163831 | + | Vitis09g01150 |  |  |  |
| Chr09 | gene | 15167532 | 15167867 | + | Vitis09g01151 | P92555 | 52.9 | Uncharacterized mitochondrial protein AtMg01250(AtMg01250) |
| Chr09 | gene | 15178935 | 15187613 | - | Vitis09g01152 | Q6NU40 | 29.7 | chromosome transmission fidelity factor 18 L homeolog(chtf18.L) |
| Chr09 | gene | 15200531 | 15221483 | + | Vitis09g01153 | C0LGG7 | 39.2 | Leucine-rich repeat transmembrane protein kinase(AT1G53420) |
| Chr09 | gene | 15224590 | 15224856 | + | Vitis09g01154 |  |  |  |
| Chr09 | gene | 15250453 | 15250707 | + | Vitis09g01155 | Q0G9J5 | 95 | ribosomal protein S12(rps12) |
| Chr09 | gene | 15251300 | 15251764 | + | Vitis09g01156 | Q67ID1 | 96 | 30S ribosomal protein S7, chloroplastic(rps7) |
| Chr09 | gene | 15251486 | 15267716 | + | Vitis09g01157 |  |  |  |
| Chr09 | gene | 15251486 | 15267716 | + | Vitis09g01158 | P10978 | 34.2 | Retrovirus-related Pol polyprotein from transposon TNT 1-94 (POLX_TOBAC) |
| Chr09 | gene | 15251486 | 15276935 | + | Vitis09g01159 | F4I171 | 69.4 | mediator of RNA polymerase II transcription subunit 15a-like protein(NRB4) |
| Chr09 | gene | 15282082 | 15282697 | - | Vitis09g01160 | Q01899 | 64.8 | Heat shock 70 kDa protein, mitochondrial (HSP7M_PHAVU) |
| Chr09 | gene | 15305376 | 15315452 | + | Vitis09g01161 | F4I171 | 71 | mediator of RNA polymerase II transcription subunit 15a-like protein(NRB4) |
| Chr09 | gene | 15305385 | 15305827 | + | Vitis09g01162 | F4I171 | 71 | mediator of RNA polymerase II transcription subunit 15a-like protein(NRB4) |
| Chr09 | gene | 15349696 | 15362550 | - | Vitis09g01163 | B9DHT4 | 48.8 | ARM repeat protein interacting with ABF2(ARIA) |
| Chr09 | gene | 15399230 | 15400858 | + | Vitis09g01164 |  |  |  |
| Chr09 | gene | 15418939 | 15419337 | - | Vitis09g01165 | P92555 | 54.4 | Uncharacterized mitochondrial protein AtMg01250(AtMg01250) |
| Chr09 | gene | 15422452 | 15422733 | - | Vitis09g01166 |  |  |  |
| Chr09 | gene | 15422874 | 15445099 | - | Vitis09g01167 |  |  |  |
| Chr11 | gene | 13557593 | 13558300 | - | Vitis11g01130 |  |  |  |
| Chr11 | gene | 13564661 | 13577539 | - | Vitis11g01131 | Q54RF3 | 43.2 | bacterial transferase hexapeptide repeat-containing protein(eIF2b5) |
| Chr11 | gene | 13565483 | 13566065 | - | Vitis11g01132 |  |  |  |
| Chr11 | gene | 13566071 | 13567235 | - | Vitis11g01133 | P92555 | 46.4 | Uncharacterized mitochondrial protein AtMg01250(AtMg01250) |
| Chr11 | gene | 13597874 | 13598330 | - | Vitis11g01134 |  |  |  |
| Chr11 | gene | 13608405 | 13610440 | + | Vitis11g01135 |  |  |  |
| Chr11 | gene | 13612904 | 13613639 | - | Vitis11g01136 | Q9ZV51 | 48.1 | RING/U-box superfamily protein(AT2G18670) |
| Chr11 | gene | 13629739 | 13630251 | + | **Vitis11g01137** | |  |  |
| Chr12 | gene | 11442661 | 11442750 | + | Vitis12g01046 | Q0G9J5 | 82.1 | ribosomal protein S12(rps12) |
| Chr12 | gene | 11442753 | 11442911 | + | Vitis12g01047 | Q0G9J5 | 91.8 | ribosomal protein S12(rps12) |
| Chr12 | gene | 11443499 | 11443967 | + | Vitis12g01048 | B1NWJ5 | 93.3 | ribosomal protein S7(rps7) |
| Chr13 | gene | 12022279 | 12023445 | + | **Vitis13g01017** | F9VN79 | 36.4 | ribonuclease HI family protein(STK_RS04185) |
| Chr13 | gene | 12052800 | 12055265 | - | **Vitis13g01018** | |  |  |
| Chr13 | gene | 12079383 | 12080675 | - | **Vitis13g01019** | P0CT42 | 30.3 | retrotransposable element(Tf2-7) |
| Chr13 | gene | 12085712 | 12087682 | + | **Vitis13g01020** | P04323 | 41.6 | Retrovirus-related Pol polyprotein from transposon 17.6(pol) |
| Chr13 | gene | 12119684 | 12130745 | - | Vitis13g01021 | Q9M1S8 | 65.9 | Peptidase M28 family protein(AMP1) |
| Chr13 | gene | 12147272 | 12147754 | - | **Vitis13g01022** | P10978 | 47.1 | Retrovirus-related Pol polyprotein from transposon TNT 1-94 (POLX_TOBAC) |
| Chr13 | gene | 12148825 | 12149571 | - | **Vitis13g01023** | |  |  |
| Chr13 | gene | 12157311 | 12157643 | + | Vitis13g01024 |  |  |  |
| Chr14 | gene | 15149111 | 15149434 | + | **Vitis14g01187** | |  |  |
| Chr14 | gene | 15172733 | 15173032 | + | **Vitis14g01188** | |  |  |
| Chr14 | gene | 15207023 | 15210113 | + | Vitis14g01189 | Q71VM4 | 85.1 | importin subunit alpha-1a-like(LOC4327117) |
| Chr14 | gene | 15401559 | 15401990 | - | Vitis14g01190 | F4IM84 | 48.2 | DEA(D/H)-box RNA helicase family protein(AT2G01130) |
| Chr14 | gene | 15468262 | 15469045 | + | Vitis14g01191 | P02553 | 53.6 | Tubulin alpha chain (TBA_LYTPI) |
| Chr14 | gene | 15468695 | 15472769 | + | Vitis14g01192 | P02553 | 53.6 | Tubulin alpha chain (TBA_LYTPI) |
| Chr15 | gene | 7188149 | 7196065 | + | **Vitis15g00357** | |  |  |
| Chr15 | gene | 7245876 | 7248716 | - | **Vitis15g00358** | |  |  |
| Chr15 | gene | 7263705 | 7267004 | - | **Vitis15g00359** | |  |  |
| Chr15 | gene | 7347498 | 7350191 | - | **Vitis15g00360** | |  |  |
| Chr15 | gene | 7366822 | 7369352 | - | **Vitis15g00361** | |  |  |
| Chr15 | gene | 7381531 | 7392344 | - | **Vitis15g00362** | |  |  |
| Chr15 | gene | 7394022 | 7396285 | - | **Vitis15g00363** | |  |  |
| Chr15 | gene | 7412350 | 7413977 | - | **Vitis15g00364** | |  |  |
| Chr15 | gene | 7513605 | 7515230 | - | **Vitis15g00365** | |  |  |
| Chr15 | gene | 7651502 | 7658911 | - | **Vitis15g00366** | |  |  |
| Chr16 | gene | 9294491 | 9508218 | - | **Vitis16g00638** | |  |  |
| Chr17 | gene | 15233084 | 15246292 | + | Vitis17g01306 | Q9M8R4 | 59.2 | Class I glutamine amidotransferase-like superfamily protein(DJ1D) |
| Chr17 | gene | 15251297 | 15251828 | + | Vitis17g01307 | Q03033 | 44 | elongation factor 1-alpha-like(LOC543386) |
| Chr17 | gene | 15254762 | 15256346 | - | Vitis17g01308 | Q94HW2 | 46.8 | Retrovirus-related Pol polyprotein from transposon RE1(RE1) |
| Chr17 | gene | 15256552 | 15256848 | + | **Vitis17g01309** | |  |  |
| Chr17 | gene | 15275386 | 15278521 | + | Vitis17g01310 | Q71VM4 | 84.6 | importin subunit alpha-1a-like(LOC4327117) |
| Chr17 | gene | 15277330 | 15277710 | + | Vitis17g01311 | Q8VXY4 | 69 | TIP41-like family protein(AT4G34270) |
| Chr17 | gene | 15278094 | 15278521 | + | Vitis17g01312 | Q71VM4 | 80.6 | importin subunit alpha-1a-like(LOC4327117) |
| Chr17 | gene | 15278562 | 15278870 | + | **Vitis17g01313** | Q9ZSY8 | 69.3 | phytochrome-associated protein 2(PAP2) |
| Chr17 | gene | 15307219 | 15308005 | + | **Vitis17g01314** | |  |  |
| Chr17 | gene | 15311398 | 15311760 | + | **Vitis17g01315** | P31843 | 49.2 | RNA-directed DNA polymerase homolog (RRPO_OENBE) |
| Chr17 | gene | 15341974 | 15342804 | + | **Vitis17g01316** | P31843 | 56.5 | RNA-directed DNA polymerase homolog (RRPO_OENBE) |
| Chr17 | gene | 15343124 | 15344551 | + | **Vitis17g01317** | Q7LHG5 | 35 |  |
| Chr17 | gene | 15373842 | 15375806 | + | **Vitis17g01318** | |  |  |
| Chr17 | gene | 15405113 | 15405718 | + | **Vitis17g01319** | P04323 | 40.4 | Retrovirus-related Pol polyprotein from transposon 17.6(pol) |
| Chr17 | gene | 15422517 | 15429637 | + | Vitis17g01320 | P10978 | 35.1 | Retrovirus-related Pol polyprotein from transposon TNT 1-94 (POLX_TOBAC) |
| Chr17 | gene | 15427397 | 15428232 | + | **Vitis17g01321** | |  |  |
| Chr17 | gene | 15430696 | 15431019 | + | **Vitis17g01322** | P0CV72 | 56.1 | Secreted RxLR effector protein 161(RXLR161) |
| Chr17 | gene | 15497477 | 15501422 | - | Vitis17g01323 | Q71VM4 | 83.8 | importin subunit alpha-1a-like(LOC4327117) |
| Chr17 | gene | 15536401 | 15538961 | - | Vitis17g01324 | Q7LHG5 | 29 |  |
| Chr17 | gene | 15541908 | 15542252 | + | Vitis17g01325 | P18601 | 42.4 | Actin, clone 211 (ACT2_ARTSX) |
| Chr17 | gene | 15542337 | 15548282 | + | Vitis17g01326 | O64884 | 71.4 | O-fucosyltransferase family protein(AT2G44500) |
| Chr17 | gene | 15549422 | 15550537 | - | Vitis17g01327 | Q9ZT94 | 56.3 | pseudo(RE2) |
| Chr17 | gene | 15551494 | 15552921 | - | **Vitis17g01328** | Q9ZT94 | 45.4 | pseudo(RE2) |
| Chr17 | gene | 15552991 | 15553278 | - | **Vitis17g01329** | |  |  |
| Chr17 | gene | 15554238 | 15554543 | + | Vitis17g01330 | Q94AH8 | 56.7 | UDP-Glycosyltransferase / trehalose-phosphatase family protein(ATTPS6) |
| Chr18 | gene | 15055195 | 15055536 | + | **Vitis18g01489** | |  |  |
| Chr18 | gene | 15056907 | 15057164 | - | **Vitis18g01490** | |  |  |
| Chr18 | gene | 15068951 | 15069247 | + | **Vitis18g01491** | P04323 | 46.3 | Retrovirus-related Pol polyprotein from transposon 17.6(pol) |
| Chr18 | gene | 15078236 | 15078811 | + | **Vitis18g01492** | |  |  |
| Chr18 | gene | 15078857 | 15079648 | + | **Vitis18g01493** | |  |  |
| Chr18 | gene | 15115179 | 15115604 | - | **Vitis18g01494** | |  |  |
| Chr18 | gene | 15119044 | 15119229 | - | **Vitis18g01495** | P31843 | 63.6 | RNA-directed DNA polymerase homolog (RRPO_OENBE) |
| Chr18 | gene | 15152580 | 15153287 | + | Vitis18g01496 |  |  |  |
| Chr18 | gene | 15167780 | 15171280 | + | Vitis18g01497 | P04323 | 36.7 | Retrovirus-related Pol polyprotein from transposon 17.6(pol) |
| Chr18 | gene | 15173494 | 15174162 | + | Vitis18g01498 |  |  |  |
| Chr18 | gene | 15178476 | 15179138 | + | Vitis18g01499 | P04323 | 48.7 | Retrovirus-related Pol polyprotein from transposon 17.6(pol) |
| Chr18 | gene | 15179205 | 15179534 | + | Vitis18g01500 |  |  |  |
| Chr18 | gene | 15179205 | 15179534 | + | Vitis18g01501 |  |  |  |
| Chr18 | gene | 15179631 | 15180197 | + | Vitis18g01502 |  |  |  |
| Chr18 | gene | 15214667 | 15215092 | - | **Vitis18g01503** | |  |  |
| Chr18 | gene | 15218480 | 15218827 | - | **Vitis18g01504** | P31843 | 67.9 | RNA-directed DNA polymerase homolog (RRPO_OENBE) |
| Chr18 | gene | 15242319 | 15242744 | + | Vitis18g01505 | Q8I7P9 | 36.1 | Retrovirus-related Pol polyprotein from transposon opus(pol) |
| Chr18 | gene | 15246273 | 15246596 | + | **Vitis18g01506** | |  |  |
| Chr18 | gene | 15278831 | 15279592 | - | **Vitis18g01507** | |  |  |
| Chr18 | gene | 15279839 | 15280567 | - | **Vitis18g01508** | |  |  |
| Chr18 | gene | 15308703 | 15309704 | + | **Vitis18g01509** | |  |  |
| Chr18 | gene | 15340506 | 15344632 | - | **Vitis18g01510** | |  |  |
| Chr18 | gene | 15344111 | 15344632 | - | **Vitis18g01511** | |  |  |
| Chr18 | gene | 15344636 | 15345832 | - | **Vitis18g01512** | |  |  |
| Chr18 | gene | 15356597 | 15358084 | - | **Vitis18g01513** | |  |  |
| Chr18 | gene | 15395156 | 15395623 | + | **Vitis18g01514** | |  |  |
| Chr18 | gene | 15395711 | 15398794 | + | **Vitis18g01515** | P04323 | 37.4 | Retrovirus-related Pol polyprotein from transposon 17.6(pol) |
| Chr18 | gene | 15397373 | 15398794 | + | **Vitis18g01516** | P04323 | 37.4 | Retrovirus-related Pol polyprotein from transposon 17.6(pol) |
| Chr18 | gene | 15398852 | 15400564 | + | **Vitis18g01517** | |  |  |
| Chr18 | gene | 15400817 | 15401230 | - | **Vitis18g01518** | |  |  |
| Chr18 | gene | 15401419 | 15401712 | + | **Vitis18g01519** | |  |  |
| Chr18 | gene | 15416034 | 15416471 | + | **Vitis18g01520** | |  |  |
| Chr18 | gene | 15427939 | 15428316 | - | **Vitis18g01521** | P92516 | 64.9 | Uncharacterized mitochondrial protein AtMg00750(AtMg00750) |
| Chr18 | gene | 15451707 | 15452309 | - | **Vitis18g01522** | Q8I7P9 | 36.8 | Retrovirus-related Pol polyprotein from transposon opus(pol) |
| Chr18 | gene | 15452382 | 15454478 | - | **Vitis18g01523** | P04323 | 37.8 | Retrovirus-related Pol polyprotein from transposon 17.6(pol) |
| Chr18 | gene | 15491215 | 15491526 | - | **Vitis18g01524** | |  |  |
| Chr18 | gene | 15507476 | 15507883 | - | **Vitis18g01525** | |  |  |
| Chr18 | gene | 15507941 | 15508195 | - | **Vitis18g01526** | |  |  |
| Chr18 | gene | 15540719 | 15540964 | - | **Vitis18g01527** | |  |  |
| Chr18 | gene | 15541288 | 15541590 | - | **Vitis18g01528** | |  |  |
| Chr18 | gene | 15541778 | 15543438 | + | **Vitis18g01529** | |  |  |
| Chr18 | gene | 15579768 | 15580764 | - | Vitis18g01530 |  |  |  |
| Chr18 | gene | 15583729 | 15586806 | + | Vitis18g01531 | P20825 | 35.6 | Retrovirus-related Pol polyprotein from transposon 297(pol) |
| Chr18 | gene | 15600414 | 15602135 | + | Vitis18g01532 | Q8I7P9 | 39.3 | Retrovirus-related Pol polyprotein from transposon opus(pol) |
| Chr18 | gene | 15601143 | 15602135 | + | Vitis18g01533 |  |  |  |
| Chr18 | gene | 15608514 | 15631457 | - | Vitis18g01534 | P04323 | 36.2 | Retrovirus-related Pol polyprotein from transposon 17.6(pol) |
| Chr18 | gene | 15610534 | 15610911 | - | **Vitis18g01535** | |  |  |
| Chr18 | gene | 15655449 | 15655874 | - | **Vitis18g01536** | P03371 | 27.4 | Pol polyprotein(pol) |
| Chr18 | gene | 15655995 | 15657053 | - | **Vitis18g01537** | Q8I7P9 | 29.3 | Retrovirus-related Pol polyprotein from transposon opus(pol) |
| Chr18 | gene | 15670480 | 15671235 | - | Vitis18g01538 | P05400 | 30.5 | Enzymatic polyprotein [Contains: Aspartic protease; Endonuclease; Reverse transcriptase](CERVgp5) |
| Chr18 | gene | 15692644 | 15693393 | - | **Vitis18g01539** | P32542 | 34.4 | Pol polyprotein(pol) |
| Chr18 | gene | 15731445 | 15732062 | - | **Vitis18g01540** | P03371 | 29.5 | Pol polyprotein(pol) |
| Chr18 | gene | 15733979 | 15734755 | - | **Vitis18g01541** | P04323 | 40.8 | Retrovirus-related Pol polyprotein from transposon 17.6(pol) |
| Chr18 | gene | 15795339 | 15797868 | - | **Vitis18g01542** | P04323 | 37.2 | Retrovirus-related Pol polyprotein from transposon 17.6(pol) |
| Chr18 | gene | 15799602 | 15802118 | - | **Vitis18g01543** | |  |  |
| Chr18 | gene | 15847461 | 15849566 | + | **Vitis18g01544** | P04323 | 36.7 | Retrovirus-related Pol polyprotein from transposon 17.6(pol) |
| Chr18 | gene | 15849846 | 15851147 | + | **Vitis18g01545** | |  |  |
| Chr18 | gene | 15874563 | 15874904 | + | Vitis18g01546 |  |  |  |
| Chr18 | gene | 15875360 | 15885109 | + | Vitis18g01547 |  |  |  |
| Chr18 | gene | 15922334 | 15922747 | + | Vitis18g01548 |  |  |  |
| Chr18 | gene | 15924922 | 15925305 | - | Vitis18g01549 | P92523 | 44.3 | Uncharacterized mitochondrial protein AtMg00860(AtMg00860) |
| Chr18 | gene | 15924922 | 15925662 | - | Vitis18g01550 | P04323 | 36.8 | Retrovirus-related Pol polyprotein from transposon 17.6(pol) |
| Chr18 | gene | 15942246 | 15942713 | + | **Vitis18g01551** | |  |  |
| Chr18 | gene | 15967391 | 15968170 | + | **Vitis18g01552** | P92523 | 41 | Uncharacterized mitochondrial protein AtMg00860(AtMg00860) |
| Chr18 | gene | 15982017 | 15984698 | - | **Vitis18g01553** | |  |  |
| Chr18 | gene | 15987600 | 15990182 | - | Vitis18g01554 | Q8VZZ4 | 51.9 | multidrug resistance-associated protein 8(ABCC6) |
| Chr18 | gene | 15992886 | 15993125 | + | Vitis18g01555 |  |  |  |
| Chr18 | gene | 16053173 | 16053529 | - | Vitis18g01556 |  |  |  |
| Chr18 | gene | 16061247 | 16065246 | - | Vitis18g01557 |  |  |  |
| Chr18 | gene | 16065370 | 16065912 | - | Vitis18g01558 | P04323 | 32 | Retrovirus-related Pol polyprotein from transposon 17.6(pol) |
| Chr18 | gene | 16107616 | 16108140 | + | **Vitis18g01559** | P03371 | 39.3 | Pol polyprotein(pol) |
| Chr18 | gene | 16109207 | 16110048 | + | **Vitis18g01560** | |  |  |
| Chr18 | gene | 16110255 | 16110632 | - | **Vitis18g01561** | |  |  |
| Chr18 | gene | 16206342 | 16209035 | + | **Vitis18g01562** | P04323 | 38.1 | Retrovirus-related Pol polyprotein from transposon 17.6(pol) |
| Chr18 | gene | 16212640 | 16213020 | - | **Vitis18g01563** | P20825 | 42.9 | Retrovirus-related Pol polyprotein from transposon 297(pol) |
| Chr18 | gene | 16232351 | 16232944 | + | **Vitis18g01564** | |  |  |
| Chr18 | gene | 16338589 | 16359127 | - | **Vitis18g01565** | |  |  |
| Chr18 | gene | 16374807 | 16375182 | - | **Vitis18g01566** | |  |  |
| Chr18 | gene | 16449228 | 16451283 | - | Vitis18g01567 | Q01525 | 41.1 | general regulatory factor 2(GRF2) |
| Chr18 | gene | 16474946 | 16475388 | - | Vitis18g01568 | F4I171 | 67.7 | mediator of RNA polymerase II transcription subunit 15a-like protein(NRB4) |
| Chr18 | gene | 16541223 | 16542665 | - | Vitis18g01569 | Q6ZI17 | 83 | protein MEI2-like 2(LOC4330544) |
| Chr18 | gene | 16543010 | 16543997 | + | Vitis18g01570 |  |  |  |
| Chr18 | gene | 16543616 | 16545832 | + | Vitis18g01571 | Q94HW2 | 49.1 | Retrovirus-related Pol polyprotein from transposon RE1(RE1) |
| Chr18 | gene | 16557312 | 16558316 | - | **Vitis18g01572** | P04323 | 42.1 | Retrovirus-related Pol polyprotein from transposon 17.6(pol) |
| Chr18 | gene | 16570243 | 16572558 | + | **Vitis18g01573** | P10978 | 30.7 | Retrovirus-related Pol polyprotein from transposon TNT 1-94 (POLX_TOBAC) |
| Chr18 | gene | 16572667 | 16574223 | + | **Vitis18g01574** | P10978 | 43.3 | Retrovirus-related Pol polyprotein from transposon TNT 1-94 (POLX_TOBAC) |
| Chr18 | gene | 16572724 | 16574223 | + | **Vitis18g01575** | P10978 | 43.8 | Retrovirus-related Pol polyprotein from transposon TNT 1-94 (POLX_TOBAC) |
| Chr18 | gene | 16592661 | 16601118 | + | Vitis18g01576 | F4I171 | 69.4 | mediator of RNA polymerase II transcription subunit 15a-like protein(NRB4) |
| Chr18 | gene | 16624295 | 16648464 | - | Vitis18g01577 |  |  |  |
| Chr18 | gene | 16648509 | 16651591 | - | **Vitis18g01578** | |  |  |
| Chr18 | gene | 16692581 | 16719672 | - | **Vitis18g01579** | |  |  |
| Chr18 | gene | 16754030 | 16757251 | - | **Vitis18g01580** | |  |  |
| Chr18 | gene | 16811049 | 16812944 | - | **Vitis18g01581** | P04323 | 37.7 | Retrovirus-related Pol polyprotein from transposon 17.6(pol) |
| Chr18 | gene | 16813093 | 16814052 | - | **Vitis18g01582** | |  |  |
| Chr18 | gene | 16847161 | 16847817 | + | **Vitis18g01583** | Q8I7P9 | 36.7 | Retrovirus-related Pol polyprotein from transposon opus(pol) |
| Chr18 | gene | 16851607 | 16852344 | - | **Vitis18g01584** | |  |  |
| Chr18 | gene | 16854410 | 16857566 | - | **Vitis18g01585** | |  |  |
| Chr18 | gene | 16857618 | 16857944 | + | **Vitis18g01586** | |  |  |
| Chr18 | gene | 16860270 | 16866068 | + | **Vitis18g01587** | |  |  |
| Chr18 | gene | 16897981 | 16898760 | - | **Vitis18g01588** | |  |  |
| Chr18 | gene | 16927737 | 16928294 | + | Vitis18g01589 | P0CT42 | 31.3 | retrotransposable element(Tf2-7) |
| Chr18 | gene | 16991145 | 16993654 | + | **Vitis18g01590** | P04323 | 38.4 | Retrovirus-related Pol polyprotein from transposon 17.6(pol) |
| Chr18 | gene | 16993208 | 16993654 | + | **Vitis18g01591** | Q8I7P9 | 34.4 | Retrovirus-related Pol polyprotein from transposon opus(pol) |
| Chr18 | gene | 16994313 | 16994987 | + | **Vitis18g01592** | P03371 | 29.5 | Pol polyprotein(pol) |
| Chr18 | gene | 16995034 | 16995612 | + | **Vitis18g01593** | |  |  |
| Chr18 | gene | 17029499 | 17030185 | + | **Vitis18g01594** | |  |  |
| Chr18 | gene | 17030263 | 17031468 | + | **Vitis18g01595** | P04323 | 34.9 | Retrovirus-related Pol polyprotein from transposon 17.6(pol) |
| Chr18 | gene | 17032972 | 17033637 | + | **Vitis18g01596** | P32542 | 33.3 | Pol polyprotein(pol) |
| Chr18 | gene | 17086285 | 17086842 | + | **Vitis18g01597** | P04323 | 40.8 | Retrovirus-related Pol polyprotein from transposon 17.6(pol) |
| Chr18 | gene | 17214547 | 17215593 | + | **Vitis18g01598** | |  |  |
| Chr18 | gene | 17215769 | 17216221 | + | **Vitis18g01599** | |  |  |
| Chr18 | gene | 17216440 | 17217213 | + | **Vitis18g01600** | |  |  |
| Chr18 | gene | 17217315 | 17219676 | + | **Vitis18g01601** | P04323 | 34.8 | Retrovirus-related Pol polyprotein from transposon 17.6(pol) |
| Chr18 | gene | 17217937 | 17219676 | + | **Vitis18g01602** | P04323 | 47 | Retrovirus-related Pol polyprotein from transposon 17.6(pol) |
| Chr18 | gene | 17237944 | 17238336 | + | **Vitis18g01603** | |  |  |
| Chr18 | gene | 17248684 | 17259566 | + | **Vitis18g01604** | |  |  |
| Chr18 | gene | 17284393 | 17285187 | + | **Vitis18g01605** | P92516 | 64.9 | Uncharacterized mitochondrial protein AtMg00750(AtMg00750) |
| Chr18 | gene | 17286524 | 17286823 | - | **Vitis18g01606** | |  |  |
| Chr18 | gene | 17308690 | 17309100 | - | Vitis18g01607 | P92523 | 39.6 | Uncharacterized mitochondrial protein AtMg00860(AtMg00860) |
| Chr18 | gene | 17308989 | 17309588 | - | Vitis18g01608 |  |  |  |
| Chr18 | gene | 17310639 | 17310986 | - | **Vitis18g01609** | |  |  |
| Chr18 | gene | 17324767 | 17325432 | + | **Vitis18g01610** | |  |  |
| Chr18 | gene | 17346880 | 17347293 | + | **Vitis18g01611** | Q8I7P9 | 42.5 | Retrovirus-related Pol polyprotein from transposon opus(pol) |
| Chr18 | gene | 17347423 | 17348295 | + | **Vitis18g01612** | |  |  |
| Chr18 | gene | 17360911 | 17364665 | - | **Vitis18g01613** | |  |  |
| Chr18 | gene | 17404662 | 17405372 | + | **Vitis18g01614** | P04323 | 35.8 | Retrovirus-related Pol polyprotein from transposon 17.6(pol) |
| Chr18 | gene | 17413158 | 17413484 | + | **Vitis18g01615** | |  |  |
| Chr18 | gene | 17414110 | 17414550 | + | **Vitis18g01616** | |  |  |
| Chr18 | gene | 17414978 | 17415841 | + | **Vitis18g01617** | |  |  |
| Chr18 | gene | 17443510 | 17444022 | - | **Vitis18g01618** | |  |  |
| Chr18 | gene | 17470764 | 17483599 | + | Vitis18g01619 |  |  |  |
| Chr18 | gene | 17593608 | 17594093 | - | **Vitis18g01620** | P03371 | 31.6 | Pol polyprotein(pol) |
| Chr18 | gene | 17594409 | 17594951 | - | **Vitis18g01621** | Q8I7P9 | 44.8 | Retrovirus-related Pol polyprotein from transposon opus(pol) |
| Chr18 | gene | 17595009 | 17595482 | - | **Vitis18g01622** | Q9TTC1 | 24.2 | Gag-Pol polyprotein(pro-pol) |
| Chr18 | gene | 17617259 | 17617528 | + | **Vitis18g01623** | |  |  |
| Chr18 | gene | 17618908 | 17619507 | - | **Vitis18g01624** | |  |  |
| Chr18 | gene | 17619604 | 17620679 | - | **Vitis18g01625** | P04323 | 33.8 | Retrovirus-related Pol polyprotein from transposon 17.6(pol) |
| Chr18 | gene | 17626849 | 17627346 | - | Vitis18g01626 |  |  |  |
| Chr18 | gene | 17627398 | 17627637 | - | **Vitis18g01627** | |  |  |
| Chr18 | gene | 17628824 | 17630221 | - | **Vitis18g01628** | |  |  |
| Chr18 | gene | 17711953 | 17712171 | - | **Vitis18g01629** | |  |  |
| Chr19 | - | - | - | - | **-** | |  |  |

**Table S8 Gene Cluster in PN_T2T.**

| **Pfam_ID** | **Gene_ID** | **Count** |
| --- | --- | --- |
| PF00004 | Vitis14g02252,Vitis14g02253,Vitis14g02254,Vitis14g02255,Vitis14g02256,Vitis14g02259,Vitis14g02261,Vitis14g02262 | 8 |
| PF00005 | Vitis02g00089,Vitis02g00090,Vitis02g00091,Vitis02g00092,Vitis02g00093,Vitis02g00094,Vitis02g00095,Vitis09g00557,Vitis09g00559,Vitis09g00560,Vitis09g00565,Vitis09g00566,Vitis09g00568,Vitis09g00572,Vitis09g00573,Vitis09g00574,Vitis09g00575,Vitis09g00576,Vitis09g00578,Vitis09g00582 | 20 |
| PF00010 | Vitis01g00858,Vitis01g00875,Vitis01g00878,Vitis01g00883,Vitis01g00884,Vitis01g00885,Vitis01g00886,Vitis01g00887,Vitis01g00893,Vitis01g00895,Vitis01g00896,Vitis07g01922,Vitis07g01938,Vitis07g01939,Vitis07g01940,Vitis07g01941,Vitis07g01942 | 17 |
| PF00011 | Vitis04g00146,Vitis04g00147,Vitis04g00148,Vitis04g00149,Vitis04g00150,Vitis04g00151,Vitis04g00152,Vitis04g00154,Vitis04g00155,Vitis04g00156,Vitis04g00157,Vitis04g00158,Vitis04g00159 | 13 |
| PF00043 | Vitis17g00294,Vitis17g00295,Vitis17g00296,Vitis17g00298 | 4 |
| PF00060 | Vitis04g00788,Vitis04g00791,Vitis04g00793,Vitis04g00795,Vitis04g00797,Vitis04g00802,Vitis04g00803,Vitis04g00806,Vitis04g00814,Vitis04g00815,Vitis04g00816,Vitis04g00817,Vitis04g00821,Vitis04g00826,Vitis04g00827 | 15 |
| PF00067 | Vitis02g00446,Vitis02g00447,Vitis02g00448,Vitis02g00449,Vitis02g00501,Vitis02g00503,Vitis02g00504,Vitis02g00505,Vitis02g00551,Vitis02g00553,Vitis02g00558,Vitis02g00559,Vitis02g00560,Vitis02g00561,Vitis02g00564,Vitis06g01365,Vitis06g01366,Vitis06g01367,Vitis06g01368,Vitis06g01369,Vitis06g01371,Vitis06g01372,Vitis06g01374,Vitis06g01375,Vitis06g01376,Vitis06g01377,Vitis06g01379,Vitis06g01380,Vitis06g01381,Vitis06g01382,Vitis06g01383,Vitis06g01384,Vitis06g01386,Vitis06g01387,Vitis06g01388,Vitis06g01389,Vitis06g01391,Vitis06g01394,Vitis06g01395,Vitis06g01396,Vitis06g01398,Vitis06g01399,Vitis07g02077,Vitis07g02078,Vitis07g02079,Vitis07g02082,Vitis07g02083,Vitis07g02086,Vitis07g02087,Vitis07g02092,Vitis07g02098,Vitis07g02263,Vitis07g02281,Vitis07g02290,Vitis07g02291,Vitis07g02292,Vitis07g02293,Vitis13g00191,Vitis13g00192,Vitis13g00196,Vitis13g00197,Vitis13g00198,Vitis13g00199,Vitis13g00200,Vitis13g00201,Vitis13g00202,Vitis15g00651,Vitis15g00653,Vitis15g00654,Vitis15g00655,Vitis15g01009,Vitis15g01014,Vitis15g01015,Vitis15g01017,Vitis15g01019,Vitis15g01021,Vitis15g01023,Vitis15g01024,Vitis15g01026,Vitis15g01031,Vitis15g01032,Vitis15g01041,Vitis15g01042,Vitis15g01043,Vitis16g00974,Vitis16g00983,Vitis16g00985,Vitis16g00988,Vitis18g00111,Vitis18g00112,Vitis18g00113,Vitis18g00115,Vitis18g00961,Vitis18g00970,Vitis18g00971,Vitis18g00972,Vitis18g00975,Vitis18g00977,Vitis18g00978,Vitis18g00979,Vitis18g00983,Vitis18g00984,Vitis18g00985,Vitis18g00986,Vitis18g00987,Vitis18g00988,Vitis18g00989,Vitis18g00990,Vitis18g00994,Vitis18g01198,Vitis18g01199,Vitis18g01200,Vitis18g01201,Vitis18g01202,Vitis18g01203,Vitis18g01206,Vitis18g01207,Vitis18g01211,Vitis18g01212,Vitis19g01049,Vitis19g01053,Vitis19g01054,Vitis19g01055,Vitis19g01060,Vitis19g01061,Vitis19g01063,Vitis19g01065,Vitis19g01085,Vitis19g01091 | 129 |
| PF00069 | Vitis04g00009,Vitis04g00026,Vitis04g00027,Vitis04g00028,Vitis04g00029,Vitis04g00030,Vitis04g00031,Vitis04g00032,Vitis04g00033,Vitis04g00034,Vitis04g00035,Vitis04g00036,Vitis04g00038,Vitis04g00039,Vitis04g00041,Vitis04g00042,Vitis04g00051,Vitis06g00167,Vitis06g00168,Vitis06g00169,Vitis06g00170,Vitis06g00171,Vitis06g00172,Vitis06g00177,Vitis06g00178,Vitis08g01177,Vitis08g01192,Vitis08g01193,Vitis08g01195,Vitis08g01196,Vitis08g01197,Vitis08g01199,Vitis08g01200,Vitis09g00280,Vitis09g00286,Vitis09g00287,Vitis09g00288,Vitis09g00292,Vitis09g00304,Vitis09g00308,Vitis12g00358,Vitis12g00374,Vitis12g00375,Vitis12g00388,Vitis12g00389,Vitis16g01586,Vitis16g01589,Vitis16g01591,Vitis16g01592,Vitis16g01595,Vitis16g01597,Vitis16g01598,Vitis16g01603,Vitis16g01607,Vitis16g01612,Vitis16g01613,Vitis16g01615,Vitis16g01616,Vitis16g01618,Vitis16g01620,Vitis16g01624,Vitis16g01625,Vitis16g01627,Vitis16g01628,Vitis16g01633,Vitis16g01637,Vitis16g01639,Vitis16g01640,Vitis16g01644,Vitis16g01646,Vitis16g01650,Vitis16g01651,Vitis16g01656,Vitis16g01663,Vitis16g01668,Vitis16g01670,Vitis16g01672,Vitis16g01674,Vitis16g01677,Vitis16g01679,Vitis16g01681,Vitis16g01686,Vitis16g01689,Vitis16g01692,Vitis16g01694,Vitis16g01697,Vitis16g01701,Vitis16g01723,Vitis16g01725,Vitis16g01727,Vitis18g00783,Vitis18g00801,Vitis18g00802,Vitis18g00803,Vitis18g00804,Vitis18g00805,Vitis18g00807,Vitis18g00808,Vitis18g00810,Vitis18g00829 | 100 |
| PF00078 | Vitis04g00877,Vitis04g00879,Vitis04g00881,Vitis04g00882,Vitis04g00883,Vitis04g00884,Vitis04g00888,Vitis04g00893,Vitis05g00992,Vitis05g00993,Vitis05g00995,Vitis05g01004,Vitis05g01018,Vitis05g01019,Vitis05g01029,Vitis05g01033,Vitis05g01041,Vitis05g01042,Vitis05g01043,Vitis05g01045,Vitis05g01048,Vitis05g01302,Vitis05g01312,Vitis05g01318,Vitis05g01319,Vitis05g01321,Vitis05g01336,Vitis05g01338,Vitis05g01340,Vitis05g01343,Vitis05g01344,Vitis05g01347,Vitis05g01348,Vitis05g01444,Vitis05g01446,Vitis05g01449,Vitis05g01452,Vitis05g01455,Vitis05g01456,Vitis05g01457,Vitis05g01467,Vitis05g01468,Vitis05g01469,Vitis05g01472,Vitis05g01473,Vitis05g01474,Vitis05g01476,Vitis05g01478,Vitis05g01480,Vitis05g01482,Vitis05g01695,Vitis05g01704,Vitis05g01705,Vitis05g01707,Vitis05g01714,Vitis05g01715,Vitis05g01718,Vitis05g01721,Vitis05g01725,Vitis05g01733,Vitis05g01742,Vitis05g01743,Vitis05g01744,Vitis07g01553,Vitis07g01556,Vitis07g01560,Vitis07g01561,Vitis07g01565,Vitis07g01578,Vitis07g01579,Vitis07g01581,Vitis07g01583,Vitis07g02510,Vitis07g02513,Vitis07g02522,Vitis07g02523,Vitis07g02524,Vitis08g00002,Vitis08g00006,Vitis08g00020,Vitis09g00259,Vitis11g01081,Vitis11g01095,Vitis11g01102,Vitis11g01104,Vitis11g01106,Vitis11g01107,Vitis11g01109,Vitis11g01115,Vitis11g01117,Vitis11g01118,Vitis11g01123,Vitis11g01131,Vitis11g01133,Vitis12g00080,Vitis12g00094,Vitis12g00105,Vitis12g00106,Vitis12g00108,Vitis12g00110,Vitis12g00126,Vitis12g00133,Vitis13g02259,Vitis13g02273,Vitis13g02276,Vitis13g02285,Vitis13g02286,Vitis13g02289,Vitis18g01534,Vitis18g01542,Vitis18g01544,Vitis18g01550,Vitis18g01558,Vitis18g01562,Vitis18g01563,Vitis18g01581,Vitis18g01583,Vitis18g01590,Vitis18g02445,Vitis18g02458,Vitis18g02476,Vitis19g01604,Vitis19g01605,Vitis19g01608,Vitis19g01770,Vitis19g01773,Vitis19g01781,Vitis19g01782,Vitis19g01784 | 129 |
| PF00082 | Vitis02g00494,Vitis02g00495,Vitis02g00496,Vitis02g00497,Vitis02g00498,Vitis02g00513,Vitis13g00463,Vitis13g00464,Vitis13g00465,Vitis13g00466,Vitis13g00469,Vitis13g00470,Vitis13g00471,Vitis13g00472,Vitis13g00473,Vitis13g00474,Vitis13g00476 | 17 |
| PF00083 | Vitis14g00456,Vitis14g00457,Vitis14g00459,Vitis14g00460,Vitis14g00461,Vitis14g00462,Vitis14g00464,Vitis14g00465,Vitis14g00466,Vitis14g00467,Vitis14g00468 | 11 |
| PF00107 | Vitis05g02081,Vitis05g02082,Vitis05g02083,Vitis05g02084,Vitis05g02085,Vitis05g02086,Vitis05g02088,Vitis05g02089 | 8 |
| PF00112 | Vitis12g00255,Vitis12g00256,Vitis12g00257,Vitis12g00259 | 4 |
| PF00122 | Vitis12g00883,Vitis12g00885,Vitis12g00886,Vitis12g00887,Vitis12g00888 | 5 |
| PF00141 | Vitis01g01570,Vitis01g01572,Vitis01g01573,Vitis01g01576,Vitis01g01577,Vitis01g01578,Vitis01g01579,Vitis01g01580,Vitis01g01581,Vitis01g01582,Vitis01g01583,Vitis01g01584,Vitis14g01872,Vitis14g01873,Vitis14g01874,Vitis14g01875 | 16 |
| PF00168 | Vitis16g01424,Vitis16g01425,Vitis16g01426,Vitis16g01427,Vitis16g01433,Vitis16g01434,Vitis16g01435,Vitis16g01436 | 8 |
| PF00182 | Vitis05g01995,Vitis05g01997,Vitis05g01999,Vitis05g02000,Vitis05g02001,Vitis05g02002,Vitis05g02005,Vitis05g02006,Vitis05g02007,Vitis05g02008,Vitis05g02009,Vitis05g02010,Vitis05g02011,Vitis05g02012 | 14 |
| PF00188 | Vitis03g00978,Vitis03g00980,Vitis03g00981,Vitis03g00982,Vitis03g00983,Vitis03g00984,Vitis03g00986,Vitis03g00987,Vitis03g00993,Vitis03g00998,Vitis03g01000,Vitis03g01002,Vitis03g01003,Vitis03g01016 | 14 |
| PF00190 | Vitis07g00958,Vitis07g00964,Vitis07g00967,Vitis07g00968,Vitis07g00972,Vitis07g00973,Vitis07g00980,Vitis12g01573,Vitis12g01574,Vitis12g01575,Vitis12g01576,Vitis12g01577,Vitis12g01578,Vitis12g01581,Vitis12g01582,Vitis12g01587,Vitis12g01588,Vitis14g00314,Vitis14g00315,Vitis14g00316,Vitis14g00317,Vitis14g00318,Vitis14g00319,Vitis14g00320,Vitis14g00321,Vitis14g00322,Vitis14g00324,Vitis14g00325,Vitis14g00326,Vitis14g00329,Vitis14g00330,Vitis14g00331,Vitis14g00332,Vitis14g00334,Vitis14g00358,Vitis14g00359,Vitis14g00360,Vitis14g00362,Vitis14g00363,Vitis14g00364,Vitis14g00365,Vitis14g00366,Vitis14g00367,Vitis14g00368,Vitis14g00369,Vitis14g00370,Vitis14g00371 | 47 |
| PF00191 | Vitis13g01832,Vitis13g01838,Vitis13g01841,Vitis13g01846,Vitis13g01847,Vitis13g01849,Vitis13g01851,Vitis13g01852,Vitis13g01859,Vitis13g01860,Vitis13g01861,Vitis13g01862,Vitis13g01865,Vitis13g01868,Vitis13g01869,Vitis13g01870,Vitis13g01873,Vitis13g01874,Vitis13g01876,Vitis13g01880,Vitis13g01881 | 21 |
| PF00195 | Vitis16g01203,Vitis16g01204,Vitis16g01205,Vitis16g01206,Vitis16g01208,Vitis16g01209,Vitis16g01210,Vitis16g01211,Vitis16g01212,Vitis16g01213,Vitis16g01214,Vitis16g01215,Vitis16g01217,Vitis16g01219,Vitis16g01220,Vitis16g01222,Vitis16g01223,Vitis16g01224,Vitis16g01225,Vitis16g01226,Vitis16g01227,Vitis16g01228,Vitis16g01229,Vitis16g01232,Vitis16g01233,Vitis16g01237,Vitis16g01238,Vitis16g01239,Vitis16g01242,Vitis16g01245,Vitis16g01246 | 31 |
| PF00197 | Vitis17g01363,Vitis17g01367,Vitis17g01373,Vitis17g01374,Vitis17g01380,Vitis17g01381,Vitis17g01384 | 7 |
| PF00201 | Vitis07g01765,Vitis07g01766,Vitis07g01767,Vitis07g01768,Vitis07g01770,Vitis08g01555,Vitis08g01556,Vitis08g01557,Vitis08g01558,Vitis08g01559,Vitis08g01560,Vitis12g01356,Vitis12g01357,Vitis12g01360,Vitis12g01361,Vitis12g01362,Vitis12g01365,Vitis12g01373,Vitis12g01375,Vitis12g01376,Vitis12g01377,Vitis12g01378,Vitis12g01380,Vitis12g01381,Vitis12g01382,Vitis12g01384,Vitis12g01388,Vitis12g01392,Vitis12g01395,Vitis12g01400,Vitis12g01402,Vitis12g01406,Vitis12g01408,Vitis13g00486,Vitis13g00487,Vitis13g00489,Vitis13g00493,Vitis13g00494,Vitis13g00497,Vitis13g00498,Vitis13g00499,Vitis13g00501,Vitis13g00507,Vitis13g00508,Vitis13g00509,Vitis13g00511,Vitis13g00513,Vitis14g01724,Vitis14g01725,Vitis14g01726,Vitis14g01728,Vitis14g01729,Vitis18g02479,Vitis18g02481,Vitis18g02483,Vitis18g02484,Vitis18g02485,Vitis18g02488,Vitis18g02489,Vitis18g02490,Vitis18g02493,Vitis18g02495,Vitis18g02496,Vitis18g02497,Vitis18g02498,Vitis18g02499,Vitis18g02504,Vitis18g02508,Vitis18g02510,Vitis18g02511 | 70 |
| PF00208 | Vitis16g00277,Vitis16g00278,Vitis16g00279,Vitis16g00280 | 4 |
| PF00232 | Vitis13g02185,Vitis13g02186,Vitis13g02189,Vitis13g02190,Vitis13g02191,Vitis13g02192,Vitis13g02193,Vitis13g02202,Vitis13g02203,Vitis13g02204,Vitis13g02205 | 11 |
| PF00248 | Vitis01g00653,Vitis01g00654,Vitis01g00656,Vitis01g00657,Vitis01g00658,Vitis01g00659,Vitis01g00660,Vitis01g00661,Vitis01g00662,Vitis01g00663 | 10 |
| PF00249 | Vitis02g01248,Vitis02g01256,Vitis02g01257,Vitis02g01259,Vitis02g01260,Vitis02g01265,Vitis02g01266,Vitis16g00114,Vitis16g00123,Vitis16g00126,Vitis16g00128,Vitis16g00131,Vitis16g00133,Vitis16g00134,Vitis16g00137,Vitis16g00139,Vitis16g00140,Vitis16g00143,Vitis16g00145,Vitis16g00148,Vitis16g00149,Vitis16g00150,Vitis16g00163 | 23 |
| PF00295 | Vitis12g00851,Vitis12g00853,Vitis12g00854,Vitis12g00855,Vitis12g00856,Vitis12g00857,Vitis16g01399,Vitis16g01400,Vitis16g01401,Vitis16g01402,Vitis19g01592,Vitis19g01593,Vitis19g01595,Vitis19g01598 | 14 |
| PF00305 | Vitis06g00187,Vitis06g00189,Vitis06g00190,Vitis06g00191,Vitis06g00193,Vitis06g00194,Vitis06g00196,Vitis06g00205 | 8 |
| PF00314 | Vitis02g00433,Vitis02g00434,Vitis02g00435,Vitis02g00436,Vitis02g00437,Vitis02g00439,Vitis02g00440,Vitis02g00441,Vitis02g00442,Vitis02g00443,Vitis02g00444 | 11 |
| PF00319 | Vitis05g00356,Vitis05g00357,Vitis05g00358,Vitis05g00359,Vitis05g00360,Vitis10g01092,Vitis10g01093,Vitis10g01094,Vitis10g01095 | 9 |
| PF00331 | Vitis03g00843,Vitis03g00845,Vitis03g00846,Vitis03g00848 | 4 |
| PF00332 | Vitis08g01727,Vitis08g01728,Vitis08g01729,Vitis08g01730,Vitis08g01731,Vitis08g01732,Vitis08g01733,Vitis08g01735,Vitis08g01736,Vitis08g01737 | 10 |
| PF00407 | Vitis01g00498,Vitis01g00499,Vitis01g00502,Vitis01g00503,Vitis01g00504,Vitis01g00505,Vitis01g00506,Vitis01g00509,Vitis01g00515,Vitis01g00516,Vitis05g00074,Vitis05g00075,Vitis05g00076,Vitis05g00077,Vitis05g00078,Vitis05g00079,Vitis05g00083,Vitis05g00084,Vitis05g00085,Vitis05g00086,Vitis05g00087,Vitis05g00088,Vitis05g00089,Vitis05g00090,Vitis05g00091 | 25 |
| PF00497 | Vitis04g00793,Vitis04g00795,Vitis04g00797,Vitis04g00802,Vitis04g00803,Vitis04g00806,Vitis04g00814,Vitis04g00815,Vitis04g00816,Vitis04g00817,Vitis04g00820,Vitis04g00821,Vitis04g00826,Vitis04g00827 | 14 |
| PF00560 | Vitis09g01458,Vitis09g01459,Vitis09g01460,Vitis09g01463,Vitis09g01467,Vitis09g01473,Vitis09g01474,Vitis09g01479,Vitis09g01484,Vitis09g01485,Vitis09g01487,Vitis09g01489,Vitis09g01491,Vitis09g01492,Vitis09g01494,Vitis09g01495,Vitis09g01498,Vitis09g01499,Vitis09g01500,Vitis09g01503,Vitis09g01519,Vitis14g01904,Vitis14g01908,Vitis14g01910,Vitis14g01915,Vitis14g01919,Vitis14g01921,Vitis14g01924,Vitis14g01925,Vitis14g01928,Vitis14g01930,Vitis14g01931,Vitis14g01936,Vitis14g01940,Vitis14g01946,Vitis14g01948,Vitis14g01950 | 37 |
| PF00561 | Vitis12g00455,Vitis12g00456,Vitis12g00457,Vitis12g00458 | 4 |
| PF00574 | Vitis02g00724,Vitis02g00725 | 2 |
| PF00646 | Vitis13g00325,Vitis13g00326,Vitis13g00327,Vitis13g00328 | 4 |
| PF00657 | Vitis14g01621,Vitis14g01623,Vitis14g01624,Vitis14g01625 | 4 |
| PF00658 | Vitis18g00839,Vitis18g00841,Vitis18g00843,Vitis18g00845 | 4 |
| PF00665 | Vitis02g01124,Vitis02g01147,Vitis02g01148,Vitis02g01153,Vitis02g01156,Vitis02g01163,Vitis02g01164,Vitis02g01166,Vitis05g01154,Vitis05g01157,Vitis05g01158,Vitis05g01161,Vitis05g01163,Vitis05g01165,Vitis05g01167,Vitis05g01172,Vitis05g01173,Vitis05g01176,Vitis05g01177,Vitis05g01180,Vitis05g01181,Vitis05g01188,Vitis05g01189,Vitis05g01208,Vitis06g00984,Vitis06g00985,Vitis06g00990,Vitis06g00991,Vitis06g00996,Vitis06g00999,Vitis06g01001,Vitis06g01006,Vitis06g01012,Vitis06g01014,Vitis06g01016,Vitis06g01020,Vitis06g01044,Vitis06g01045,Vitis08g00403,Vitis08g00421,Vitis08g00423,Vitis08g00429,Vitis08g00440,Vitis08g00441,Vitis08g00443,Vitis08g00444,Vitis08g00452,Vitis08g00455,Vitis10g01154,Vitis10g01163,Vitis10g01179,Vitis10g01181,Vitis11g01150,Vitis11g01153,Vitis11g01154,Vitis11g01155,Vitis11g01157,Vitis11g01161,Vitis11g01164,Vitis11g01166,Vitis11g01172,Vitis11g01173,Vitis11g01176,Vitis11g01177,Vitis11g01179,Vitis11g01181,Vitis11g01193,Vitis14g01070,Vitis16g00596,Vitis16g00598,Vitis16g00599,Vitis16g00619,Vitis16g00621,Vitis16g00626,Vitis16g00636,Vitis18g01532,Vitis18g01533,Vitis18g01534,Vitis18g01535,Vitis18g01536,Vitis18g01539,Vitis18g01540,Vitis18g01542,Vitis18g01545,Vitis18g01559,Vitis18g01562,Vitis18g01564,Vitis18g01573,Vitis18g01592,Vitis18g02502 | 90 |
| PF00685 | Vitis13g01589,Vitis13g01604,Vitis13g01605,Vitis13g01607,Vitis13g02056,Vitis13g02057,Vitis13g02058,Vitis13g02059,Vitis13g02063,Vitis13g02064 | 10 |
| PF00704 | Vitis11g00774,Vitis11g00775,Vitis11g00776,Vitis11g00777,Vitis11g00778,Vitis11g00779,Vitis11g00782,Vitis11g00783,Vitis11g00784,Vitis15g01329,Vitis15g01330,Vitis15g01331,Vitis15g01332,Vitis15g01333,Vitis15g01334,Vitis15g01342,Vitis15g01343,Vitis15g01344 | 18 |
| PF00722 | Vitis11g01487,Vitis11g01488,Vitis11g01489,Vitis11g01494,Vitis11g01495,Vitis11g01496,Vitis11g01499,Vitis11g01500,Vitis11g01501 | 9 |
| PF00743 | Vitis03g00285,Vitis03g00286,Vitis03g00287,Vitis03g00288,Vitis03g00289,Vitis03g00290 | 6 |
| PF00847 | Vitis07g00688,Vitis07g00689,Vitis07g00690,Vitis07g00691,Vitis07g00692,Vitis07g00693,Vitis07g00695,Vitis07g00696,Vitis07g00697,Vitis07g00698,Vitis16g00409,Vitis16g00410,Vitis16g00412,Vitis16g00413,Vitis16g00414,Vitis16g00415,Vitis16g00416,Vitis16g00418,Vitis16g00421,Vitis16g00422,Vitis16g00424,Vitis16g00425,Vitis16g00431,Vitis16g00432,Vitis16g00433,Vitis16g00435,Vitis16g00436,Vitis16g00437,Vitis16g00438 | 29 |
| PF00854 | Vitis18g02458,Vitis18g02460,Vitis18g02461,Vitis18g02462,Vitis18g02463,Vitis18g02466,Vitis18g02467,Vitis18g02468,Vitis18g02469,Vitis18g02470,Vitis18g02471,Vitis18g02473 | 12 |
| PF00891 | Vitis12g00301,Vitis12g00305,Vitis12g00307,Vitis12g00308,Vitis12g00310,Vitis12g00311,Vitis12g00312,Vitis12g00313,Vitis12g00314,Vitis12g00315,Vitis12g00316,Vitis12g00317,Vitis12g00318,Vitis12g00319 | 14 |
| PF00892 | Vitis01g00937,Vitis01g00946,Vitis01g00947,Vitis01g00949,Vitis01g00950,Vitis01g00951,Vitis04g01808,Vitis04g01809,Vitis04g01811,Vitis04g01812,Vitis04g01813,Vitis04g01815,Vitis04g01816,Vitis04g01817,Vitis04g01818,Vitis04g01819,Vitis04g01821,Vitis04g01822,Vitis04g01823,Vitis04g01824 | 20 |
| PF00917 | Vitis03g00874,Vitis03g00875,Vitis03g00876,Vitis03g00877,Vitis03g00881 | 15 |
| PF00931 | Vitis01g00086,Vitis01g00087,Vitis01g00088,Vitis01g00093,Vitis01g00096,Vitis01g00097,Vitis01g00098,Vitis01g00101,Vitis01g00112,Vitis03g00121,Vitis03g00130,Vitis03g00131,Vitis03g00132,Vitis03g00133,Vitis03g00134,Vitis03g00135,Vitis03g00136,Vitis03g00137,Vitis03g00138,Vitis03g00139,Vitis03g00141,Vitis03g00146,Vitis03g00147,Vitis03g00148,Vitis03g00149,Vitis03g00153,Vitis03g00154,Vitis07g01655,Vitis07g01656,Vitis07g01659,Vitis09g00367,Vitis09g00370,Vitis09g00371,Vitis09g00372,Vitis09g00373,Vitis09g00375,Vitis09g00376,Vitis09g00482,Vitis09g00483,Vitis09g00484,Vitis09g00485,Vitis09g00486,Vitis09g00488,Vitis09g00489,Vitis09g00490,Vitis09g00496,Vitis09g00502,Vitis09g00504,Vitis09g00505,Vitis09g00509,Vitis09g00515,Vitis09g00537,Vitis09g00538,Vitis13g01736,Vitis13g01737,Vitis13g01738,Vitis13g01739,Vitis13g01740,Vitis13g01742,Vitis13g01749,Vitis13g01750,Vitis13g01751,Vitis13g01753,Vitis13g01755,Vitis13g01759,Vitis13g01760,Vitis13g01762,Vitis13g01763,Vitis13g01765,Vitis13g01771,Vitis13g01774,Vitis13g01776,Vitis13g01779,Vitis13g01995,Vitis13g02010,Vitis13g02012,Vitis13g02013,Vitis13g02015,Vitis13g02019,Vitis13g02020,Vitis13g02030,Vitis13g02032,Vitis13g02034,Vitis13g02043,Vitis14g00851,Vitis14g00853,Vitis14g00854,Vitis14g00856,Vitis14g00858,Vitis14g00859,Vitis14g00860,Vitis14g00865,Vitis14g00866,Vitis14g00869,Vitis14g00870,Vitis14g00874,Vitis14g00879,Vitis14g00883,Vitis14g00885,Vitis14g00889,Vitis15g00420,Vitis15g00421,Vitis15g00427,Vitis15g00436,Vitis15g00437,Vitis15g00439,Vitis15g00441,Vitis15g00445,Vitis15g00450,Vitis18g02281,Vitis18g02282,Vitis18g02286,Vitis18g02287,Vitis18g02290,Vitis18g02291,Vitis19g00557,Vitis19g00558,Vitis19g00561,Vitis19g00562,Vitis19g00563,Vitis19g00564,Vitis19g00574,Vitis19g01597,Vitis19g01602,Vitis19g01612,Vitis19g01615,Vitis19g01623,Vitis19g01624,Vitis19g01625,Vitis19g01626,Vitis19g01628,Vitis19g01629,Vitis19g01630,Vitis19g01632,Vitis19g01635,Vitis19g01637,Vitis19g01644,Vitis19g01648,Vitis19g01649,Vitis19g01652,Vitis19g01654,Vitis19g01655,Vitis19g01656 | 143 |
| PF00933 | Vitis05g00308,Vitis05g00309,Vitis05g00311,Vitis05g00312,Vitis05g00313 | 5 |
| PF01061 | Vitis09g00558,Vitis09g00559,Vitis09g00560,Vitis09g00563,Vitis09g00564,Vitis09g00565,Vitis09g00566,Vitis09g00567,Vitis09g00570,Vitis09g00571,Vitis09g00572,Vitis09g00574,Vitis09g00576,Vitis09g00582 | 14 |
| PF01095 | Vitis01g00534,Vitis11g00022,Vitis11g00023,Vitis11g00024,Vitis11g00025,Vitis11g00044,Vitis11g00046 | 7 |
| PF01190 | Vitis07g00409,Vitis07g00410,Vitis07g00411,Vitis07g00412 | 4 |
| PF01277 | Vitis14g02120,Vitis14g02121,Vitis14g02122,Vitis14g02123 | 4 |
| PF01357 | Vitis02g00723,Vitis02g00725,Vitis02g00726,Vitis02g00728 | 4 |
| PF01370 | Vitis13g01467,Vitis13g01468,Vitis13g01471,Vitis13g01475,Vitis13g01476,Vitis13g01482 | 6 |
| PF01397 | Vitis10g00468,Vitis10g00469,Vitis10g00475,Vitis10g00476,Vitis10g00481,Vitis10g00485,Vitis10g00492,Vitis10g00493,Vitis10g00495,Vitis10g00498,Vitis10g00499,Vitis10g00500,Vitis10g00501,Vitis10g00502,Vitis10g00504,Vitis13g01486,Vitis13g01487,Vitis13g01489,Vitis13g01495,Vitis13g01501,Vitis13g01502,Vitis13g01505,Vitis13g01506,Vitis13g01508,Vitis13g01510,Vitis13g01511,Vitis13g01513,Vitis13g01516,Vitis13g01517,Vitis13g01520,Vitis13g01521,Vitis13g01522,Vitis13g01525,Vitis13g01526,Vitis13g01533,Vitis13g01701,Vitis13g01706,Vitis13g01707,Vitis13g01708,Vitis13g01709,Vitis13g01710,Vitis13g01711,Vitis13g01714,Vitis13g01718,Vitis13g01720,Vitis18g02774,Vitis18g02776,Vitis18g02777,Vitis18g02779,Vitis18g02784,Vitis18g02785,Vitis18g02786,Vitis18g02789,Vitis18g02791,Vitis18g02799,Vitis18g02800,Vitis18g02805,Vitis18g02808,Vitis18g02812,Vitis18g02814,Vitis18g02815,Vitis18g02819,Vitis18g02821,Vitis18g02825,Vitis18g02826,Vitis18g02828,Vitis18g02837,Vitis18g02840,Vitis18g02841,Vitis18g02842,Vitis18g02843,Vitis18g02845,Vitis18g02846,Vitis18g02848,Vitis18g02851 | 75 |
| PF01425 | Vitis03g00172,Vitis03g00174,Vitis03g00175,Vitis03g00176,Vitis03g00177,Vitis03g00178,Vitis03g00179 | 7 |
| PF01453 | Vitis12g00374,Vitis12g00375,Vitis12g00377,Vitis12g00751,Vitis12g00752,Vitis12g00753,Vitis12g00757,Vitis12g00758,Vitis12g00759,Vitis12g00760,Vitis12g00764,Vitis12g00765,Vitis12g00766,Vitis12g00767,Vitis12g00772,Vitis12g00773,Vitis12g00774,Vitis12g00779,Vitis12g00783,Vitis12g00787,Vitis12g00788,Vitis12g00791,Vitis12g00797 | 23 |
| PF01490 | Vitis04g00019,Vitis04g00020,Vitis04g00021,Vitis04g00022,Vitis04g00024,Vitis19g01667,Vitis19g01668,Vitis19g01669,Vitis19g01670,Vitis19g01671 | 10 |
| PF01501 | Vitis14g00095,Vitis14g00096,Vitis14g00098,Vitis14g00099,Vitis14g00100,Vitis14g00102,Vitis14g00103 | 7 |
| PF01535 | Vitis04g00463,Vitis04g00468,Vitis04g00470,Vitis04g00471,Vitis04g00473 | 5 |
| PF01554 | Vitis08g00093,Vitis08g00100,Vitis08g00102,Vitis08g00103,Vitis08g00104,Vitis08g00110,Vitis08g00113,Vitis08g00117,Vitis08g00118,Vitis08g00121,Vitis08g00129,Vitis11g01525,Vitis11g01526,Vitis11g01527,Vitis11g01530,Vitis11g01531,Vitis11g01532,Vitis11g01533 | 18 |
| PF01596 | Vitis11g00269,Vitis11g00270,Vitis11g00272 | 3 |
| PF01657 | Vitis10g00408,Vitis10g00409,Vitis10g00414,Vitis10g00418,Vitis10g00420,Vitis10g00422,Vitis10g00423,Vitis10g00427,Vitis10g00429,Vitis10g00430,Vitis10g00432,Vitis10g00433,Vitis10g00436,Vitis10g00437,Vitis10g00438,Vitis10g00440,Vitis10g00441,Vitis10g00442,Vitis10g00446,Vitis10g00448,Vitis12g00694,Vitis12g00695,Vitis12g00696,Vitis12g00697,Vitis12g00698,Vitis12g00699,Vitis12g00700,Vitis12g00701 | 28 |
| PF01734 | Vitis18g00913,Vitis18g00917,Vitis18g00920,Vitis18g00921,Vitis18g00924,Vitis18g00925,Vitis18g00929,Vitis18g00930 | 8 |
| PF01762 | Vitis11g01474,Vitis11g01475,Vitis11g01476,Vitis11g01477 | 4 |
| PF01764 | Vitis10g01233,Vitis10g01234,Vitis10g01235,Vitis10g01236,Vitis10g01237,Vitis16g01728,Vitis16g01732,Vitis16g01733,Vitis16g01734,Vitis16g01735,Vitis16g01736,Vitis16g01738,Vitis16g01739 | 13 |
| PF02298 | Vitis13g01797,Vitis13g01798,Vitis13g01804,Vitis13g01805,Vitis13g01808,Vitis13g01812,Vitis13g01813,Vitis13g01823,Vitis13g01824,Vitis13g01828 | 10 |
| PF02362 | Vitis07g01630,Vitis07g01631,Vitis07g01632,Vitis07g01633,Vitis07g01634,Vitis07g01635 | 6 |
| PF02365 | Vitis15g01097,Vitis15g01098,Vitis15g01099,Vitis15g01100,Vitis15g01101,Vitis19g01509,Vitis19g01511,Vitis19g01512,Vitis19g01513,Vitis19g01516 | 10 |
| PF02458 | Vitis01g01626,Vitis01g01627,Vitis01g01629,Vitis01g01631,Vitis01g01632,Vitis01g01633,Vitis01g01634,Vitis01g01635,Vitis01g01636,Vitis02g01356,Vitis02g01358,Vitis02g01360,Vitis02g01362,Vitis02g01363,Vitis02g01370,Vitis02g01371,Vitis02g01372,Vitis02g01373,Vitis09g00977,Vitis09g00988,Vitis09g00990,Vitis09g00991,Vitis09g00992,Vitis09g01002,Vitis09g01003,Vitis09g01004,Vitis09g01005,Vitis09g01006,Vitis09g01028,Vitis09g01029,Vitis09g01030,Vitis12g00810,Vitis12g00811,Vitis12g00812,Vitis12g00813,Vitis12g00814,Vitis12g00815,Vitis12g00817,Vitis18g00839,Vitis18g00843,Vitis18g00844,Vitis18g00846,Vitis18g00847 | 43 |
| PF02469 | Vitis08g00947,Vitis08g00948,Vitis08g00949,Vitis08g00950,Vitis08g00951,Vitis08g00953,Vitis08g00954,Vitis08g00955,Vitis08g00956,Vitis08g00957,Vitis08g00958,Vitis08g00961 | 12 |
| PF02519 | Vitis03g00068,Vitis03g00069,Vitis03g00070,Vitis03g00088,Vitis03g00089,Vitis03g00090,Vitis03g00091,Vitis03g00092,Vitis03g00093,Vitis03g00094,Vitis03g00096,Vitis03g00097,Vitis03g00100,Vitis03g00101,Vitis03g00102,Vitis03g00103,Vitis03g00104,Vitis03g00105,Vitis03g00106,Vitis03g00107,Vitis03g00108,Vitis03g00109,Vitis03g00110,Vitis03g00111,Vitis03g00112,Vitis03g00113,Vitis03g00114,Vitis04g01297,Vitis04g01298,Vitis04g01299,Vitis04g01300,Vitis04g01302,Vitis04g01303,Vitis04g01304,Vitis04g01305,Vitis04g01306,Vitis04g01307,Vitis04g01308,Vitis04g01309,Vitis04g01310,Vitis04g01311,Vitis04g01312,Vitis04g01313,Vitis04g01314,Vitis04g01315,Vitis04g01316 | 46 |
| PF02535 | Vitis19g00817,Vitis19g00818,Vitis19g00819,Vitis19g00820 | 4 |
| PF02798 | Vitis05g00866,Vitis05g00867,Vitis05g00868,Vitis05g00870,Vitis05g00873,Vitis17g00717,Vitis17g00718,Vitis17g00719,Vitis17g00720,Vitis17g00722,Vitis17g00723 | 11 |
| PF02893 | Vitis10g01989,Vitis10g01990,Vitis10g01991,Vitis10g01992,Vitis10g01993,Vitis10g01996,Vitis10g01997,Vitis10g01998,Vitis10g01999,Vitis10g02001,Vitis10g02002,Vitis10g02003,Vitis10g02004,Vitis10g02007,Vitis10g02010,Vitis10g02011,Vitis10g02016,Vitis10g02017,Vitis10g02019,Vitis10g02020,Vitis10g02023 | 21 |
| PF03016 | Vitis11g00635,Vitis11g00636,Vitis11g00638,Vitis11g00648 | 4 |
| PF03018 | Vitis06g00112,Vitis06g00113,Vitis06g00114,Vitis06g00115,Vitis06g00116,Vitis06g00117,Vitis06g00118,Vitis08g01815,Vitis08g01816,Vitis08g01817,Vitis08g01819,Vitis08g01820,Vitis08g01821,Vitis08g01823,Vitis08g01824,Vitis12g00519,Vitis12g00520,Vitis12g00521,Vitis12g00522,Vitis12g00524,Vitis12g00525 | 21 |
| PF03087 | Vitis03g00306,Vitis03g00307,Vitis03g00308,Vitis03g00309,Vitis03g00310,Vitis03g00311,Vitis03g00312,Vitis03g00313,Vitis03g00314,Vitis03g00315,Vitis03g00316,Vitis03g00317,Vitis04g01719,Vitis04g01720,Vitis04g01721,Vitis04g01722,Vitis04g01723,Vitis04g01724,Vitis04g01725,Vitis04g01726,Vitis04g01729,Vitis04g01730,Vitis04g01731 | 23 |
| PF03088 | Vitis17g00516,Vitis17g00517,Vitis17g00518,Vitis17g00519,Vitis17g00520,Vitis17g00521 | 6 |
| PF03107 | Vitis01g00850,Vitis01g00851,Vitis01g00852,Vitis01g00853,Vitis01g00854 | 5 |
| PF03140 | Vitis04g00529,Vitis04g00531,Vitis04g00532,Vitis04g00533,Vitis04g00534,Vitis08g01676,Vitis08g01677,Vitis08g01679,Vitis08g01680,Vitis08g01682,Vitis08g01683,Vitis08g01684,Vitis08g01685,Vitis08g01686,Vitis08g01687,Vitis08g01688,Vitis08g01689,Vitis08g01690,Vitis08g01691,Vitis08g01692,Vitis08g01693,Vitis08g01695,Vitis14g00272,Vitis14g00275,Vitis14g00276,Vitis14g00277,Vitis14g00278,Vitis14g00279,Vitis14g00281,Vitis14g00287,Vitis14g00288,Vitis14g00292,Vitis14g00300,Vitis14g00302,Vitis14g00306,Vitis14g00307,Vitis14g00308 | 37 |
| PF03168 | Vitis02g00055,Vitis02g00056,Vitis02g00057,Vitis02g00058 | 4 |
| PF03171 | Vitis02g00813,Vitis02g00814,Vitis02g00815,Vitis02g00824,Vitis02g00825,Vitis02g00826,Vitis02g00828,Vitis05g00805,Vitis05g00806,Vitis05g00809,Vitis05g00810,Vitis05g00811,Vitis05g00812,Vitis05g00814,Vitis05g00815,Vitis05g00816,Vitis05g00817,Vitis05g00818,Vitis05g00820,Vitis05g00821,Vitis05g00822,Vitis09g00544,Vitis09g00546,Vitis09g00547,Vitis09g00551,Vitis09g00552,Vitis09g00554,Vitis09g00555,Vitis10g00998,Vitis10g00999,Vitis10g01000,Vitis10g01001,Vitis10g01002,Vitis10g01004,Vitis10g01007,Vitis10g01008,Vitis10g01010,Vitis10g01011,Vitis10g01013,Vitis10g01017,Vitis10g01018,Vitis10g01020,Vitis10g01021,Vitis10g01023,Vitis10g01025,Vitis10g01027 | 46 |
| PF03181 | Vitis04g00396,Vitis04g00399,Vitis04g00400,Vitis04g00401,Vitis04g00403,Vitis04g00404,Vitis04g00407,Vitis04g00408,Vitis04g00409,Vitis04g00410,Vitis04g00411 | 11 |
| PF03195 | Vitis13g00577,Vitis13g00579,Vitis13g00580,Vitis13g00583,Vitis13g00584,Vitis13g00585,Vitis13g00586,Vitis13g00587,Vitis13g00589,Vitis13g00590,Vitis13g00591,Vitis13g00594 | 12 |
| PF03492 | Vitis04g01471,Vitis04g01472,Vitis04g01475,Vitis04g01477,Vitis04g01482,Vitis04g01483,Vitis04g01484,Vitis04g01485,Vitis04g01487,Vitis04g01488,Vitis04g01489,Vitis04g01490,Vitis04g01491,Vitis17g00287,Vitis17g00288,Vitis17g00289,Vitis17g00290,Vitis17g00291 | 18 |
| PF03514 | Vitis13g00397,Vitis13g00398,Vitis13g00399,Vitis13g00400,Vitis13g00401,Vitis13g00402 | 6 |
| PF03547 | Vitis04g01997,Vitis04g01998,Vitis04g01999,Vitis04g02000,Vitis04g02003,Vitis04g02004 | 6 |
| PF03552 | Vitis02g00175,Vitis02g00176,Vitis02g00177,Vitis02g00178,Vitis02g00179,Vitis02g00181,Vitis02g00182,Vitis02g00183,Vitis02g00184,Vitis02g00185,Vitis02g00187,Vitis02g00188,Vitis02g00189,Vitis02g00190,Vitis02g00191,Vitis02g00194,Vitis02g00196,Vitis02g00197,Vitis02g00198,Vitis02g00199,Vitis10g00343,Vitis10g00344,Vitis10g00346,Vitis10g00347,Vitis10g00348,Vitis10g00349,Vitis10g00350,Vitis12g00531,Vitis12g00532,Vitis12g00533,Vitis12g00534,Vitis12g00535,Vitis12g00536,Vitis12g00537,Vitis12g00538,Vitis12g00539 | 36 |
| PF03754 | Vitis15g01253,Vitis15g01254,Vitis15g01255,Vitis15g01256,Vitis15g01257,Vitis15g01258,Vitis15g01259,Vitis15g01260,Vitis15g01261,Vitis15g01265,Vitis15g01267,Vitis15g01269,Vitis15g01274,Vitis16g01712,Vitis16g01713,Vitis16g01714,Vitis16g01717,Vitis16g01718 | 18 |
| PF03936 | Vitis18g02774,Vitis18g02775,Vitis18g02776,Vitis18g02777,Vitis18g02778,Vitis18g02782,Vitis18g02784,Vitis18g02789,Vitis18g02791,Vitis18g02799,Vitis18g02805 | 11 |
| PF04043 | Vitis01g00534,Vitis01g00535,Vitis01g00537,Vitis16g00884,Vitis16g00885,Vitis16g00896,Vitis16g00897,Vitis16g00899,Vitis16g00900,Vitis16g00901,Vitis16g00904,Vitis16g00907,Vitis16g00908 | 13 |
| PF04116 | Vitis15g00525,Vitis15g00526,Vitis15g00528,Vitis15g00529,Vitis15g00531,Vitis15g00532 | 6 |
| PF04398 | Vitis14g00195,Vitis14g00196,Vitis14g00197,Vitis14g00198,Vitis14g00199,Vitis14g00200,Vitis14g00201,Vitis14g00202,Vitis14g00203,Vitis14g00204,Vitis14g00205,Vitis14g00206,Vitis14g00207,Vitis14g00208 | 14 |
| PF04450 | Vitis03g00749,Vitis03g00750,Vitis03g00751,Vitis03g00752,Vitis03g00753,Vitis03g00755 | 6 |
| PF04578 | Vitis05g01915,Vitis05g01916,Vitis05g01917,Vitis05g01918,Vitis05g01920,Vitis05g01923,Vitis05g01926,Vitis05g01931,Vitis05g01946,Vitis09g00107,Vitis09g00108,Vitis09g00110,Vitis09g00122,Vitis09g00125,Vitis09g00132,Vitis09g00133 | 16 |
| PF04833 | Vitis17g00586,Vitis17g00587,Vitis17g00589,Vitis17g00590 | 4 |
| PF04885 | Vitis10g00104,Vitis10g00105,Vitis10g00109,Vitis10g00110,Vitis10g00111,Vitis10g00112 | 6 |
| PF05056 | Vitis13g00302,Vitis13g00303,Vitis13g00304,Vitis13g00305,Vitis13g00306,Vitis13g00307,Vitis13g00308,Vitis13g00309,Vitis13g00310,Vitis13g00311,Vitis13g00312,Vitis13g00313,Vitis13g00314,Vitis13g00315,Vitis13g00316,Vitis13g00317,Vitis13g00318 | 17 |
| PF05577 | Vitis06g01571,Vitis06g01572,Vitis06g01573,Vitis06g01574,Vitis06g01576,Vitis06g01580,Vitis06g01581,Vitis06g01582 | 8 |
| PF05617 | Vitis11g00680,Vitis11g00681,Vitis11g00682,Vitis11g00683 | 4 |
| PF05686 | Vitis15g01454,Vitis15g01455,Vitis15g01456,Vitis15g01457,Vitis15g01458,Vitis15g01459,Vitis15g01461,Vitis15g01462,Vitis15g01463 | 9 |
| PF05758 | Vitis02g01536,Vitis02g01537,Vitis02g01538,Vitis02g01539,Vitis02g01540,Vitis02g01541,Vitis02g01542 | 7 |
| PF06955 | Vitis11g01488,Vitis11g01489,Vitis11g01494,Vitis11g01495,Vitis11g01496,Vitis11g01498,Vitis11g01499,Vitis11g01500,Vitis11g01502 | 9 |
| PF07714 | Vitis07g02093,Vitis07g02095,Vitis07g02096,Vitis07g02097,Vitis07g02099,Vitis07g02102,Vitis07g02103,Vitis07g02106,Vitis07g02108,Vitis09g00281,Vitis09g00285,Vitis09g00286,Vitis09g00288,Vitis09g00290,Vitis09g00292,Vitis09g00293,Vitis09g00298,Vitis09g00299,Vitis09g00300,Vitis09g00302,Vitis09g00303,Vitis09g00305,Vitis09g00307,Vitis09g00312,Vitis09g00314,Vitis10g00414,Vitis10g00421,Vitis10g00422,Vitis10g00427,Vitis10g00429,Vitis10g00430,Vitis10g00432,Vitis10g00436,Vitis10g00437,Vitis10g00438,Vitis10g00442,Vitis10g00443,Vitis10g00445,Vitis10g00446,Vitis10g00447,Vitis10g00467,Vitis10g00534,Vitis10g00536,Vitis10g00537,Vitis10g00539,Vitis10g00542,Vitis10g00545,Vitis10g00547,Vitis10g00552,Vitis10g00554,Vitis10g00556,Vitis10g00557,Vitis10g00559,Vitis10g00562,Vitis10g00564,Vitis10g00570,Vitis10g00573,Vitis10g00574,Vitis10g00576 | 59 |
| PF07727 | Vitis09g00943,Vitis09g00944,Vitis09g00951,Vitis09g00979,Vitis10g01140,Vitis10g01146,Vitis10g01163,Vitis10g01164,Vitis10g01166,Vitis10g01170,Vitis10g01187,Vitis14g01046,Vitis14g01047,Vitis14g01061,Vitis14g01069,Vitis14g01070,Vitis14g01073,Vitis14g01074,Vitis14g01082,Vitis14g01098,Vitis15g00160,Vitis15g00175,Vitis15g00184,Vitis15g00185,Vitis15g00186,Vitis15g00188,Vitis15g00198,Vitis15g00200,Vitis15g00240,Vitis15g00247,Vitis15g00255,Vitis15g00266,Vitis15g00267,Vitis15g00269,Vitis15g00270,Vitis15g00272,Vitis15g00273,Vitis15g00275,Vitis15g00281,Vitis15g00282,Vitis15g00284,Vitis15g00285,Vitis15g00286,Vitis16g00357,Vitis16g00379,Vitis16g00380,Vitis16g00382,Vitis16g00383,Vitis16g00389,Vitis16g00396,Vitis16g00399,Vitis16g00400,Vitis16g00401,Vitis16g00405,Vitis17g01477,Vitis17g01502,Vitis17g01503,Vitis17g01506,Vitis18g02486,Vitis18g02487,Vitis18g02492,Vitis18g02502,Vitis18g02503,Vitis18g02505,Vitis18g02506,Vitis18g02520,Vitis18g02830,Vitis18g02831,Vitis18g02833,Vitis18g02834,Vitis18g02847,Vitis18g02860,Vitis19g00957,Vitis19g00966,Vitis19g00967,Vitis19g00974,Vitis19g00975,Vitis19g00981,Vitis19g00982,Vitis19g00983,Vitis19g00984 | 81 |
| PF07797 | Vitis15g00743,Vitis15g00744,Vitis15g00745,Vitis15g00746,Vitis15g00750,Vitis15g00752 | 6 |
| PF07859 | Vitis03g00630,Vitis03g00631,Vitis03g00632,Vitis03g00633,Vitis03g00634,Vitis03g00635,Vitis03g00636,Vitis03g00637,Vitis03g00638,Vitis03g00639,Vitis03g00640,Vitis03g00641,Vitis03g00642,Vitis04g00553,Vitis04g00554,Vitis04g00555,Vitis04g00556,Vitis04g00557,Vitis04g00558,Vitis04g00559,Vitis18g02927,Vitis18g02928,Vitis18g02929,Vitis18g02930,Vitis18g02932 | 25 |
| PF08263 | Vitis01g01693,Vitis01g01712,Vitis01g01714,Vitis01g01718,Vitis01g01720,Vitis01g01722,Vitis01g01733,Vitis01g01734,Vitis01g01739,Vitis01g01740,Vitis06g00046,Vitis06g00047,Vitis06g00048,Vitis06g00049,Vitis06g00051,Vitis06g00054,Vitis09g01470,Vitis09g01473,Vitis09g01474,Vitis09g01479,Vitis09g01484,Vitis09g01485,Vitis09g01489,Vitis09g01492,Vitis09g01494,Vitis09g01495,Vitis09g01497,Vitis09g01500,Vitis09g01503,Vitis14g01903,Vitis14g01904,Vitis14g01907,Vitis14g01913,Vitis14g01914,Vitis14g01919,Vitis14g01922,Vitis14g01924,Vitis14g01925,Vitis14g01926,Vitis14g01933,Vitis14g01934,Vitis14g01938,Vitis14g01941,Vitis14g01943,Vitis14g01947,Vitis14g01948,Vitis14g01951 | 47 |
| PF08718 | Vitis11g01366,Vitis11g01367,Vitis11g01368,Vitis11g01369,Vitis11g01373 | 5 |
| PF12609 | Vitis12g00821,Vitis12g00826,Vitis12g00828,Vitis12g00829,Vitis12g00830,Vitis12g00831,Vitis12g00832,Vitis12g00833,Vitis12g00834,Vitis12g00835,Vitis12g00836,Vitis12g00837,Vitis12g00838,Vitis12g00839,Vitis12g00841 | 15 |
| PF12796 | Vitis05g01772,Vitis05g01775,Vitis05g01783,Vitis05g01784,Vitis05g01786,Vitis05g01787,Vitis05g01788,Vitis05g01789,Vitis05g01793,Vitis05g01794,Vitis05g01798,Vitis05g01800,Vitis05g01801,Vitis05g01802,Vitis05g01803,Vitis05g01813,Vitis05g01819,Vitis05g01822,Vitis05g01826,Vitis05g01827,Vitis11g00148,Vitis11g00149,Vitis11g00151,Vitis11g00152,Vitis11g00154,Vitis11g00163,Vitis11g00165,Vitis11g00166,Vitis11g00169,Vitis11g00171,Vitis12g00415,Vitis12g00420,Vitis12g00421,Vitis12g00422,Vitis12g00425,Vitis12g00426,Vitis12g00427,Vitis12g00428,Vitis12g00431,Vitis12g00432,Vitis12g00439,Vitis12g00440,Vitis12g00441,Vitis12g00444,Vitis12g00445,Vitis12g00446,Vitis12g00447,Vitis12g00448 | 48 |
| PF13086 | Vitis16g01531,Vitis16g01532,Vitis16g01533,Vitis16g01534 | 4 |
| PF13243 | Vitis09g01592,Vitis09g01593,Vitis09g01595,Vitis09g01598,Vitis09g01600,Vitis09g01601,Vitis09g01602,Vitis09g01609,Vitis09g01613,Vitis09g01615,Vitis09g01616,Vitis09g01618,Vitis09g01620,Vitis09g01621,Vitis09g01626 | 15 |
| PF13249 | Vitis09g01592,Vitis09g01593,Vitis09g01595,Vitis09g01597,Vitis09g01600,Vitis09g01601,Vitis09g01602,Vitis09g01604,Vitis09g01614,Vitis09g01616,Vitis09g01618,Vitis09g01620,Vitis09g01621,Vitis09g01626 | 14 |
| PF13302 | Vitis14g00592,Vitis14g00594,Vitis14g00595,Vitis14g00597,Vitis14g00599 | 5 |
| PF13360 | Vitis14g02341,Vitis14g02342,Vitis14g02343,Vitis14g02344,Vitis14g02345,Vitis14g02348,Vitis14g02356 | 7 |
| PF13499 | Vitis01g01698,Vitis01g01699,Vitis01g01700,Vitis01g01701,Vitis01g01702,Vitis01g01704,Vitis01g01705,Vitis01g01706,Vitis12g00465,Vitis12g00466,Vitis12g00467,Vitis12g00469,Vitis12g00470,Vitis12g00471,Vitis12g00473,Vitis12g00475,Vitis12g00476,Vitis12g00477,Vitis12g00478,Vitis12g00479,Vitis12g00481 | 21 |
| PF13561 | Vitis06g01771,Vitis06g01772,Vitis06g01773,Vitis06g01774,Vitis17g00649,Vitis17g00650,Vitis17g00651,Vitis17g00653,Vitis17g00654 | 9 |
| PF13639 | Vitis01g00226,Vitis01g00227,Vitis01g00228,Vitis01g00229,Vitis01g00230,Vitis01g00232,Vitis07g02166,Vitis07g02167,Vitis07g02168,Vitis07g02169,Vitis07g02171 | 11 |
| PF13839 | Vitis14g01568,Vitis14g01569,Vitis14g01570,Vitis14g01571,Vitis14g01572 | 5 |
| PF13855 | Vitis01g01712,Vitis01g01713,Vitis01g01715,Vitis01g01718,Vitis01g01722,Vitis01g01733,Vitis01g01734,Vitis01g01739,Vitis01g01740,Vitis09g01458,Vitis09g01459,Vitis09g01460,Vitis09g01461,Vitis09g01462,Vitis09g01463,Vitis09g01464,Vitis09g01467,Vitis09g01473,Vitis09g01474,Vitis09g01479,Vitis09g01484,Vitis09g01485,Vitis09g01486,Vitis09g01487,Vitis09g01489,Vitis09g01491 | 26 |
| PF13912 | Vitis06g01311,Vitis06g01318,Vitis06g01319,Vitis06g01320,Vitis06g01321,Vitis06g01322,Vitis06g01323 | 7 |
| PF13947 | Vitis16g01602,Vitis16g01603,Vitis16g01605,Vitis16g01606,Vitis16g01611,Vitis16g01612,Vitis16g01615,Vitis16g01616,Vitis16g01618,Vitis16g01619,Vitis16g01620,Vitis16g01624,Vitis16g01625,Vitis16g01626,Vitis16g01628,Vitis16g01629,Vitis16g01630,Vitis16g01631,Vitis16g01632,Vitis16g01633,Vitis16g01636,Vitis16g01637,Vitis16g01638,Vitis16g01639,Vitis16g01643,Vitis16g01644,Vitis16g01647,Vitis16g01650,Vitis16g01651,Vitis16g01654,Vitis16g01655,Vitis16g01661,Vitis16g01662,Vitis16g01664,Vitis16g01667,Vitis16g01668,Vitis16g01669,Vitis16g01671,Vitis16g01672,Vitis16g01673,Vitis16g01674,Vitis16g01675,Vitis16g01676,Vitis16g01677,Vitis16g01678,Vitis16g01679,Vitis16g01680,Vitis16g01681,Vitis16g01682,Vitis16g01683,Vitis16g01685,Vitis16g01686,Vitis16g01688,Vitis16g01689,Vitis16g01692,Vitis16g01693,Vitis16g01694,Vitis16g01695,Vitis16g01697,Vitis16g01698,Vitis16g01699,Vitis16g01700,Vitis16g01701,Vitis16g01702,Vitis16g01703,Vitis16g01723,Vitis16g01724,Vitis16g01725,Vitis16g01726 | 69 |
| PF13962 | Vitis09g00886,Vitis09g00887,Vitis09g00890,Vitis09g00891,Vitis09g00901,Vitis09g00903,Vitis09g00904 | 7 |
| PF13966 | Vitis05g00996,Vitis05g01010,Vitis05g01011,Vitis05g01012,Vitis05g01018,Vitis05g01019,Vitis05g01025,Vitis05g01041,Vitis05g01045,Vitis09g00259,Vitis09g00260,Vitis09g00262 | 12 |
| PF13968 | Vitis05g01915,Vitis05g01916,Vitis05g01917,Vitis05g01918,Vitis05g01920,Vitis05g01923,Vitis05g01924,Vitis05g01931,Vitis05g01946,Vitis05g01950,Vitis09g00107,Vitis09g00110,Vitis09g00111,Vitis09g00113,Vitis09g00123,Vitis09g00125,Vitis09g00130,Vitis09g00132,Vitis09g00133,Vitis09g00134,Vitis09g00135 | 21 |
| PF14111 | Vitis13g01929,Vitis13g01940,Vitis13g01941,Vitis13g01943,Vitis13g01947 | 5 |
| PF14226 | Vitis02g00813,Vitis02g00814,Vitis02g00815,Vitis02g00823,Vitis02g00824,Vitis02g00825,Vitis02g00826,Vitis02g00827,Vitis02g00828,Vitis10g00998,Vitis10g00999,Vitis10g01001,Vitis10g01002,Vitis10g01003,Vitis10g01005,Vitis10g01006,Vitis10g01008,Vitis10g01010,Vitis10g01011,Vitis10g01013,Vitis10g01016,Vitis10g01017,Vitis10g01018,Vitis10g01019,Vitis10g01021,Vitis10g01023,Vitis10g01028 | 27 |
| PF14304 | Vitis17g00397,Vitis17g00400 | 2 |
| PF14327 | Vitis17g00396,Vitis17g00397,Vitis17g00399,Vitis17g00400 | 4 |
| PF14368 | Vitis13g01911,Vitis13g01912,Vitis13g01913,Vitis13g01914,Vitis13g01915 | 5 |
| PF14476 | Vitis10g00120,Vitis10g00121,Vitis10g00123,Vitis10g00124,Vitis10g00125,Vitis10g00126,Vitis10g00128 | 7 |
| PF14541 | Vitis02g01440,Vitis02g01441,Vitis02g01442,Vitis02g01443,Vitis02g01444,Vitis04g00733,Vitis04g00734,Vitis04g00735,Vitis04g00737,Vitis04g00738,Vitis04g00739,Vitis04g00743,Vitis04g00744,Vitis04g00747,Vitis04g00749,Vitis04g00751,Vitis04g00754,Vitis08g01317,Vitis08g01318,Vitis08g01319,Vitis08g01320,Vitis08g01321,Vitis08g01322,Vitis08g01324,Vitis08g01325 | 25 |
| PF14547 | Vitis02g00613,Vitis02g00614,Vitis02g00615,Vitis02g00616,Vitis02g00617 | 5 |
| PF14576 | Vitis14g02052,Vitis14g02055,Vitis14g02056,Vitis14g02057,Vitis14g02059,Vitis14g02063,Vitis14g02064 | 7 |
| PF16845 | Vitis10g00275,Vitis10g00276,Vitis10g00278,Vitis10g00279,Vitis10g00280,Vitis10g00282,Vitis10g00284 | 7 |
| PF17917 | Vitis08g00404,Vitis08g00411,Vitis08g00412,Vitis08g00424,Vitis08g00429,Vitis08g00431,Vitis08g00434,Vitis08g00437,Vitis08g00438,Vitis08g00439,Vitis08g00450,Vitis08g00453,Vitis08g00454,Vitis08g00458,Vitis08g00463 | 15 |
| PF18052 | Vitis01g00086,Vitis01g00087,Vitis01g00089,Vitis01g00093,Vitis01g00096,Vitis01g00097,Vitis01g00098,Vitis01g00112,Vitis13g01736,Vitis13g01737,Vitis13g01738,Vitis13g01739,Vitis13g01740,Vitis13g01741,Vitis13g01749,Vitis13g01750,Vitis13g01751,Vitis13g01752,Vitis13g01755,Vitis13g01757,Vitis13g01759,Vitis13g01760,Vitis13g01762,Vitis13g01763,Vitis13g01765,Vitis13g01771,Vitis13g01774,Vitis13g01776 | 28 |

| **Table S9 The genes located on the large heterozygous fragments shown in Figure 6A.** | | |
| --- | --- | --- |
| **Chr ID** | **Pos (bp)** | **Gene ID** |
| Chr01 | 1100000-1300000 | Vitis01g00129,Vitis01g00130,Vitis01g00131,Vitis01g00132,Vitis01g00133,Vitis01g00135,Vitis01g00136,Vitis01g00138,Vitis01g00139,Vitis01g00140,Vitis01g00141,Vitis01g00142,Vitis01g00144 |
| Chr02 | 5300000-7200000 | Vitis02g00512,Vitis02g00513,Vitis02g00514,Vitis02g00515,Vitis02g00516,Vitis02g00517,Vitis02g00518,Vitis02g00520,Vitis02g00521,Vitis02g00522,Vitis02g00523,Vitis02g00524,Vitis02g00525,Vitis02g00526,Vitis02g00527,Vitis02g00528,Vitis02g00529,Vitis02g00530,Vitis02g00531,Vitis02g00532,Vitis02g00533,Vitis02g00534,Vitis02g00535,Vitis02g00536,Vitis02g00537,Vitis02g00538,Vitis02g00539,Vitis02g00540,Vitis02g00541,Vitis02g00542,Vitis02g00543,Vitis02g00544,Vitis02g00546,Vitis02g00547,Vitis02g00548,Vitis02g00549,Vitis02g00551,Vitis02g00552,Vitis02g00553,Vitis02g00554,Vitis02g00555,Vitis02g00556,Vitis02g00557,Vitis02g00558,Vitis02g00560,Vitis02g00561,Vitis02g00564,Vitis02g00565,Vitis02g00566,Vitis02g00567,Vitis02g00568,Vitis02g00569,Vitis02g00570,Vitis02g00571,Vitis02g00572,Vitis02g00573,Vitis02g00575,Vitis02g00576,Vitis02g00577,Vitis02g00578,Vitis02g00579,Vitis02g00580,Vitis02g00581,Vitis02g00582,Vitis02g00583,Vitis02g00587,Vitis02g00588,Vitis02g00589,Vitis02g00591,Vitis02g00592,Vitis02g00593,Vitis02g00594,Vitis02g00595,Vitis02g00599,Vitis02g00600,Vitis02g00601,Vitis02g00602,Vitis02g00603,Vitis02g00606,Vitis02g00607,Vitis02g00608,Vitis02g00609,Vitis02g00611,Vitis02g00614,Vitis02g00618,Vitis02g00633,Vitis02g00635,Vitis02g00636,Vitis02g00637,Vitis02g00638,Vitis02g00639,Vitis02g00640,Vitis02g00641,Vitis02g00642,Vitis02g00643,Vitis02g00644,Vitis02g00645,Vitis02g00646,Vitis02g00647,Vitis02g00648,Vitis02g00649,Vitis02g00650,Vitis02g00651,Vitis02g00652,Vitis02g00653,Vitis02g00654,Vitis02g00655,Vitis02g00656,Vitis02g00657,Vitis02g00658,Vitis02g00659,Vitis02g00660,Vitis02g00661,Vitis02g00662,Vitis02g00663,Vitis02g00664,Vitis02g00665,Vitis02g00667,Vitis02g00668,Vitis02g00669,Vitis02g00670,Vitis02g00671,Vitis02g00672,Vitis02g00673,Vitis02g00674,Vitis02g00675,Vitis02g00676,Vitis02g00677,Vitis02g00678,Vitis02g00679,Vitis02g00681,Vitis02g00682,Vitis02g00683,Vitis02g00684,Vitis02g00685,Vitis02g00686,Vitis02g00687,Vitis02g00688,Vitis02g00689,Vitis02g00691,Vitis02g00692,Vitis02g00693,Vitis02g00694,Vitis02g00695,Vitis02g00696,Vitis02g00697,Vitis02g00699,Vitis02g00700,Vitis02g00702,Vitis02g00703,Vitis02g00704,Vitis02g00705,Vitis02g00706,Vitis02g00707,Vitis02g00708,Vitis02g00709,Vitis02g00710,Vitis02g00711,Vitis02g00715,Vitis02g00716,Vitis02g00717,Vitis02g00718,Vitis02g00719,Vitis02g00720,Vitis02g00721,Vitis02g00722,Vitis02g00723,Vitis02g00724,Vitis02g00725,Vitis02g00726,Vitis02g00728,Vitis02g00729,Vitis02g00730,Vitis02g00731,Vitis02g00732,Vitis02g00733,Vitis02g00735,Vitis02g00736,Vitis02g00737,Vitis02g00739,Vitis02g00740,Vitis02g00741,Vitis02g00742,Vitis02g00743,Vitis02g00744,Vitis02g00748,Vitis02g00749,Vitis02g00750,Vitis02g00751,Vitis02g00752,Vitis02g00753,Vitis02g00754,Vitis02g00755,Vitis02g00756,Vitis02g00757,Vitis02g00758,Vitis02g00759,Vitis02g00762,Vitis02g00763,Vitis02g00764,Vitis02g00766,Vitis02g00767,Vitis02g00768,Vitis02g00769,Vitis02g00770,Vitis02g00771,Vitis02g00772,Vitis02g00773,Vitis02g00774,Vitis02g00775,Vitis02g00776,Vitis02g00779,Vitis02g00780,Vitis02g00781,Vitis02g00782,Vitis02g00783,Vitis02g00784,Vitis02g00785 |
| Chr03 | 9400000-9900000 | Vitis03g01027,Vitis03g01028,Vitis03g01029,Vitis03g01030,Vitis03g01031,Vitis03g01032,Vitis03g01033,Vitis03g01035,Vitis03g01036,Vitis03g01039,Vitis03g01040,Vitis03g01041,Vitis03g01042 |
| Chr04 | 21800000-22900000 | Vitis04g01655,Vitis04g01656,Vitis04g01657,Vitis04g01658,Vitis04g01659,Vitis04g01660,Vitis04g01661,Vitis04g01662,Vitis04g01663,Vitis04g01664,Vitis04g01665,Vitis04g01666,Vitis04g01667,Vitis04g01668,Vitis04g01669,Vitis04g01670,Vitis04g01671,Vitis04g01672,Vitis04g01673,Vitis04g01676,Vitis04g01677,Vitis04g01678,Vitis04g01679,Vitis04g01680,Vitis04g01681,Vitis04g01682,Vitis04g01683,Vitis04g01684,Vitis04g01685,Vitis04g01686,Vitis04g01687,Vitis04g01688,Vitis04g01689,Vitis04g01690,Vitis04g01691,Vitis04g01692,Vitis04g01693,Vitis04g01694,Vitis04g01695,Vitis04g01696,Vitis04g01697,Vitis04g01698,Vitis04g01699,Vitis04g01700,Vitis04g01701,Vitis04g01702,Vitis04g01703,Vitis04g01704,Vitis04g01705,Vitis04g01708,Vitis04g01709,Vitis04g01710,Vitis04g01711,Vitis04g01713,Vitis04g01714,Vitis04g01715,Vitis04g01717,Vitis04g01718,Vitis04g01719,Vitis04g01720,Vitis04g01721,Vitis04g01722,Vitis04g01723,Vitis04g01724,Vitis04g01725,Vitis04g01726,Vitis04g01729,Vitis04g01730,Vitis04g01731,Vitis04g01732,Vitis04g01733,Vitis04g01734,Vitis04g01735,Vitis04g01736,Vitis04g01737,Vitis04g01738,Vitis04g01739,Vitis04g01741,Vitis04g01742,Vitis04g01743,Vitis04g01744,Vitis04g01745,Vitis04g01747,Vitis04g01748,Vitis04g01749,Vitis04g01751,Vitis04g01752,Vitis04g01753,Vitis04g01754,Vitis04g01755,Vitis04g01756,Vitis04g01757,Vitis04g01758,Vitis04g01759,Vitis04g01760,Vitis04g01761,Vitis04g01762,Vitis04g01763,Vitis04g01764,Vitis04g01765,Vitis04g01766,Vitis04g01768,Vitis04g01769,Vitis04g01770,Vitis04g01771,Vitis04g01772,Vitis04g01773,Vitis04g01774,Vitis04g01775,Vitis04g01776,Vitis04g01777,Vitis04g01778,Vitis04g01784 |
| Chr07 | 15300000-26200000 | Vitis07g01178,Vitis07g01179,Vitis07g01180,Vitis07g01183,Vitis07g01184,Vitis07g01186,Vitis07g01187,Vitis07g01188,Vitis07g01189,Vitis07g01190,Vitis07g01193,Vitis07g01196,Vitis07g01197,Vitis07g01198,Vitis07g01199,Vitis07g01201,Vitis07g01202,Vitis07g01204,Vitis07g01205,Vitis07g01206,Vitis07g01208,Vitis07g01209,Vitis07g01210,Vitis07g01211,Vitis07g01212,Vitis07g01213,Vitis07g01214,Vitis07g01219,Vitis07g01220,Vitis07g01221,Vitis07g01222,Vitis07g01223,Vitis07g01224,Vitis07g01226,Vitis07g01227,Vitis07g01228,Vitis07g01229,Vitis07g01230,Vitis07g01231,Vitis07g01232,Vitis07g01233,Vitis07g01234,Vitis07g01235,Vitis07g01236,Vitis07g01237,Vitis07g01238,Vitis07g01239,Vitis07g01240,Vitis07g01241,Vitis07g01244,Vitis07g01248,Vitis07g01253,Vitis07g01254,Vitis07g01255,Vitis07g01256,Vitis07g01258,Vitis07g01259,Vitis07g01262,Vitis07g01263,Vitis07g01264,Vitis07g01265,Vitis07g01266,Vitis07g01267,Vitis07g01268,Vitis07g01269,Vitis07g01270,Vitis07g01271,Vitis07g01272,Vitis07g01273,Vitis07g01277,Vitis07g01280,Vitis07g01283,Vitis07g01293,Vitis07g01294,Vitis07g01295,Vitis07g01299,Vitis07g01306,Vitis07g01309,Vitis07g01310,Vitis07g01311,Vitis07g01312,Vitis07g01313,Vitis07g01316,Vitis07g01317,Vitis07g01319,Vitis07g01320,Vitis07g01321,Vitis07g01322,Vitis07g01323,Vitis07g01324,Vitis07g01325,Vitis07g01327,Vitis07g01328,Vitis07g01329,Vitis07g01330,Vitis07g01331,Vitis07g01332,Vitis07g01333,Vitis07g01334,Vitis07g01339,Vitis07g01340,Vitis07g01341,Vitis07g01342,Vitis07g01343,Vitis07g01345,Vitis07g01347,Vitis07g01349,Vitis07g01350,Vitis07g01351,Vitis07g01352,Vitis07g01353,Vitis07g01356,Vitis07g01357,Vitis07g01358,Vitis07g01359,Vitis07g01360,Vitis07g01362,Vitis07g01363,Vitis07g01364,Vitis07g01365,Vitis07g01366,Vitis07g01368,Vitis07g01369,Vitis07g01370,Vitis07g01371,Vitis07g01373,Vitis07g01374,Vitis07g01375,Vitis07g01379,Vitis07g01380,Vitis07g01381,Vitis07g01382,Vitis07g01383,Vitis07g01386,Vitis07g01387,Vitis07g01388,Vitis07g01389,Vitis07g01390,Vitis07g01391,Vitis07g01392,Vitis07g01393,Vitis07g01394,Vitis07g01395,Vitis07g01399,Vitis07g01400,Vitis07g01401,Vitis07g01402,Vitis07g01403,Vitis07g01404,Vitis07g01407,Vitis07g01409,Vitis07g01410,Vitis07g01411,Vitis07g01412,Vitis07g01414,Vitis07g01415,Vitis07g01416,Vitis07g01417,Vitis07g01418,Vitis07g01419,Vitis07g01420,Vitis07g01423,Vitis07g01425,Vitis07g01426,Vitis07g01427,Vitis07g01428,Vitis07g01429,Vitis07g01430,Vitis07g01431,Vitis07g01432,Vitis07g01433,Vitis07g01434,Vitis07g01435,Vitis07g01436,Vitis07g01437,Vitis07g01442,Vitis07g01443,Vitis07g01444,Vitis07g01445,Vitis07g01446,Vitis07g01447,Vitis07g01451,Vitis07g01452,Vitis07g01453,Vitis07g01454,Vitis07g01455,Vitis07g01456,Vitis07g01457,Vitis07g01458,Vitis07g01459,Vitis07g01460,Vitis07g01461,Vitis07g01462,Vitis07g01463,Vitis07g01464,Vitis07g01465,Vitis07g01466,Vitis07g01467,Vitis07g01468,Vitis07g01469,Vitis07g01471,Vitis07g01472,Vitis07g01473,Vitis07g01474,Vitis07g01475,Vitis07g01476,Vitis07g01477,Vitis07g01478,Vitis07g01479,Vitis07g01480,Vitis07g01481,Vitis07g01482,Vitis07g01483,Vitis07g01484,Vitis07g01485,Vitis07g01486,Vitis07g01487,Vitis07g01488,Vitis07g01489,Vitis07g01490,Vitis07g01491,Vitis07g01492,Vitis07g01499,Vitis07g01500,Vitis07g01501,Vitis07g01502,Vitis07g01503,Vitis07g01504,Vitis07g01505,Vitis07g01506,Vitis07g01507,Vitis07g01508,Vitis07g01509,Vitis07g01510,Vitis07g01511,Vitis07g01512,Vitis07g01513,Vitis07g01514,Vitis07g01515,Vitis07g01516,Vitis07g01517,Vitis07g01518,Vitis07g01519,Vitis07g01520,Vitis07g01521,Vitis07g01522,Vitis07g01523,Vitis07g01524,Vitis07g01525,Vitis07g01526,Vitis07g01527,Vitis07g01528,Vitis07g01531,Vitis07g01533,Vitis07g01534,Vitis07g01535,Vitis07g01536,Vitis07g01537,Vitis07g01538,Vitis07g01539,Vitis07g01540,Vitis07g01541,Vitis07g01542,Vitis07g01543,Vitis07g01544,Vitis07g01545,Vitis07g01546,Vitis07g01547,Vitis07g01549,Vitis07g01550,Vitis07g01551,Vitis07g01552,Vitis07g01553,Vitis07g01554,Vitis07g01557,Vitis07g01558,Vitis07g01559,Vitis07g01560,Vitis07g01562,Vitis07g01563,Vitis07g01564,Vitis07g01565,Vitis07g01567,Vitis07g01568,Vitis07g01569,Vitis07g01570,Vitis07g01571,Vitis07g01572,Vitis07g01573,Vitis07g01574,Vitis07g01575,Vitis07g01576,Vitis07g01577,Vitis07g01579,Vitis07g01580,Vitis07g01581,Vitis07g01585,Vitis07g01586,Vitis07g01587,Vitis07g01588,Vitis07g01589,Vitis07g01590,Vitis07g01591,Vitis07g01592,Vitis07g01593,Vitis07g01594,Vitis07g01595,Vitis07g01596,Vitis07g01597,Vitis07g01598,Vitis07g01599,Vitis07g01600,Vitis07g01601,Vitis07g01602,Vitis07g01603,Vitis07g01604,Vitis07g01605,Vitis07g01606,Vitis07g01607,Vitis07g01608,Vitis07g01609,Vitis07g01611,Vitis07g01612,Vitis07g01613,Vitis07g01614,Vitis07g01615,Vitis07g01616,Vitis07g01617,Vitis07g01618,Vitis07g01619,Vitis07g01620,Vitis07g01622,Vitis07g01623,Vitis07g01624,Vitis07g01626,Vitis07g01627,Vitis07g01628,Vitis07g01629,Vitis07g01630,Vitis07g01631,Vitis07g01632,Vitis07g01633,Vitis07g01634,Vitis07g01635,Vitis07g01636,Vitis07g01637,Vitis07g01638,Vitis07g01639,Vitis07g01640,Vitis07g01641,Vitis07g01642,Vitis07g01643,Vitis07g01644,Vitis07g01645,Vitis07g01646,Vitis07g01647,Vitis07g01648,Vitis07g01649,Vitis07g01650,Vitis07g01651,Vitis07g01652,Vitis07g01653,Vitis07g01654,Vitis07g01655,Vitis07g01656,Vitis07g01657,Vitis07g01658,Vitis07g01659,Vitis07g01660,Vitis07g01661,Vitis07g01662,Vitis07g01663,Vitis07g01664,Vitis07g01665,Vitis07g01667,Vitis07g01668,Vitis07g01669,Vitis07g01670,Vitis07g01671,Vitis07g01672,Vitis07g01673,Vitis07g01674,Vitis07g01675,Vitis07g01676,Vitis07g01677,Vitis07g01678,Vitis07g01679,Vitis07g01680,Vitis07g01681,Vitis07g01682,Vitis07g01683,Vitis07g01684,Vitis07g01685,Vitis07g01686,Vitis07g01687,Vitis07g01688,Vitis07g01689,Vitis07g01690,Vitis07g01691,Vitis07g01692,Vitis07g01693,Vitis07g01694,Vitis07g01695,Vitis07g01696,Vitis07g01697,Vitis07g01698,Vitis07g01699,Vitis07g01700,Vitis07g01701,Vitis07g01702,Vitis07g01703,Vitis07g01704,Vitis07g01705,Vitis07g01706,Vitis07g01708,Vitis07g01709,Vitis07g01710,Vitis07g01711,Vitis07g01712,Vitis07g01714,Vitis07g01715,Vitis07g01716,Vitis07g01717,Vitis07g01718,Vitis07g01719,Vitis07g01720,Vitis07g01721,Vitis07g01722,Vitis07g01723,Vitis07g01724,Vitis07g01728,Vitis07g01729,Vitis07g01730,Vitis07g01731,Vitis07g01732,Vitis07g01733,Vitis07g01734,Vitis07g01735,Vitis07g01736,Vitis07g01737,Vitis07g01738,Vitis07g01739,Vitis07g01740,Vitis07g01742,Vitis07g01743,Vitis07g01744,Vitis07g01745,Vitis07g01746,Vitis07g01747,Vitis07g01748,Vitis07g01749,Vitis07g01750,Vitis07g01751,Vitis07g01753,Vitis07g01755,Vitis07g01756,Vitis07g01757,Vitis07g01758,Vitis07g01759,Vitis07g01761,Vitis07g01762,Vitis07g01763,Vitis07g01764,Vitis07g01765,Vitis07g01767,Vitis07g01768,Vitis07g01769,Vitis07g01770,Vitis07g01771,Vitis07g01772,Vitis07g01773,Vitis07g01774,Vitis07g01775,Vitis07g01778,Vitis07g01779,Vitis07g01780,Vitis07g01781,Vitis07g01782,Vitis07g01783,Vitis07g01784,Vitis07g01785,Vitis07g01786,Vitis07g01787,Vitis07g01788,Vitis07g01790,Vitis07g01791,Vitis07g01792,Vitis07g01793,Vitis07g01794,Vitis07g01796,Vitis07g01797,Vitis07g01798,Vitis07g01799,Vitis07g01800,Vitis07g01801,Vitis07g01802,Vitis07g01804,Vitis07g01805,Vitis07g01806,Vitis07g01807,Vitis07g01808,Vitis07g01809,Vitis07g01810,Vitis07g01811,Vitis07g01812,Vitis07g01814,Vitis07g01815,Vitis07g01816,Vitis07g01817,Vitis07g01818,Vitis07g01819,Vitis07g01820,Vitis07g01821,Vitis07g01822,Vitis07g01823,Vitis07g01824,Vitis07g01825,Vitis07g01826,Vitis07g01827,Vitis07g01828,Vitis07g01829,Vitis07g01830,Vitis07g01831,Vitis07g01832,Vitis07g01833,Vitis07g01836,Vitis07g01837,Vitis07g01838,Vitis07g01839,Vitis07g01840,Vitis07g01841,Vitis07g01842,Vitis07g01843,Vitis07g01844,Vitis07g01846,Vitis07g01847,Vitis07g01848,Vitis07g01850,Vitis07g01851,Vitis07g01852,Vitis07g01853,Vitis07g01854,Vitis07g01855,Vitis07g01856,Vitis07g01857,Vitis07g01858,Vitis07g01859,Vitis07g01860,Vitis07g01861,Vitis07g01862,Vitis07g01863,Vitis07g01864,Vitis07g01865,Vitis07g01867,Vitis07g01868,Vitis07g01869,Vitis07g01870,Vitis07g01871,Vitis07g01872,Vitis07g01873,Vitis07g01874,Vitis07g01875,Vitis07g01876,Vitis07g01877,Vitis07g01878,Vitis07g01879,Vitis07g01880,Vitis07g01881,Vitis07g01882,Vitis07g01883,Vitis07g01884,Vitis07g01885,Vitis07g01886,Vitis07g01887,Vitis07g01888,Vitis07g01889,Vitis07g01890,Vitis07g01891,Vitis07g01892,Vitis07g01893,Vitis07g01894,Vitis07g01895,Vitis07g01896,Vitis07g01897,Vitis07g01898,Vitis07g01899,Vitis07g01900,Vitis07g01901,Vitis07g01902,Vitis07g01903,Vitis07g01904,Vitis07g01905,Vitis07g01906,Vitis07g01907,Vitis07g01908,Vitis07g01909,Vitis07g01910,Vitis07g01911,Vitis07g01912,Vitis07g01913,Vitis07g01914,Vitis07g01915,Vitis07g01916,Vitis07g01917,Vitis07g01918,Vitis07g01919,Vitis07g01920,Vitis07g01921,Vitis07g01922,Vitis07g01923,Vitis07g01924,Vitis07g01926,Vitis07g01927,Vitis07g01928,Vitis07g01929,Vitis07g01930,Vitis07g01932,Vitis07g01934,Vitis07g01936,Vitis07g01937,Vitis07g01938,Vitis07g01939,Vitis07g01940,Vitis07g01941,Vitis07g01942,Vitis07g01943,Vitis07g01945,Vitis07g01946,Vitis07g01947,Vitis07g01948,Vitis07g01949,Vitis07g01950,Vitis07g01951,Vitis07g01953,Vitis07g01954,Vitis07g01955,Vitis07g01956,Vitis07g01957,Vitis07g01958,Vitis07g01964,Vitis07g01965,Vitis07g01966,Vitis07g01967,Vitis07g01968,Vitis07g01969,Vitis07g01970,Vitis07g01971,Vitis07g01972,Vitis07g01973,Vitis07g01975,Vitis07g01976,Vitis07g01977,Vitis07g01978,Vitis07g01979,Vitis07g01980,Vitis07g01981,Vitis07g01982,Vitis07g01984,Vitis07g01985,Vitis07g01986,Vitis07g01987,Vitis07g01989,Vitis07g01992,Vitis07g01993,Vitis07g01994,Vitis07g01995,Vitis07g01996,Vitis07g01997,Vitis07g01998,Vitis07g01999,Vitis07g02000,Vitis07g02001,Vitis07g02002,Vitis07g02004,Vitis07g02005,Vitis07g02006,Vitis07g02007,Vitis07g02008,Vitis07g02009,Vitis07g02010,Vitis07g02011,Vitis07g02012,Vitis07g02013,Vitis07g02014,Vitis07g02015,Vitis07g02017,Vitis07g02018,Vitis07g02019,Vitis07g02020,Vitis07g02021,Vitis07g02022,Vitis07g02023,Vitis07g02024,Vitis07g02025,Vitis07g02026,Vitis07g02027,Vitis07g02028 |
| Chr10 | 700000-6500000 | Vitis10g00117,Vitis10g00118,Vitis10g00119,Vitis10g00120,Vitis10g00124,Vitis10g00125,Vitis10g00126,Vitis10g00127,Vitis10g00129,Vitis10g00132,Vitis10g00133,Vitis10g00138,Vitis10g00139,Vitis10g00140,Vitis10g00142,Vitis10g00143,Vitis10g00144,Vitis10g00145,Vitis10g00146,Vitis10g00147,Vitis10g00149,Vitis10g00150,Vitis10g00151,Vitis10g00152,Vitis10g00153,Vitis10g00154,Vitis10g00156,Vitis10g00157,Vitis10g00158,Vitis10g00160,Vitis10g00161,Vitis10g00162,Vitis10g00163,Vitis10g00164,Vitis10g00165,Vitis10g00166,Vitis10g00167,Vitis10g00168,Vitis10g00169,Vitis10g00170,Vitis10g00171,Vitis10g00172,Vitis10g00173,Vitis10g00174,Vitis10g00176,Vitis10g00177,Vitis10g00178,Vitis10g00179,Vitis10g00180,Vitis10g00181,Vitis10g00182,Vitis10g00183,Vitis10g00184,Vitis10g00185,Vitis10g00186,Vitis10g00187,Vitis10g00188,Vitis10g00189,Vitis10g00190,Vitis10g00191,Vitis10g00192,Vitis10g00193,Vitis10g00194,Vitis10g00195,Vitis10g00196,Vitis10g00197,Vitis10g00198,Vitis10g00199,Vitis10g00204,Vitis10g00205,Vitis10g00207,Vitis10g00208,Vitis10g00210,Vitis10g00211,Vitis10g00213,Vitis10g00214,Vitis10g00215,Vitis10g00216,Vitis10g00217,Vitis10g00218,Vitis10g00220,Vitis10g00221,Vitis10g00222,Vitis10g00223,Vitis10g00224,Vitis10g00225,Vitis10g00226,Vitis10g00227,Vitis10g00229,Vitis10g00230,Vitis10g00231,Vitis10g00232,Vitis10g00233,Vitis10g00234,Vitis10g00236,Vitis10g00237,Vitis10g00239,Vitis10g00240,Vitis10g00241,Vitis10g00242,Vitis10g00243,Vitis10g00244,Vitis10g00245,Vitis10g00246,Vitis10g00247,Vitis10g00248,Vitis10g00249,Vitis10g00251,Vitis10g00252,Vitis10g00253,Vitis10g00254,Vitis10g00255,Vitis10g00256,Vitis10g00257,Vitis10g00258,Vitis10g00259,Vitis10g00260,Vitis10g00261,Vitis10g00262,Vitis10g00264,Vitis10g00265,Vitis10g00266,Vitis10g00267,Vitis10g00268,Vitis10g00269,Vitis10g00270,Vitis10g00271,Vitis10g00272,Vitis10g00273,Vitis10g00274,Vitis10g00275,Vitis10g00277,Vitis10g00278,Vitis10g00279,Vitis10g00281,Vitis10g00283,Vitis10g00287,Vitis10g00288,Vitis10g00289,Vitis10g00290,Vitis10g00291,Vitis10g00292,Vitis10g00293,Vitis10g00294,Vitis10g00295,Vitis10g00296,Vitis10g00297,Vitis10g00298,Vitis10g00299,Vitis10g00300,Vitis10g00301,Vitis10g00302,Vitis10g00303,Vitis10g00304,Vitis10g00305,Vitis10g00306,Vitis10g00307,Vitis10g00308,Vitis10g00311,Vitis10g00312,Vitis10g00313,Vitis10g00315,Vitis10g00316,Vitis10g00319,Vitis10g00320,Vitis10g00321,Vitis10g00322,Vitis10g00323,Vitis10g00324,Vitis10g00326,Vitis10g00327,Vitis10g00328,Vitis10g00329,Vitis10g00330,Vitis10g00331,Vitis10g00332,Vitis10g00333,Vitis10g00334,Vitis10g00335,Vitis10g00336,Vitis10g00337,Vitis10g00338,Vitis10g00339,Vitis10g00340,Vitis10g00341,Vitis10g00342,Vitis10g00343,Vitis10g00344,Vitis10g00345,Vitis10g00346,Vitis10g00347,Vitis10g00348,Vitis10g00349,Vitis10g00350,Vitis10g00351,Vitis10g00352,Vitis10g00353,Vitis10g00354,Vitis10g00355,Vitis10g00356,Vitis10g00357,Vitis10g00358,Vitis10g00359,Vitis10g00360,Vitis10g00361,Vitis10g00362,Vitis10g00363,Vitis10g00364,Vitis10g00365,Vitis10g00366,Vitis10g00367,Vitis10g00368,Vitis10g00369,Vitis10g00371,Vitis10g00372,Vitis10g00373,Vitis10g00374,Vitis10g00375,Vitis10g00376,Vitis10g00377,Vitis10g00378,Vitis10g00379,Vitis10g00380,Vitis10g00381,Vitis10g00382,Vitis10g00383,Vitis10g00384,Vitis10g00385,Vitis10g00386,Vitis10g00387,Vitis10g00388,Vitis10g00389,Vitis10g00390,Vitis10g00391,Vitis10g00392,Vitis10g00393,Vitis10g00394,Vitis10g00395,Vitis10g00396,Vitis10g00397,Vitis10g00398,Vitis10g00399,Vitis10g00400,Vitis10g00401,Vitis10g00402,Vitis10g00403,Vitis10g00404,Vitis10g00405,Vitis10g00406,Vitis10g00408,Vitis10g00409,Vitis10g00414,Vitis10g00415,Vitis10g00416,Vitis10g00417,Vitis10g00418,Vitis10g00419,Vitis10g00420,Vitis10g00421,Vitis10g00422,Vitis10g00423,Vitis10g00424,Vitis10g00425,Vitis10g00426,Vitis10g00427,Vitis10g00429,Vitis10g00430,Vitis10g00432,Vitis10g00433,Vitis10g00434,Vitis10g00435,Vitis10g00436,Vitis10g00437,Vitis10g00438,Vitis10g00439,Vitis10g00440,Vitis10g00441,Vitis10g00442,Vitis10g00443,Vitis10g00444,Vitis10g00445,Vitis10g00446,Vitis10g00447,Vitis10g00448,Vitis10g00449,Vitis10g00450,Vitis10g00451,Vitis10g00452,Vitis10g00455,Vitis10g00456,Vitis10g00457,Vitis10g00458,Vitis10g00459,Vitis10g00460,Vitis10g00461,Vitis10g00462,Vitis10g00463,Vitis10g00464,Vitis10g00465,Vitis10g00466,Vitis10g00467,Vitis10g00468,Vitis10g00469,Vitis10g00470,Vitis10g00471,Vitis10g00472,Vitis10g00473,Vitis10g00474,Vitis10g00475,Vitis10g00476,Vitis10g00477,Vitis10g00480,Vitis10g00481,Vitis10g00485,Vitis10g00492,Vitis10g00493,Vitis10g00494,Vitis10g00495,Vitis10g00498,Vitis10g00499,Vitis10g00500,Vitis10g00501,Vitis10g00502,Vitis10g00503,Vitis10g00504,Vitis10g00505,Vitis10g00507,Vitis10g00508,Vitis10g00509,Vitis10g00510,Vitis10g00511,Vitis10g00512,Vitis10g00513,Vitis10g00517,Vitis10g00518,Vitis10g00522,Vitis10g00523,Vitis10g00524,Vitis10g00527,Vitis10g00539,Vitis10g00540,Vitis10g00541,Vitis10g00542,Vitis10g00543,Vitis10g00544,Vitis10g00545,Vitis10g00546,Vitis10g00547,Vitis10g00548,Vitis10g00552,Vitis10g00553,Vitis10g00554,Vitis10g00555,Vitis10g00556,Vitis10g00557,Vitis10g00558,Vitis10g00559,Vitis10g00562,Vitis10g00563,Vitis10g00564,Vitis10g00565,Vitis10g00566,Vitis10g00567,Vitis10g00568,Vitis10g00569,Vitis10g00570,Vitis10g00571,Vitis10g00572,Vitis10g00573,Vitis10g00574,Vitis10g00576,Vitis10g00577,Vitis10g00578,Vitis10g00579,Vitis10g00580,Vitis10g00581,Vitis10g00582,Vitis10g00583,Vitis10g00584,Vitis10g00585,Vitis10g00586,Vitis10g00587,Vitis10g00588,Vitis10g00589,Vitis10g00590,Vitis10g00593,Vitis10g00594,Vitis10g00595,Vitis10g00596,Vitis10g00598,Vitis10g00599,Vitis10g00600,Vitis10g00601,Vitis10g00602,Vitis10g00603,Vitis10g00604,Vitis10g00605,Vitis10g00606,Vitis10g00608,Vitis10g00609,Vitis10g00610,Vitis10g00611,Vitis10g00612,Vitis10g00613,Vitis10g00614,Vitis10g00615,Vitis10g00616,Vitis10g00617,Vitis10g00618,Vitis10g00619,Vitis10g00620,Vitis10g00621,Vitis10g00622,Vitis10g00623,Vitis10g00624,Vitis10g00625,Vitis10g00626,Vitis10g00627,Vitis10g00628,Vitis10g00629,Vitis10g00630,Vitis10g00631,Vitis10g00632,Vitis10g00633,Vitis10g00634,Vitis10g00635,Vitis10g00636,Vitis10g00637,Vitis10g00638,Vitis10g00642,Vitis10g00643,Vitis10g00644,Vitis10g00645,Vitis10g00646,Vitis10g00647,Vitis10g00648,Vitis10g00649,Vitis10g00650,Vitis10g00651,Vitis10g00652,Vitis10g00653,Vitis10g00654,Vitis10g00655,Vitis10g00656,Vitis10g00657,Vitis10g00658,Vitis10g00659,Vitis10g00660,Vitis10g00661,Vitis10g00662,Vitis10g00663,Vitis10g00664,Vitis10g00665,Vitis10g00666,Vitis10g00667,Vitis10g00668,Vitis10g00669,Vitis10g00670,Vitis10g00671,Vitis10g00672,Vitis10g00673,Vitis10g00674,Vitis10g00675,Vitis10g00678,Vitis10g00679,Vitis10g00680,Vitis10g00681,Vitis10g00682,Vitis10g00683,Vitis10g00684,Vitis10g00685,Vitis10g00686,Vitis10g00687,Vitis10g00688,Vitis10g00689,Vitis10g00690,Vitis10g00691,Vitis10g00692,Vitis10g00693,Vitis10g00694,Vitis10g00695,Vitis10g00696,Vitis10g00697,Vitis10g00698,Vitis10g00699,Vitis10g00700,Vitis10g00701,Vitis10g00702,Vitis10g00703,Vitis10g00704,Vitis10g00705,Vitis10g00707,Vitis10g00709,Vitis10g00710,Vitis10g00711,Vitis10g00712,Vitis10g00713,Vitis10g00714,Vitis10g00715,Vitis10g00716,Vitis10g00717,Vitis10g00718,Vitis10g00719,Vitis10g00720,Vitis10g00721,Vitis10g00722,Vitis10g00723,Vitis10g00724,Vitis10g00725,Vitis10g00726,Vitis10g00727,Vitis10g00728,Vitis10g00729,Vitis10g00730,Vitis10g00732,Vitis10g00733,Vitis10g00734,Vitis10g00735,Vitis10g00737,Vitis10g00738,Vitis10g00739,Vitis10g00740,Vitis10g00741,Vitis10g00744, |
| Chr10 | 17600000-18300000 | Vitis10g01572,Vitis10g01573,Vitis10g01574,Vitis10g01575,Vitis10g01576,Vitis10g01577,Vitis10g01578,Vitis10g01579,Vitis10g01580,Vitis10g01581,Vitis10g01582,Vitis10g01583,Vitis10g01584,Vitis10g01585,Vitis10g01586,Vitis10g01587,Vitis10g01589,Vitis10g01591,Vitis10g01594,Vitis10g01595,Vitis10g01596,Vitis10g01597,Vitis10g01598,Vitis10g01599,Vitis10g01600,Vitis10g01601,Vitis10g01602,Vitis10g01603,Vitis10g01604,Vitis10g01605,Vitis10g01606,Vitis10g01608,Vitis10g01609,Vitis10g01610,Vitis10g01611,Vitis10g01612,Vitis10g01613,Vitis10g01615,Vitis10g01617,Vitis10g01618,Vitis10g01619,Vitis10g01620,Vitis10g01621,Vitis10g01622,Vitis10g01624,Vitis10g01625,Vitis10g01626,Vitis10g01627,Vitis10g01628,Vitis10g01629,Vitis10g01635,Vitis10g01636,Vitis10g01637,Vitis10g01639,Vitis10g01640,Vitis10g01641,Vitis10g01646,Vitis10g01647,Vitis10g01648,Vitis10g01649,Vitis10g01650,Vitis10g01651,Vitis10g01652,Vitis10g01653,Vitis10g01654,Vitis10g01655,Vitis10g01656,Vitis10g01657,Vitis10g01658,Vitis10g01659,Vitis10g01662,Vitis10g01663,Vitis10g01664,Vitis10g01665,Vitis10g01666,Vitis10g01670,Vitis10g01671,Vitis10g01672,Vitis10g01673,Vitis10g01674,Vitis10g01675,Vitis10g01676,Vitis10g01677,Vitis10g01678,Vitis10g01679,Vitis10g01680,Vitis10g01681, |
| Chr11 | 7100000-7800000 | Vitis11g00770,Vitis11g00771,Vitis11g00772,Vitis11g00773,Vitis11g00774,Vitis11g00775,Vitis11g00776,Vitis11g00777,Vitis11g00778,Vitis11g00779,Vitis11g00780,Vitis11g00781,Vitis11g00784,Vitis11g00785,Vitis11g00786,Vitis11g00787,Vitis11g00788,Vitis11g00789,Vitis11g00790,Vitis11g00791,Vitis11g00793,Vitis11g00794,Vitis11g00795,Vitis11g00796,Vitis11g00797,Vitis11g00798,Vitis11g00799,Vitis11g00800,Vitis11g00801,Vitis11g00802,Vitis11g00803,Vitis11g00804,Vitis11g00805,Vitis11g00806,Vitis11g00807,Vitis11g00808,Vitis11g00809,Vitis11g00810,Vitis11g00811,Vitis11g00812, |
| Chr16 | 13000000-13500000 | Vitis16g00653,Vitis16g00655,Vitis16g00656,Vitis16g00657,Vitis16g00658,Vitis16g00659,Vitis16g00660,Vitis16g00661,Vitis16g00666,Vitis16g00667,Vitis16g00668, |

**GO enrichment analysis of genes on heterozygous fragments**

| Type | Term | Functions |  | Count | Percent | GeneRatio | PValue | Genes | List Total | Pop Hits | Pop Total | Fold Enrichment | Bonferroni | Benjamini | FDR |
| --- | --- | --- | --- | --- | --- | --- | --- | --- | --- | --- | --- | --- | --- | --- | --- |
| MF | GO:0005515 | protein binding | *** | 117 | 19.69697 | 0.349254 | 4.39E-07 | O48723, Q9LSF1, Q9FFF3, Q8RWH9, Q9FJN9, Q9FKP4, Q9FPR3, Q84J71, Q39193, Q8S2T0, Q9FN50, Q1H5D2, Q9LFT8, Q9M2Z2, Q9SGS5, P46668, F4IH25, O49545, Q94FB9, O49543, Q8VWK4, C0LU16, Q9LSM5, O04479, O64764, Q9M2Y6, Q9XGM2, Q9SDN0, Q9LK43, Q9SJZ7, Q9C778, Q9LVJ0, Q9SRR0, Q9FLH0, Q1H5E9, Q9FJL3, Q9LRR4, Q3EBZ2, F4I2N7, O81001, P18616, F4JRB0, Q39255, Q9FVC1, Q8L540, Q93YZ7, P55852, Q9S9Z2, Q9ZQ34, Q38827, O48716, Q94JY4, Q9SII8, Q9SRS9, Q84TH4, Q39088, Q9SE97, Q0WVX5, Q8GT74, C0LGX3, Q9ASS4, Q84JK2, Q9ASS2, Q6R2J8, F4K265, Q8LES0, Q9FMP0, Q9SA23, Q9ZSA2, Q9SZL8, Q9ZS97, Q9FX45, Q9LIE6, Q9C5Y0, Q9ZVP5, Q67ZB3, Q058P4, Q9SFV2, Q9SWG3, Q9SD11, Q9C5H5, Q9SGP6, Q9SIT9, F4JL28, Q38914, Q9ZPE4, Q9LX14, F4KAB8, C0LGK9, Q05753, Q8H1D6, Q9XIE6, Q8GYN5, Q8H1E4, P46604, Q9LT96, Q6NLW5, Q9LXA5, P48348, Q9SZN7, Q9LTA6, Q9LTA3, Q5XF11, Q9FME2, Q9SZF7, Q38902, Q8GSA7, F4K5J1, Q9SZ66, Q9SV68, Q94F39, Q9FJW6, Q9SGZ8, C0LGT1, Q9SK91, Q6NKN9, P42752 | 335 | 4403 | 19266 | 1.528213 | 1.51E-04 | 1.51E-04 | 1.51E-04 |
| MF | GO:0046872 | metal ion binding | ** | 56 | 9.427609 | 0.167164 | 0.005717717 | Q94BT2, Q9ASS2, Q9SV71, P0C034, Q9FKP5, Q680K2, Q9SCS2, Q9ZU93, Q9SFU3, Q9LSE7, Q8W4C3, Q94HW2, P0CE10, O49543, O65499, Q9LME4, Q9FX85, Q94AM1, Q43872, Q0WVZ1, Q8W468, O80560, Q9S7P5, Q9SJZ2, Q9CA78, Q96326, Q38970, Q9SJZ7, Q9T074, Q8VYN6, F4JL28, Q9XGM8, Q9LSL6, Q9FJP9, F4KAB8, Q0WTB4, Q9LX93, P18616, Q9C5C8, F4JRF4, O81004, Q9ZT94, P0C2F6, Q9SZN7, Q9LTA6, Q8RXX9, Q84TE9, Q0WRW8, O65398, Q42560, P92979, P47924, O80462, Q9SGZ8, P0CB22, Q8GT74 | 335 | 2258 | 19266 | 1.4263 | 0.860097 | 0.618826 | 0.618826 |
| MF | GO:0004672 | protein kinase activity | * | 20 | 3.367003 | 0.059701 | 0.041230905 | Q9FGD7, Q9ASS4, Q8RWL6, Q6R2J8, O49545, Q9LSF1, P43294, C0LGK9, Q9ZVP5, Q9FPR3, F4I2N7, Q9LW83, Q9SCS2, Q39193, Q9ZSA2, Q9LXA5, C0LGT1, Q9LFT8, Q9LK43, Q9C5H5 | 335 | 710 | 19266 | 1.620013 | 0.999999 | 1 | 1 |
| MF | GO:0004674 | protein serine/threonine kinase activity | . | 20 | 3.367003 | 0.059701 | 0.067701006 | Q8RWL6, O49545, Q9LSF1, P43294, Q9SII6, C0LGK9, Q9ZVP5, Q8H1D6, Q9ZR08, Q9FPR3, F4I2N7, Q9LW83, Q9LT96, Q39193, C0LGT1, Q9LK43, Q8RY67, Q9C5H5, Q8GWU0, C0LGX3 | 335 | 755 | 19266 | 1.523456 | 1 | 1 | 1 |
| MF | GO:0008270 | zinc ion binding | . | 19 | 3.198653 | 0.056716 | 0.057966102 | O23169, Q9LSL8, O49543, Q9SYT0, Q9CAI3, Q9LXY5, Q9SY02, P0C899, Q9M2Y7, Q9STF3, Q9C9H9, Q0WN60, Q9FJY7, Q39088, Q8H107, P93011, Q9SZL8, Q9SWG3, Q8LFC0 | 335 | 692 | 19266 | 1.579044 | 1 | 1 | 1 |
| MF | GO:0005525 | GTP binding | * | 11 | 1.851852 | 0.032836 | 0.037515089 | A0A178VBJ0, P47924, O80462, Q38902, O81004, Q9SE83, Q9LFT9, P19892, Q9FJN8, Q8VY57, Q0WTB4 | 335 | 301 | 19266 | 2.101711 | 0.999998 | 1 | 1 |
| MF | GO:0003924 | GTPase activity | . | 8 | 1.346801 | 0.023881 | 0.096825068 | A0A178VBJ0, O80462, Q38902, Q9SE83, Q9LFT9, P19892, Q9FJN8, Q8VY57 | 335 | 225 | 19266 | 2.044816 | 1 | 1 | 1 |
| MF | GO:0016791 | phosphatase activity | ** | 7 | 1.178451 | 0.020896 | 0.007195655 | Q66GQ6, O80560, Q9C5G5, Q9SCS3, Q8GWU0, P0DKC3 | 335 | 98 | 19266 | 4.107889 | 0.916008 | 0.618826 | 0.618826 |
| MF | GO:0043621 | protein self-association | . | 6 | 1.010101 | 0.01791 | 0.074107717 | Q93VH2, Q84JK2, O49545, O49710, Q1H5D2, Q93YZ7 | 335 | 129 | 19266 | 2.674905 | 1 | 1 | 1 |
| MF | GO:0019901 | protein kinase binding | . | 4 | 0.673401 | 0.01194 | 0.085931068 | Q9SV72, Q38902, Q9LSF1, Q9ZVP5 | 335 | 60 | 19266 | 3.83403 | 1 | 1 | 1 |
| MF | GO:0008967 | phosphoglycolate phosphatase activity | *** | 3 | 0.505051 | 0.008955 | 8.89E-04 | Q8GWU0, P0DKC3 | 335 | 3 | 19266 | 57.51045 | 0.262835 | 0.152848 | 0.152848 |
| MF | GO:0004709 | MAP kinase kinase kinase activity | * | 3 | 0.505051 | 0.008955 | 0.023764142 | Q9ZVP5, Q9C5H5, Q9FPR3 | 335 | 14 | 19266 | 12.32367 | 0.999739 | 1 | 1 |
| MF | GO:0015136 | sialic acid transmembrane transporter activity | . | 2 | 0.3367 | 0.00597 | 0.099624492 | Q8GY97, Q8LES0 | 335 | 6 | 19266 | 19.17015 | 1 | 1 | 1 |
| MF | GO:0015165 | pyrimidine nucleotide-sugar transmembrane transporter activity | . | 2 | 0.3367 | 0.00597 | 0.099624492 | Q8GY97, Q8LES0 | 335 | 6 | 19266 | 19.17015 | 1 | 1 | 1 |
| CC | GO:0009507 | chloroplast | * | 94 | 15.82492 | 0.241026 | 0.014096221 | O48723, O48844, Q9LFU1, Q38853, Q38935, Q9SV71, Q8GXB1, O80813, P19892, Q9SW95, Q9CAI3, Q8LES0, Q9SZT4, Q9FKP4, Q680K2, Q84J71, Q8RXE8, Q9LXC9, P93014, Q9FN50, Q0WM29, Q9ZS97, Q9M0Z3, Q8GWU0, Q9LSE7, Q8LDD3, O65451, Q8W4C3, Q94K80, P0CE10, O49543, Q9FX45, Q9LME4, O64764, Q9LSM5, Q94AM1, Q9LW83, Q9SZ46, Q9SCT2, Q9FIW4, F4HUK6, Q9FJY7, Q56XR7, F4HV09, Q9SDN0, Q9SJZ3, Q9CA78, Q9C5H5, Q9SJZ7, Q9SYK9, Q9SVE7, P0DKC3, Q8LFC0, Q9FIF7, O23523, Q9SIT7, Q8VYN6, Q94A73, Q9XEE2, Q9LVJ0, Q9SYT0, Q05753, Q9FLH0, Q0WTB4, Q8W2B8, Q1H5E9, Q9FJP3, Q9STF3, Q9C5C8, Q56X46, O81004, O80470, Q8L540, Q5HZ05, Q93YZ7, Q9FGE9, O65398, Q84WN0, P92979, Q42561, Q9XIB4, Q9SV68, Q9LXY5, Q9SA96, P47924, B0M1H3, Q84TH4, Q9FJW6, Q9LVD9, Q9CAZ7, Q0WVX5, Q8GT74, Q9SHP0 | 390 | 5029 | 26021 | 1.247112 | 0.926624 | 0.324213 | 0.317165 |
| CC | GO:0005886 | plasma membrane | *** | 85 | 14.30976 | 0.217949 | 1.39E-04 | Q94BT2, Q9ASS4, Q9SRT0, Q6R2J8, Q8L7S6, Q9LSF1, Q9ZQ47, Q9FJN7, P19892, Q9FJN8, Q9SZT4, Q9FKP4, Q9FPR3, Q680K2, Q42112, Q9SCS2, Q9LXC9, Q9SVL6, Q9ZSA2, Q9LFT9, Q8LPT3, Q9SGS5, Q8L7W1, Q9LSE7, Q94K80, Q9ZQR4, O49545, Q94A40, F4IJK1, Q9LME4, Q9C5Y0, Q6R8G7, Q940S3, Q9LW83, Q9M2Y6, Q5E930, Q9M2Y3, Q9LK43, Q8RY67, Q9SD11, Q96326, Q9C5H5, Q38970, Q8LFC0, Q9LRB0, Q94A73, Q9SYT0, Q9LX14, C0LGK9, Q9ZR08, Q9XIE6, Q9SX98, Q8GYN5, F4I2N7, Q9LX93, Q9LT96, Q9C5G5, Q56X46, Q9LXA5, Q9SE83, P48348, Q39255, Q9SZN7, Q9LTA6, Q8VY57, Q8RXX9, Q8VYV9, Q8VXX9, Q9LSK9, Q38902, O65398, Q9SU40, Q9SII6, Q42560, Q94JY4, Q9SUM2, O80905, Q9SV68, Q8GUI4, Q6SZ87, O80462, Q6IM84, C0LGT1, Q9SE97, C0LGX3 | 390 | 3818 | 26021 | 1.485396 | 0.025229 | 0.025551 | 0.024995 |
| CC | GO:0005829 | cytosol | ** | 70 | 11.78451 | 0.179487 | 0.00216148 | O48844, Q94BT2, Q9LFU1, Q8RWL6, Q38935, Q9SV71, Q9FFF3, Q9SW95, Q9CAI3, Q9FKP4, Q9FKP5, Q9FPR3, Q42112, Q39193, Q9LXC9, Q9LSV0, Q9ZSA2, Q9LFT9, Q0WM29, Q9LFT8, A2RVV7, Q94A40, O49543, O64764, F4I0P8, Q9LV66, Q94AM1, Q940S3, Q9FIW4, Q9SJZ2, Q9M2Y6, Q9C5H5, Q9T071, Q38970, O23523, Q9T074, O24454, Q9XEE2, Q9SYT0, Q8S8F8, Q8W2B8, Q8H1D6, Q8GYN5, Q9C5G5, Q9LXA8, Q9C5C8, F4JRB0, O81884, Q9SE83, Q9FKZ1, Q8H107, P48348, Q39255, Q8VY57, Q9LTA3, Q93YZ7, Q9FME2, P55852, Q9C5S1, O65398, Q9SU40, Q42560, Q9SUM2, O22822, Q9SV68, Q9FPS4, Q9SII8, Q9SGZ8, Q9CAZ7, Q8RWU7 | 390 | 3286 | 26021 | 1.421313 | 0.328434 | 0.132571 | 0.129689 |
| CC | GO:0009536 | plastid | . | 22 | 3.703704 | 0.05641 | 0.065566109 | Q9FME2, O49543, Q9XEE2, Q9LVJ0, Q9FFF3, Q9FJN8, P92979, Q94AM1, Q9FJL3, Q67ZB3, Q9LT96, Q9FJP3, Q9LV81, Q9LXC9, Q9FJW4, Q39193, Q9FN50, P48348, Q8GWU0, Q8L540, P0DKC3 | 390 | 986 | 26021 | 1.488693 | 0.999996 | 0.857752 | 0.839106 |
| CC | GO:0005773 | vacuole | ** | 21 | 3.535354 | 0.053846 | 0.007477822 | Q94BV5, O65398, Q9SYT0, Q8S8F8, Q9C5Y0, Q9FX85, Q42560, Q43872, Q9FJL3, Q9SV68, Q8RYD1, Q9SIE7, Q9LXA8, P18616, Q9SJZ2, P42814, Q9SA23, Q9LVD9, Q9CAZ7, Q8VY57, Q9SJZ7 | 390 | 732 | 26021 | 1.914113 | 0.748695 | 0.229581 | 0.22459 |
| CC | GO:0000325 | plant-type vacuole | ** | 20 | 3.367003 | 0.051282 | 0.009899597 | Q94BV5, O65398, Q9SU40, Q7XA86, Q9SYT0, Q9C5Y0, Q42560, Q9FJL3, Q9SV68, Q8RYD1, Q9LXA8, P18616, Q9M2Y6, P42814, Q9SA23, Q9SE83, Q9SVL6, Q9LVD9, Q8VY57, P0DKC3 | 390 | 703 | 26021 | 1.898165 | 0.839683 | 0.260218 | 0.254561 |
| CC | GO:0009570 | chloroplast stroma | . | 17 | 2.861953 | 0.04359 | 0.078304367 | Q9SYT0, Q84WN0, P92979, Q94AM1, Q680K2, Q9SA96, Q9FXB6, P47924, Q9FJP3, Q9C5C8, Q9LXC9, P93014, Q9FN50, Q9ZS97, P0DKC3, Q9SHP0 | 390 | 722 | 26021 | 1.570982 | 1 | 0.857752 | 0.839106 |
| CC | GO:0000139 | Golgi membrane | ** | 15 | 2.525253 | 0.038462 | 0.005236467 | Q9LSG3, Q9SRT3, Q94A40, Q9XGM8, Q3EC11, Q9FFF3, Q8LES0, Q9XIE6, Q9FFN2, A8MRC7, Q9M2Y6, O81007, Q9SE83, Q9LFT9, Q9M0Z3 | 390 | 426 | 26021 | 2.349314 | 0.619413 | 0.229581 | 0.22459 |
| CC | GO:0043231 | intracellular membrane-bounded organelle | ** | 12 | 2.020202 | 0.030769 | 0.001637878 | Q9SY02, P0C899, O23169, Q9LRB0, Q9SIT7, Q9STF3, Q38902, Q9ZQR4, Q940V3, Q9LJR6, P93011, Q9LTA3 | 390 | 254 | 26021 | 3.15215 | 0.260378 | 0.132571 | 0.129689 |
| CC | GO:0005802 | trans-Golgi network | * | 11 | 1.851852 | 0.028205 | 0.02164517 | F4HYR4, Q9LSG3, Q9SRT3, O81007, Q9M2Y6, Q8S2T0, Q9XGM8, Q9S7J8, Q6R8G7, Q9XIE6, Q9FPR3 | 390 | 317 | 26021 | 2.315223 | 0.982162 | 0.442523 | 0.432903 |
| CC | GO:0005768 | endosome | . | 10 | 1.683502 | 0.025641 | 0.072827512 | F4HYR4, Q9LSG3, Q9SRT3, O81007, Q9M2Y6, Q9XGM8, Q9S7J8, Q9FJN8, Q9XIE6, Q9FPR3 | 390 | 341 | 26021 | 1.956613 | 0.999999 | 0.857752 | 0.839106 |
| CC | GO:0012505 | endomembrane system | * | 7 | 1.178451 | 0.017949 | 0.025556561 | Q9SA23, Q9FKZ1, Q9LFT9, P19892, Q8L709, Q8H1D6, Q8GYN5 | 390 | 150 | 26021 | 3.113624 | 0.991465 | 0.470241 | 0.460018 |
| CC | GO:0005622 | intracellular | . | 5 | 0.841751 | 0.012821 | 0.072853634 | P42814, Q9SRR0, F4I0P8, Q9SZT4, Q9FKP4 | 390 | 105 | 26021 | 3.177167 | 0.999999 | 0.857752 | 0.839106 |
| CC | GO:0000785 | chromatin | . | 4 | 0.673401 | 0.010256 | 0.087403501 | Q8VWK4, Q9XGM2, P0CB22, F4KAB8 | 390 | 70 | 26021 | 3.812601 | 1 | 0.857752 | 0.839106 |
| CC | GO:0005819 | spindle | . | 4 | 0.673401 | 0.010256 | 0.096203594 | Q38902, Q39255, Q8VY57, Q0WQE7 | 390 | 73 | 26021 | 3.655919 | 1 | 0.857752 | 0.839106 |
| CC | GO:0035619 | root hair tip | ** | 3 | 0.505051 | 0.007692 | 0.007486339 | Q9C5G5, Q9SYT0, F4K5J1 | 390 | 9 | 26021 | 22.24017 | 0.749092 | 0.229581 | 0.22459 |
| CC | GO:0031902 | late endosome membrane | . | 3 | 0.505051 | 0.007692 | 0.057234164 | Q9ASS2, Q9FLG8, Q8VY57 | 390 | 26 | 26021 | 7.698521 | 0.99998 | 0.857752 | 0.839106 |
| CC | GO:0030173 | integral component of Golgi membrane | . | 3 | 0.505051 | 0.007692 | 0.096098517 | Q8GY97, Q8VXX9, Q8LES0 | 390 | 35 | 26021 | 5.718901 | 1 | 0.857752 | 0.839106 |
| CC | GO:0055037 | recycling endosome | . | 2 | 0.3367 | 0.005128 | 0.058473546 | Q9SZF7, Q9XIE6 | 390 | 4 | 26021 | 33.36026 | 0.999985 | 0.857752 | 0.839106 |
| BP | GO:0009414 | response to water deprivation | . | 22 | 3.703704 | 0.060274 | 0.094863496 | Q9SIT9, Q9XEE2, F4IJK1, Q9SYT0, Q9FJN7, P0C034, Q9SII6, Q9LV66, Q9SV68, Q9FPR3, Q8H1E4, P46604, Q9M2Y9, Q94F39, Q39193, Q940V3, Q5E930, Q6IM84, Q9SVL6, Q9T071, Q9LTA3, P46668 | 365 | 955 | 22556 | 1.423602 | 1 | 1 | 1 |
| BP | GO:0006468 | protein phosphorylation | . | 19 | 3.198653 | 0.052055 | 0.056850196 | Q9FGD7, Q9ASS4, Q8RWL6, Q6R2J8, Q9LSF1, Q9SII6, C0LGK9, Q8H1D6, Q9ZR08, Q8GYN5, Q9LW83, Q9LT96, Q39193, C0LGT1, Q9LFT8, Q9LK43, Q8RY67, Q8GWU0, C0LGX3 | 365 | 740 | 22556 | 1.586686 | 1 | 1 | 1 |
| BP | GO:0009738 | abscisic acid-activated signaling pathway | *** | 16 | 2.693603 | 0.043836 | 3.34E-05 | O23523, Q9SRT0, Q94K80, Q38902, Q9C778, Q9ZQ47, Q9SII6, Q9ZVP5, Q9FPR3, P46604, Q9M2Y9, Q94F39, Q39193, Q6IM84, Q9MAH1, P46668 | 365 | 268 | 22556 | 3.689389 | 0.024396 | 0.024665 | 0.024665 |
| BP | GO:0006979 | response to oxidative stress | * | 16 | 2.693603 | 0.043836 | 0.010802521 | Q38853, Q9LSF1, Q9C778, Q9SYT0, Q9FX85, Q43872, F4I2N7, Q9FKQ1, Q9SIE7, Q9C5C8, Q9SJZ2, Q9LSV0, Q9LVD9, Q0WM29, Q9SGS5, Q9T071 | 365 | 476 | 22556 | 2.077219 | 0.999677 | 1 | 1 |
| BP | GO:0009651 | response to salt stress | * | 16 | 2.693603 | 0.043836 | 0.037885783 | O23523, Q9C5S1, O49710, Q7XA86, Q9XEE2, Q9SYT0, Q9SRS9, Q8H1E4, Q9FKQ1, Q94F39, Q39193, Q5E930, Q9MAH1, Q8RY67, Q9SGS5, Q9T071 | 365 | 557 | 22556 | 1.775146 | 1 | 1 | 1 |
| BP | GO:0051301 | cell division | * | 14 | 2.356902 | 0.038356 | 0.043302152 | Q9FJK7, Q94A73, Q9SXY0, Q9C778, Q38914, Q9LX14, F4K5J1, Q8W1Y0, Q0WQE7, Q9SIE7, F4HYR4, F4JRF4, Q8VY57, P42752 | 365 | 471 | 22556 | 1.836861 | 1 | 1 | 1 |
| BP | GO:0006970 | response to osmotic stress | . | 11 | 1.851852 | 0.030137 | 0.054809498 | Q8H1E4, O23523, Q9FJW4, Q39193, Q9ZQ47, Q9SYT0, Q9MAH1, Q9ZVP5, Q9T071, Q9LTA3, Q8L7W1 | 365 | 345 | 22556 | 1.970347 | 1 | 1 | 1 |
| BP | GO:0006511 | ubiquitin-dependent protein catabolic process | . | 11 | 1.851852 | 0.030137 | 0.05993685 | O48844, Q8W468, Q9SRR1, O24454, Q9ZPE4, Q9LVD9, Q39255, O22207, Q9FKP5, Q9FPS4, Q9SII8 | 365 | 351 | 22556 | 1.936666 | 1 | 1 | 1 |
| BP | GO:0046686 | response to cadmium ion | . | 10 | 1.683502 | 0.027397 | 0.088336352 | O48723, Q9T074, O65398, Q9LXC9, P93014, Q9SYT0, Q39255, Q5HZ05, Q42112, Q9SHP0 | 365 | 330 | 22556 | 1.872644 | 1 | 1 | 1 |
| BP | GO:0044085 | cellular component biogenesis | * | 8 | 1.346801 | 0.021918 | 0.049215225 | Q8LER3, F4HYR4, Q9M2Y7, O65399, P0CB23, Q9SJZ3, Q9SFV9, Q9SW44 | 365 | 205 | 22556 | 2.4116 | 1 | 1 | 1 |
| BP | GO:0009408 | response to heat | . | 8 | 1.346801 | 0.021918 | 0.065424446 | Q8H1E4, Q9SIT9, P55852, O49710, Q9XEE2, Q9SYT0, Q9T071, Q9SJZ7 | 365 | 219 | 22556 | 2.257434 | 1 | 1 | 1 |
| BP | GO:0009451 | RNA modification | ** | 7 | 1.178451 | 0.019178 | 0.005400046 | Q9SY02, P0C899, O23169, Q9SIT7, Q9STF3, Q9LJR6, P93011 | 365 | 99 | 22556 | 4.369503 | 0.98181 | 0.798127 | 0.798127 |
| BP | GO:1905392 | plant organ morphogenesis | ** | 7 | 1.178451 | 0.019178 | 0.009698313 | Q8W4C3, Q94A73, Q8L627, Q38914, Q9LX14, Q52QU2, Q0WRW8 | 365 | 112 | 22556 | 3.862329 | 0.999262 | 1 | 1 |
| BP | GO:0010052 | guard cell differentiation | ** | 4 | 0.673401 | 0.010959 | 0.002395739 | Q9SV72, Q9T068, Q9LSL6, Q9LFT5 | 365 | 17 | 22556 | 14.54053 | 0.830512 | 0.798127 | 0.798127 |
| BP | GO:0061077 | chaperone-mediated protein folding | ** | 4 | 0.673401 | 0.010959 | 0.003875141 | Q38935, Q9FN50, Q9SDN0, Q9FJL3 | 365 | 20 | 22556 | 12.35945 | 0.943481 | 0.798127 | 0.798127 |
| BP | GO:1900865 | chloroplast RNA modification | ** | 4 | 0.673401 | 0.010959 | 0.00511163 | Q9FIF7, Q9FJY7, Q9SJZ3, Q9LXY5 | 365 | 22 | 22556 | 11.23587 | 0.977456 | 0.798127 | 0.798127 |
| BP | GO:0009789 | positive regulation of abscisic acid-activated signaling pathway | . | 4 | 0.673401 | 0.010959 | 0.054034313 | Q39193, Q9MAH1, Q9C5Y0, Q9ZVP5 | 365 | 53 | 22556 | 4.663944 | 1 | 1 | 1 |
| BP | GO:0006099 | tricarboxylic acid cycle | . | 4 | 0.673401 | 0.010959 | 0.069966517 | Q8H107, Q9FJP9, Q42560, Q8LFC0 | 365 | 59 | 22556 | 4.189645 | 1 | 1 | 1 |
| BP | GO:0007140 | male meiosis | * | 3 | 0.505051 | 0.008219 | 0.023737908 | B0M1H3, Q6NLW5, Q39255 | 365 | 15 | 22556 | 12.35945 | 1 | 1 | 1 |
| BP | GO:0007264 | small GTPase mediated signal transduction | * | 3 | 0.505051 | 0.008219 | 0.030103538 | A0A178VBJ0, Q38902, Q9LXC0 | 365 | 17 | 22556 | 10.9054 | 1 | 1 | 1 |
| BP | GO:0017004 | cytochrome complex assembly | * | 3 | 0.505051 | 0.008219 | 0.040749139 | Q96326, P93280, P92527 | 365 | 20 | 22556 | 9.269589 | 1 | 1 | 1 |
| BP | GO:0009932 | cell tip growth | . | 3 | 0.505051 | 0.008219 | 0.061060254 | Q66GQ6, Q9C5G5, Q9SU40 | 365 | 25 | 22556 | 7.415671 | 1 | 1 | 1 |
| BP | GO:0046856 | phosphatidylinositol dephosphorylation | . | 3 | 0.505051 | 0.008219 | 0.069967907 | Q66GQ6, O80560, Q9C5G5 | 365 | 27 | 22556 | 6.866362 | 1 | 1 | 1 |
| BP | GO:0010584 | pollen exine formation | . | 3 | 0.505051 | 0.008219 | 0.093880175 | Q06915, O49432, Q8LES0 | 365 | 32 | 22556 | 5.793493 | 1 | 1 | 1 |
| BP | GO:1905157 | positive regulation of photosynthesis | . | 2 | 0.3367 | 0.005479 | 0.063008748 | Q9M2Y6, Q9M0Z3 | 365 | 4 | 22556 | 30.89863 | 1 | 1 | 1 |
| BP | GO:0045787 | positive regulation of cell cycle | . | 2 | 0.3367 | 0.005479 | 0.078132232 | Q9SXY0, Q39088 | 365 | 5 | 22556 | 24.7189 | 1 | 1 | 1 |
| BP | GO:0007276 | gamete generation | . | 2 | 0.3367 | 0.005479 | 0.078132232 | F4IH25, Q38914 | 365 | 5 | 22556 | 24.7189 | 1 | 1 | 1 |
| BP | GO:0015739 | sialic acid transport | . | 2 | 0.3367 | 0.005479 | 0.093012276 | Q8GY97, Q8LES0 | 365 | 6 | 22556 | 20.59909 | 1 | 1 | 1 |
